# Structural Insights Into Man_9_ Recognition by the HIV Antibody 2G12 Revealed by Paramagnetic NMR

**DOI:** 10.1002/advs.202600025

**Published:** 2026-04-22

**Authors:** Adrián Silva‐Díaz, Paola Oquist‐Phillips, Noelia de la Cruz, Inés Lera‐Lasso, Laura Castañar, Javier Rojo, Javier Ramos‐Soriano, Ángeles Canales

**Affiliations:** ^1^ Glycosystems Laboratory Instituto de Investigaciones Químicas (IIQ) CSIC – Universidad de Sevilla Seville Spain; ^2^ Department of Organic Chemistry Faculty of Chemistry Universidad Complutense de Madrid Madrid Spain

**Keywords:** 2G12 antibody, lanthanide, Man_9_, Mannose, NMR

## Abstract

Viral proteins contain “glycoepitopes” that can be targeted by antibodies. A prominent example is HIV gp120 glycoprotein, which contains a dense cluster of oligomannoside glycans recognized by the broadly neutralizing 2G12 antibody. 3D structures of 2G12 complexed with oligomannosides of diverse sizes have been determined using X‐ray crystallography and cryo‐electron microscopy, and different glycan binding epitopes have been proposed, with either the D1 arm or the D3 arm of the glycans establishing the main interactions with the antibody. In this work, we have synthesized a Man_9_ derivative bearing a lanthanide binding tag to perform detailed characterization of the glycan binding epitope in solution using NMR under paramagnetic conditions. This approach allows studying the Man_9_ structure, overcoming the signal overlapping problems found in standard NMR experiments. STD NMR analysis revealed a clear preference for the D1 arm in 2G12 antibody recognition, while the D2 and D3 branches also contributed to binding, albeit with weaker STD effects. This relevant information is fundamental for the design of immunogens to elicit 2G12‐like antibodies as vaccine components, since recent studies have found that these “glycoepitopes” are also present in other viruses such as SARS‐CoV‐2 and certain H3N2 influenza strains.

## Graphical abstract

The HIV gp120 glycoprotein displays Man_9_ oligomannose structures that are recognized by the neutralizing antibody 2G12. Detailed characterization of the binding epitope in solution is important for immunogen design but remains challenging, since the signals of the terminal mannoses from the three arms of Man_9_ (D1, D2, and D3) cannot be distinguished in conventional experiments. Herein, this problem is overcome using a paramagnetic approach.

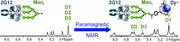

## 1 Introduction

Viral infections pose a major threat to global public health, as dramatically demonstrated by the COVID‐19 outbreak. It is well established that glycans attached to glycoproteins decorating the surface of both host cells and viruses play a crucial role in viral infections [1]. One common viral strategy to evade the human immune system is the development of a glycan shield to mask protein epitopes. However, certain protein regions with specific glycosylation patterns, particularly oligomannoside structures, remain exposed and accessible for interactions with human receptors, mainly DC‐SIGN (expressed on dendritic cells) and L‐SIGN (located on endothelial cells) [2]. These glycosylation sites are emerging as new “glycoepitopes” that can be targeted by antibodies [3]. The best‐known example is the HIV gp120 glycoprotein, which contains a dense cluster of glycosylation sites forming what has been termed the “silent face” of the viral protein. Remarkably, HIV‐infected patients have developed unique antibodies, such as 2G12, capable of targeting this glycan‐rich surface [4]. This antibody represents a paradigm in glycan recognition, as it is one of the few known human antibodies that neutralizes HIV by specifically targeting the glycan component of the viral glycoprotein. Recent studies point out that 2G12 can also recognize glycans on SARS‐CoV‐2 and on certain H3N2 influenza viruses, raising the possibility of repurposing this antibody for new applications [3].

2G12 adopts a domain‐swapped conformation with two Fabs that exchange their variable heavy chain domains, creating two primary glycan‐binding sites at the interface of the light and heavy chains and two potential secondary binding sites at the interface of the heavy chains [4]. This antibody specifically targets high‐mannose‐type oligosaccharides within the HIV gp120 glycoprotein, with a particular affinity for the dense cluster of high‐mannose *N*‐glycans located in the C3/V4 domain of gp120 [5]. Since its discovery, extensive efforts have focused on determining how 2G12 recognizes epitopes within gp120 and on enhancing its ability to neutralize diverse HIV strains.

**FIGURE 1:**
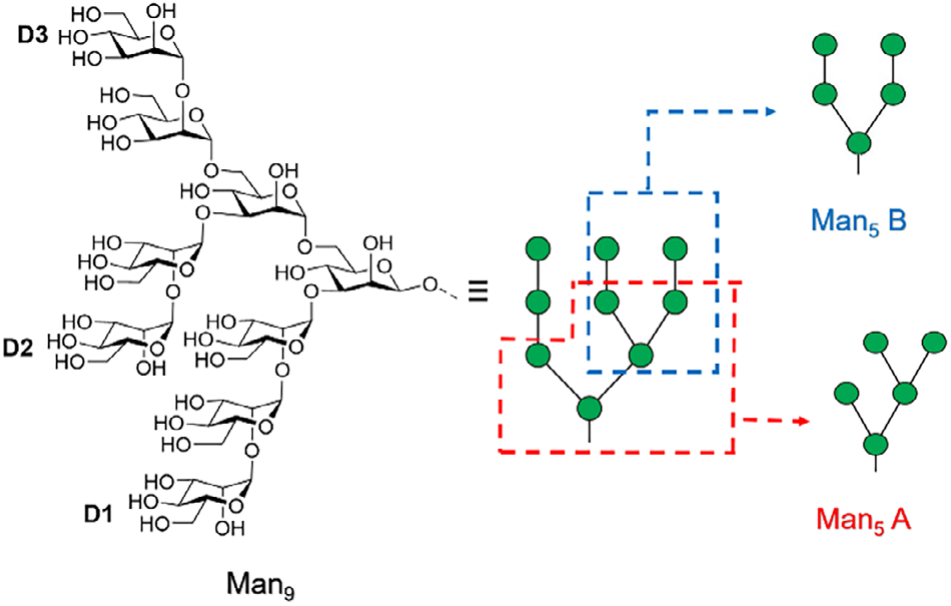
Structures of Man_9_ and the pentamannosides Man_5_ A and Man_5_ B. Structures were schematically represnted using the symbol nomenclature for glycans (SNFG) [19].

X‐ray crystal structures of 2G12 in complex with different oligomannosides (Man_4_, Man_5_, Man_7_, Man_8_, and Man_9_) have been solved, revealing distinct glycan‐recognition modes. In these complexes, the antibody shows a preferential interaction with the terminal Manα1,2Man moiety located on the D1 and D3 arms (Figure 1). However, whether D1 or D3 occupies the primary binding site depends on the particular oligomannoside. The crystal structures of Man_4_, Man_7_, and Man_9_ show that 2G12 interacts primarily with the glycan's D1 arm [5]. In contrast, the Man_5_ and Man_8_ structures indicate that the D3 arm can also occupy the primary binding site [4, 6]. In addition, the cryo‐EM structure of the SARS‐CoV‐2 spike protein bound to the 2G12 antibody also shows that the D3 arm of one of the glycans attached to the spike protein is positioned within the antibody's primary binding site [7]. In this context, further research is needed to clarify the differences observed in the reported structures. Furthermore, a significant gap remains in our understanding of the recognition process in solution. Addressing this gap is essential for achieving a comprehensive view of the interaction mechanism and for guiding the design of more effective therapeutics capable of targeting a broader spectrum of HIV strains. In this line, NMR is particularly suited to characterize protein‐glycan interactions in solution [8] as shown in previous studies [9, 10], describing the interaction of 2G12 with small mannose oligosaccharides (up to pentasaccharide).

However, the extensive signal overlap in NMR spectra of intact Man_9_ has thus far precluded a detailed atomic‐level characterization of its binding epitope. To overcome this limitation, a lanthanide‐binding tag (LBT) can be attached to the oligosaccharide. The incorporation of paramagnetic ions into the LBT increases signal dispersion in NMR spectra [11], thereby facilitating structural analysis.

In previous work, we successfully applied this approach to carry out conformational analyses of biantennary [11] and tetraantennary complex‐type *N*‐glycans [12], as well as to characterize the recognition of sialylated *N‐*glycans by influenza hemagglutinin [13]. Similarly, Kato's group elegantly applied this methodology to investigate the conformational dynamics of oligomannosides [14]. Herein, we synthesized a strategically conjugated Man_9_ derivative bearing an LBT at the reducing end and applied the paramagnetic approach to demonstrate the advantages of LBT‐conjugated oligomannosides for detailed binding studies with 2G12. This strategy enables resolution of signals corresponding to individual mannose units in NMR spectra and, for the first time, allows detailed characterization of the Man_9_ binding epitope in complex with 2G12 in solution. Moreover, the method can be broadly applied to characterize glycan epitopes recognized by emerging glycan‐targeting antibodies, with direct implications for structure‐based vaccine design.

## 2 Results and Discussion

### 2.1 Man_5_ and Man_9_/2G12 Binding Studies

The glycosylation pattern of gp120 produced in different systems has been characterized in previous works. Initial reports demonstrated that the trimeric envelope glycoprotein derived from pseudoviral systems was almost entirely glycosylated with oligomannosides, with Man_5_ structures representing the predominant species [15]. Subsequently, a wider range of viral production systems was examined, and a broader distribution of glycan abundances was reported; although in most systems, Man_5_ and Man_9_ glycans were present in high proportions [16]. It is worth noting that different Man_5_ structures can be considered. For instance, the Man_5_ A oligomannoside (Figure 1) represents a biosynthetic intermediate in the Man_9_ processing pathway and is naturally present in gp120, whereas the Man_5_ B glycan (Figure 1) corresponds to a fragment of Man_9_ comprising two of its arms (D2 and D3). Man_5_ B has been used as a model system in structural studies, but it is not naturally found in gp120. Man_5_ A glycan has been identified in recombinant gp120, in HIV virions produced in HEK293T cells, and in viruses generated by peripheral blood mononuclear cell line (PBMC) infection [15, 16]. Additionally, the Man_5_ A structure is recognized by another glycan‐targeting gp120 antibody PG9 [17]. However, the recognition of Man_5_ A oligomannoside by 2G12 remains poorly characterized, and structural data for both Man_5_ A and Man_9_ oligosaccharides in complex with 2G12 in solution are completely lacking. To date, only two X‐ray crystal structures have been reported: 2G12 bound to a Man_5_ B oligosaccharide (PDB: 6MUB) [6] and 2G12 in complex with Man_9_ (PDB: 6N2X) [4].

**FIGURE 2:**
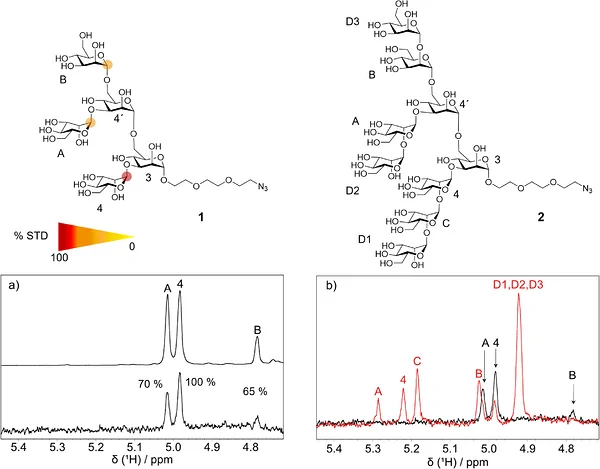
Binding studies of the Man_5_ **1** to 2G12 and competition studies with Man_9_ **2**. (a) Expansion of the anomeric region of (top) off resonance and (bottom) STD ^1^H NMR spectra of Man_5_ **1** (0.6 mM) in the presence of the 2G12 antibody (12 µM); Man_5_:2G12, 50:1 molecular ratio. Normalized levels of saturation (%) measured for each signal are shown in the bottom spectrum and highlighted in the structure using a colour code. (b) Expansion of the anomeric region of the ^1^H STD NMR spectra of Man_5_ **1** in the presence of 2G12 (in black) and of the Man_5_/2G12 sample upon addition of Man_9_ **2** (in red) to a final concentration of 0.6 mM, Man_5_:Man_9_:2G12, 50:50:1 molecular ratio. The anomeric region is shown for the Man_5_/Man_9_ comparison, as it is the region with the least signal overlap. Full NMR spectra are given in the Supporting Information, Section 4, Figures S32 and S33.

In this context, as an initial step, we performed ^1^H saturation transfer difference NMR (^1^H STD NMR) [18] experiments to evaluate the recognition of Man_5_ A and Man_9_ glycans by 2G12, since both are found in gp120. For these studies, Man_5_ **1** and Man_9_ **2** (Figure 2) oligomannoside derivatives were synthesized.

**SCHEME 1:**
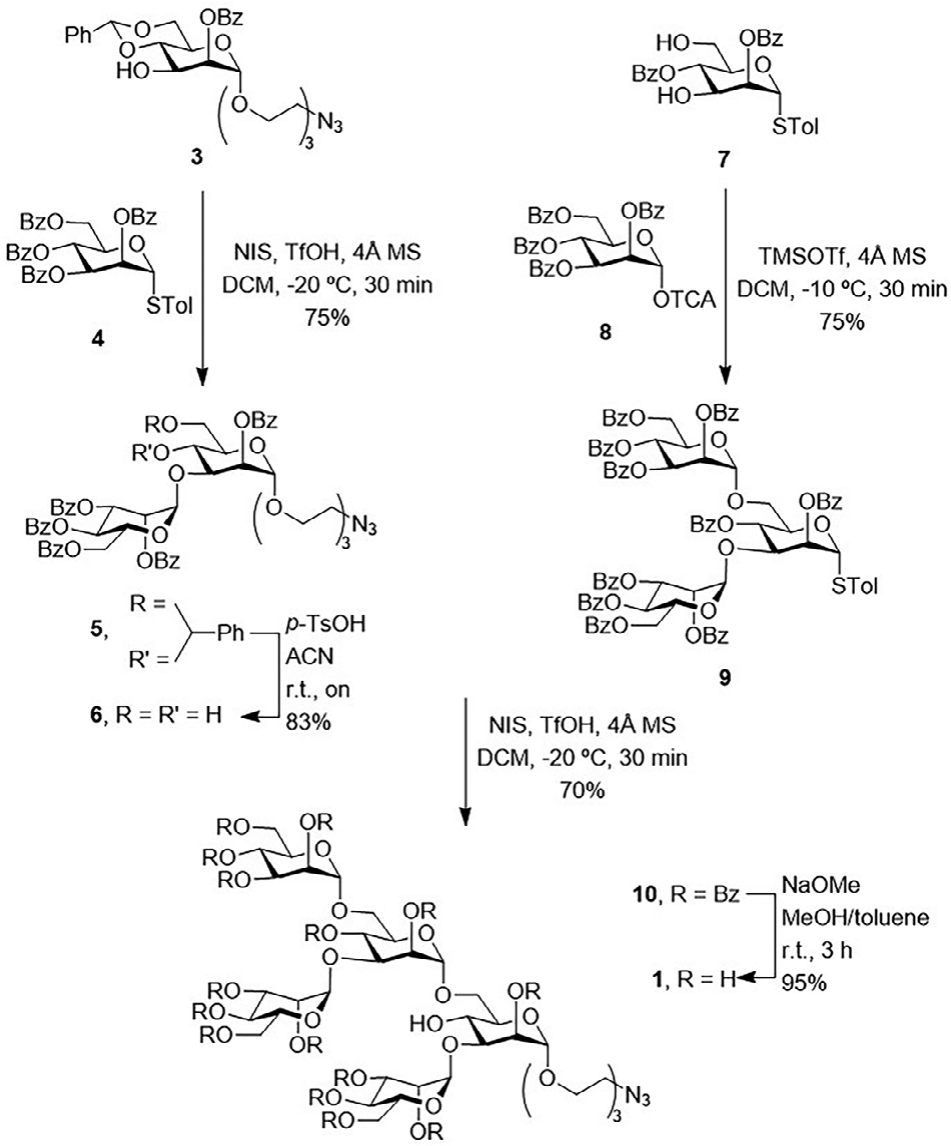
Synthesis of the Man_5_ oligosaccharide **1**.

Man_9_ **2** synthetic procedure was previously described by our group [20]. The synthesis of the pentamannoside **1** (Scheme 1) started with the glycosylation of acceptor **3** [21] with donor **4** [22] promoted by NIS/TfOH to afford disaccharide **5**. After selective benzylidene cleavage, disaccharide **6** was successfully glycosylated with trisaccharide **9**, which was obtained by 2 + 1 glycosylation of acceptor **7** [23] with donor **8** [24] in good yield. Finally, compound **10** was subjected to Zemplén deprotection, affording Man_5_ **1** in excellent yield.

Once Man_5_ **1** was synthesized, its recognition by 2G12 was evaluated by using ^1^H STD NMR experiments. Clear ^1^H STD signals were observed for the pentasaccharide Man_5_ **1** in the presence of 2G12 (Figure 2a), indicating that the glycan is recognized by the antibody. Therefore, 2G12 is able to recognize truncated oligomannosides containing only α(1‐3) and α(1‐6) linkages. The strongest effects were observed for the protons located at mannose 4 (Figure 2a).

In the next step, competition experiments with Man_9_ were performed. Man_9_ **2** was subsequently added to the NMR tube containing both Man_5_ **1** and the antibody, and a new ^1^H STD NMR experiment was acquired. Under these conditions, the ^1^H STD signals of Man_5_ **1** decreased (Figure 2b), and strong ^1^H STD signals were observed for Man_9_ **2**.

This result points out that Man_9_ **2** binds more effectively as a ligand than Man_5_ **1**, as expected, since 2G12 preferentially recognizes Manα1‐2Man epitopes. This result is in agreement with the relative affinity obtained for similar Man_5_ and Man_9_ structures by competitive inhibition of 2G12 binding to immobilized gp120 [25]. Therefore, it is essential to characterize in detail the recognition of Man_9_ **2** by 2G12 in solution to define the binding epitope.

**SCHEME 2:**
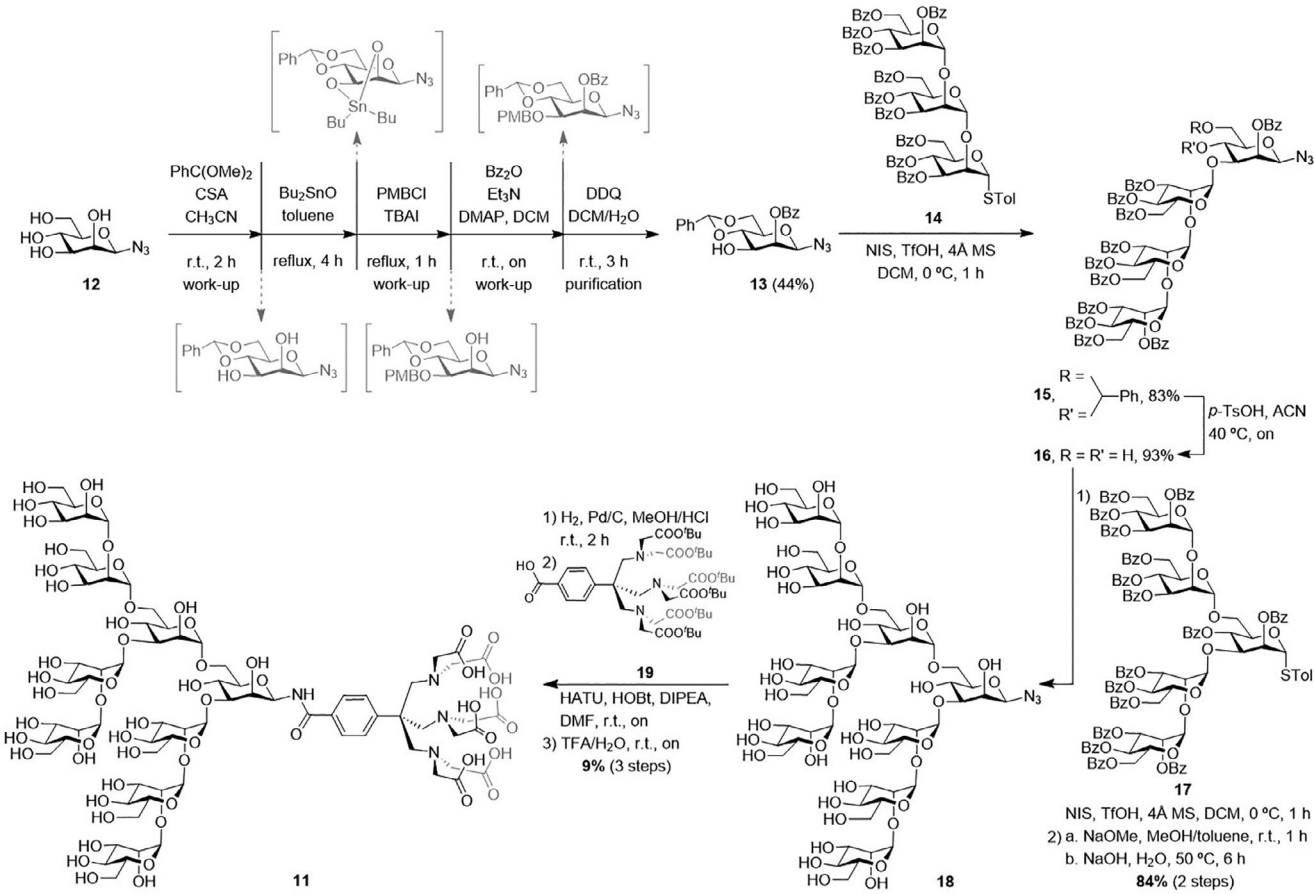
Synthesis of LBT‐Man_9_ oligosaccharide **11**.

However, in standard ^1^H NMR experiments, the signals of the terminal mannoses on the D1, D2, and D3 arms cannot be individually identified, as they overlap due to their similar chemical environments (Figure 2b). To overcome this problem, a Man_9_ derivative bearing an LBT (Man_9_‐LBT **11**) was synthesized (Scheme 2). A phenyl‐triaminohexaacetate (Ph‐TAHA) structure was used as LBT [26]. The key features considered in the selection of this chelating unit are the C3‐symmetry, which ensures the observation of only one set of signals upon lanthanide coordination, and the presence of nine coordination sites that form stable complexes with lanthanide ions.

We envisioned the synthesis of the Man_9_‐LBT conjugate **11** (Scheme 2) based on our previously established convergent and straightforward synthetic strategy for obtaining complex oligomannosides [20, 23, 27]. Initially, mannose **12** [28] was orthogonally protected using both stepwise (for further details, see Section S2 of the Supporting Information) and consecutive approaches. In brief, the chemoselective protection of C‐4 and C‐6 as a benzylidene acetal was followed by regioselective *p*‐methoxybenzylation of C‐3 via the initial formation of a tin acetal between C‐2 and C‐3, subsequent benzoylation of C‐2, and finally, the selective removal of the *p*‐methoxybenzyl ether group. This synthetic five‐step sequence provided the desired acceptor **13** with an overall yield of 29 % using the stepwise approach (including purification of all intermediates) and 44 % using the consecutive approach (with only a final chromatographic purification) from mannose **12**.

The glycosylation reaction of compound **13** with donor **14** [23] in the presence of NIS and TfOH as glycosylation promoters yielded benzylidene‐protected tetrasaccharide **15**, which was deprotected in acidic media to afford tetrasaccharide **16** in high yield.

Compound **16** was then subjected to a glycosylation reaction with pentasaccharide **17**, previously described in the literature [23], followed by global deprotection using basic conditions, yielding nonasaccharide **18** with 84 % overall yield (over 2 steps).

The target molecule **11**, featuring an aglycone LBT at the reducing end, was obtained in moderate yield by hydrogenation of azido **18** to the corresponding amino derivative, followed by conjugation with carboxylic acid‐equipped LBT **19** [26] via HATU‐mediated coupling, and finally, deprotection of *tert*‐butyl esters in acidic media.

The β‐stereochemistry of the anomeric center of compound **11** was confirmed by the ^1^ *J*_C‐1,H‐1_ coupling constant (^1^ *J*_CH_ = 158.7 Hz), determined via a coupled 2D ^1^H‐^13^C HSQC NMR experiment (see Figure S27 in Section 3 of the Supporting Information).

### 2.2 LBT‐Man_9_ 11 Conformational Analysis

Following the synthesis of LBT‐Man_9_ **11**, NMR characterization and conformational analysis of the structure were performed. The use of glycan conjugates bearing LBTs, which chelate paramagnetic metal ions, allows measuring NMR parameters with valuable structural information, such as pseudo‐contact shift (PCS), which arises from the dipolar interaction of the nuclei with the paramagnetic center. The PCS depends on the distance of a nucleus to the metal and on the shape and orientation of the metal's paramagnetic susceptibility tensor [29]. The experimental PCS determination requires comparison of the chemical shift of each signal under diamagnetic and paramagnetic conditions. The first step in the NMR characterization was the acquisition of a 2D ^1^H‐^13^C HSQC spectrum of the glycan derivative **11** loaded with La^3+^ (diamagnetic reference) and Dy^3+^ (paramagnetic metal).

**FIGURE 3:**
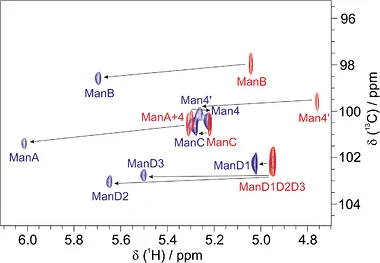
Expansion of the anomeric region of the 2D ^1^H–^13^C HSQC spectrum of LBT‐Man_9_ **11** loaded with lanthanum (diamagnetic reference, in red) overlaid with the spectrum of **11** loaded with dysprosium (paramagnetic ion, in blue). Residual signals of unbound glycan were observed in the dysprosium spectrum at low intensity level, as the weak signal (in blue) below the signal of the protons H1 D1, H1 D2 and H1 D3 in the red spectrum. However, no additional dysprosium was added to the sample to avoid nonspecific line broadening caused by metal excess. Full spectra are provided in the Supporting Information, Section 6 (Figure S35).

Clear PCSs were detected in the presence of dysprosium, as shown in Figure 3. The most significant chemical shift change was detected for H‐1 of the β‐mannose linked to the LBT, with a PCS value of 1.12 ppm. It is remarkable that the anomeric protons of mannoses D1, D2, and D3, normally indistinguishable in standard NMR experiments, could be individually identified in the presence of dysprosium (Figure 3).

A total of 20 PCSs were measured for the LBT(Dy^3+^)‐Man_9_ complex (the PCS value is the difference in ^1^H chemical shift of each signal in diamagnetic and paramagnetic conditions) and were used to investigate the conformational behavior of the glycan. This was achieved by comparing experimental PCS values with those estimated by back‐calculating the expected PCS values for the individual energy minima of the ensembles obtained from molecular dynamics simulations, using MSpin software [30, 31] as described in previous work [12].

**FIGURE 4:**
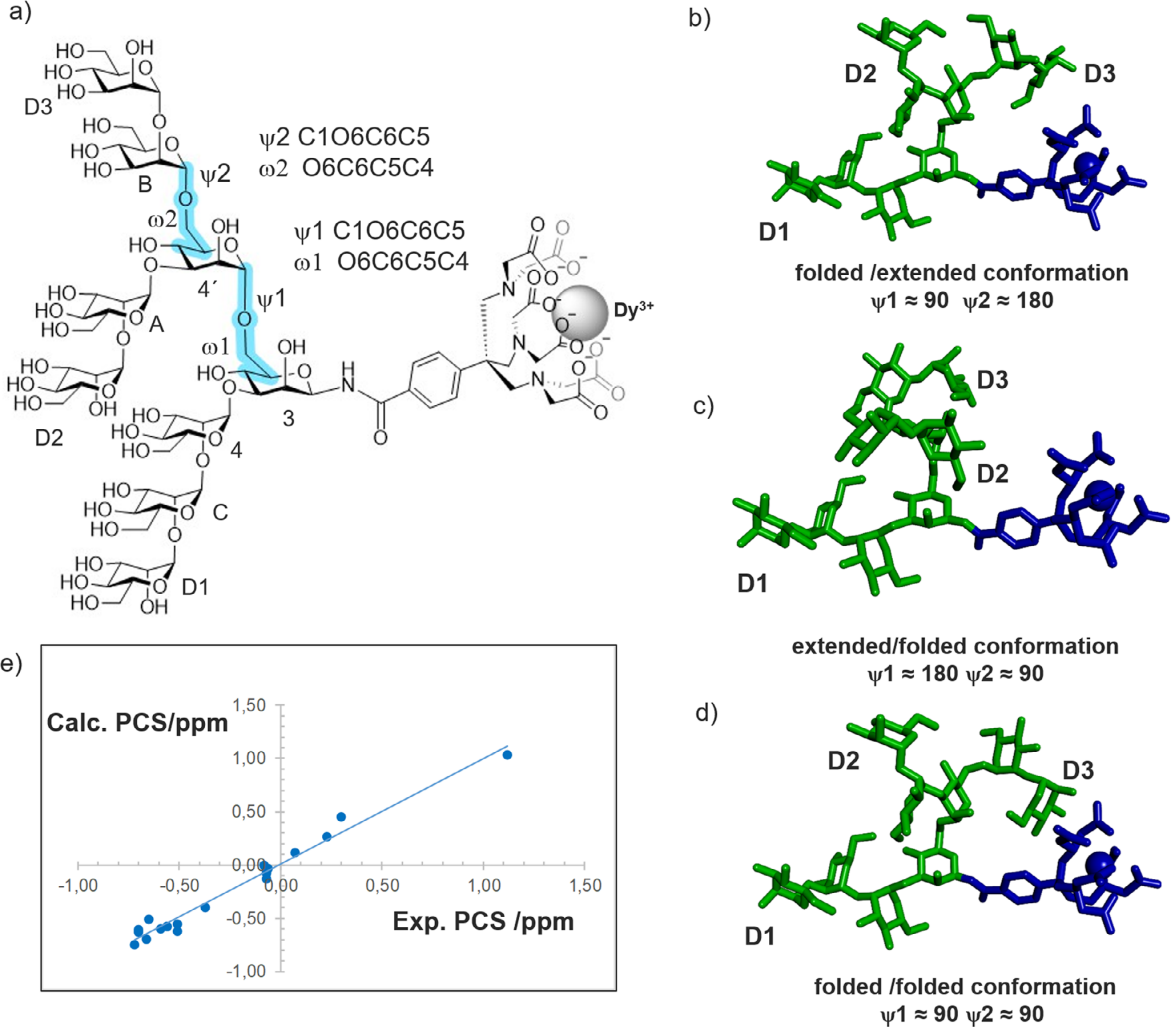
(a) LBT(Dy^3+^)‐Man_9_ structure with the definitions of the key angles that determine the conformation around 1‐6 glycosidic linkages. (b–d) Representative structures of the three families of conformations that provide the best fit to the experimental PCSs. ω1 and ω2 adopts values around 60º in all conformations; ψ1 and ψ2 values are shown in each panel. (e) Correlation between experimental and back‐calculated PCSs from the structural ensemble that includes conformations (b–d).

The 1–6 glycosidic linkages are usually the most flexible linkages in glycans, since in addition to φ (O5C1O6C6) and ψ (C1O6C6C5) torsional angles, they are modulated by ω (O6C6C5C4) torsion (Figure 4) [32].

Small oligomannosides, adopts mainly two conformations: *gauche‐gauche* (ω = 60°) and *gauche‐trans* (ω = 180°) [33], therefore these conformations were considered in LBT(Dy^3+^)‐Man_9_ MSpin calculations. However, the best correlation between experimental and calculated PCSs was obtained when considering just the *gauche–gauche* conformation with ω_1_ and ω_2_ angles around 60º. These results indicate that in Man_9_ structures, the flexibility around ω torsion is restricted, consistent with previous observations [34, 35]. It is also important to examine the behavior of the ψ angle of 1–6 linkages, since this torsion determines whether the glycan adopts extended (ψ = 180°) or folded (ψ  =  90°) conformations. For the ψ_1_ angle, the values that best fit the experimental data in the MSpin calculations are around 90º, indicating that the folded conformer is the most populated, accounting for approximately 70 % of the conformational ensemble. The ψ_2_ angle also adopts values characteristic of a folded conformation, although to lesser extent (population of approximately 40 %). The presence of folded conformations for Man_9_ oligomannosides has also been proposed by Yamaguchi et al. [14].

The best agreement between experimental and back calculated PCSs (associated MSpin quality factor Q_F_ = 0.13) was obtained combining three families of conformations: folded/extended (panel b, Figure 4), extended/folded (panel c, Figure 4), and folded/folded (panel d, Figure 4) with relative populations of 61%, 28% and 11%, respectively. The correlation between experimental and calculated PCSs is shown in Figure 4 panel e. The population weights were computed using the “Fit Populations” tool implemented in the MSpin software. The coordinates of a representative structure from each conformer family were considered in MSpin calculations. These conformers differ mainly in the 1–6 linkages, which, as noted above, can adopt either folded or extended conformations. In all of these structures, the (1–3) and (1–2) linkages display φ and ψ values corresponding to the expected global energy minima for these linkages (see Figure S36 in Section 8 of the Supporting Information). The presence of both extended and folded conformations may allow Man9 to adapt more effectively to the 2G12 binding site.

### 2.3 LBT(Dy^3+^)‐Man_9_/2G12 Binding Studies

**FIGURE 5:**
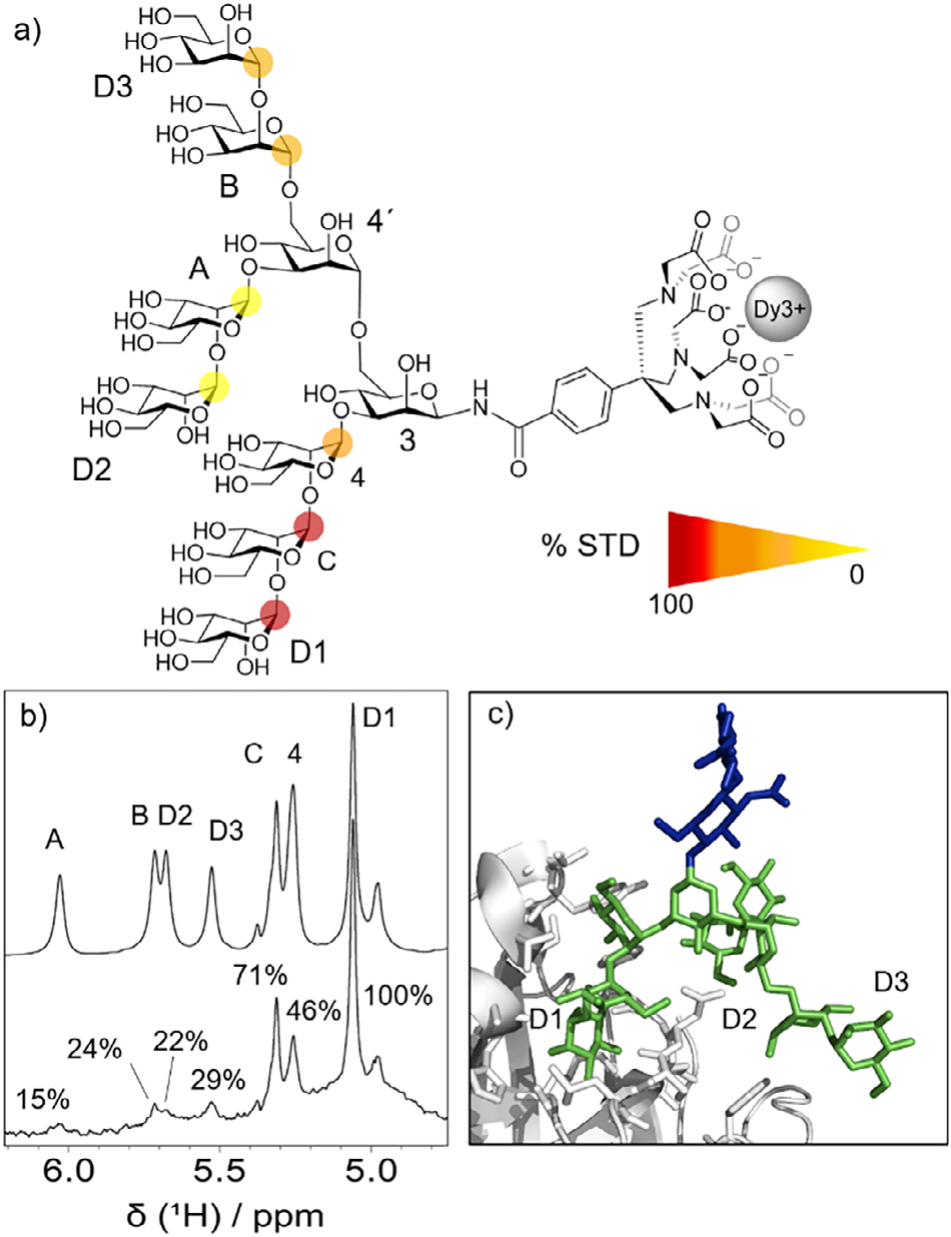
(a) Binding epitope for LBT(Dy^3+^)‐Man_9_ extracted from the ^1^H STD NMR spectrum of (Dy^3+^) in the presence of 2G12 antibody. Relative STD values were calculated using H‐1 of the terminal mannose of the D1 arm as a reference set at 100 %. (b) 600 MHz ^1^H STD NMR off‐resonance (top) and on‐resonance (bottom) spectra of LBT‐Man_9_ **11** loaded with dysprosium in the presence of 2G12. The 4′ H1 signal is weak under paramagnetic conditions due to paramagnetic relaxation enhancement induced by the dysprosium ion and it is not observed in the presence of the protein due to additional line broadening upon binding. Full spectra are shown in Figure S34 in Section 5 of the Supporting Information. (c) X‐Ray structure of Man_9_ in complex with 2G12 (PDB code: 6N2X). The terminal mannoses of D1, D2, and D3 arms are labeled.

After the conformational analysis, LBT‐Man_9_ **11** was used to investigate arm selectivity in the recognition of the 2G12 antibody. A ^1^H STD NMR experiment was performed on a sample of LBT‐Man_9_ **11** loaded with dysprosium in the presence of 2G12 antibody, revealing intense STD signals for the Man_9_ protons (Figure 5). Under these conditions, the signals of arms D1, D2, and D3 could be clearly distinguished, allowing the STD effects of each arm to be quantified. The saturation received by each proton was normalized to the highest STD value, corresponding to the anomeric proton of the terminal mannose in the D1 arm. Strong STD responses were also observed for the mannoses C and 4, both belonging to the D1 arm. In contrast, the D2 and D3 arms showed substantially weaker STD effects, indicating a clear preference for the recognition of the D1 arm.

As described in the introduction, different binding modes have been proposed for the interaction of 2G12 with oligomannosides that differ in the selection of the D1 or D3 arm for the main interaction with the antibody. Our data support the binding mode where the D1 arm is responsible for most of the contacts with 2G12, as described in X‐ray structure (PDB code: 6N2X) [4] and shown in Figure 5c, where the D1 arm is located in the primary binding site, completely surrounded by 2G12 residues. Accordingly, our data indicate that the D1 branch plays a key role in the interaction with 2G12 and should therefore be preserved in immunogen design. The D2 and D3 branches also contribute to binding, albeit with weaker STD effects, suggesting that preservation of the complete structure may enhance immunogenicity. This information is crucial for the design of immunogens to elicit 2G12‐like antibodies as components of vaccines, particularly as recent studies have identified oligomannose glycoepitopes in other viruses such as SARS‐CoV‐2 and certain H3N2 influenza viruses [3].

## 3 Conclusions

The recognition of Man_5_ and Man_9_ oligomannosides present in gp120 by the 2G12 antibody was investigated using ^1^H STD NMR experiments. Our results show that 2G12 can recognize Man_5_ A, a biosynthetic intermediate in the Man_9_ processing pathway, but exhibits a clear preference for Man_9_ structures.

The incorporation of an LBT allowed clear differentiation of the terminal arms in the LBT(Dy^3^^+^)‐Man_9_ complex, overcoming the challenge posed by the similar chemical environments of the D1, D2, and D3 arms, which cannot be individually resolved using standard NMR methods. This strategy enabled detailed conformational analysis in solution by using the experimentally determined pseudo‐contact shift values.

Moreover, the combined application of paramagnetic NMR and STD NMR experiments unambiguously demonstrates that the D1 arm is predominantly recognized by 2G12 in solution, however D2 and D3 branches also contribute to binding. This insight provides a critical basis for the rational design of immunogens aimed at eliciting 2G12‐like antibodies for vaccine development.

## Conflicts of Interest

No. The authors declare no conflicts of interest.

## Supporting information




**Supporting File**: advs74914‐sup‐0001‐SuppMat.pdf.

## Data Availability

The data that support the findings of this study are available from the corresponding author upon reasonable request.
