# Supplementary material for: Structural Insights Into Man9 Recognition by the HIV Antibody 2G12 Revealed by Paramagnetic NMR
Source: Adv Sci (Weinh). 2026 Apr 22;13(31):e00025. doi: 10.1002/advs.202600025 (PMC13588039; doi:10.1002/advs.202600025)
Supplement: Supplementary file 1 [file ADVS-13-e00025-s001.pdf]

Supporting Information

Structural insights into Man_9 recognition by the HIV antibody 2G12 revealed by paramagnetic NMR

Adrián Silva-Díaz,+ [a] Paola Oquist-Phillips,+ [b] Noelia de la Cruz,[a] Inés Lera-Lasso,[b] Laura Castañar,[b] Javier Rojo,*[a] Javier Ramos-Soriano,*[a] Ángeles Canales*[b]

[a] Glycosystems Laboratory, Instituto de Investigaciones Químicas (IIQ), CSIC - Universidad de Sevilla, Av. Américo Vespucio 49, Seville 41092, Spain.

[b] Department of Organic Chemistry, Faculty of Chemistry, Universidad Complutense de Madrid, Avd. Complutense s/n, Madrid 28040, Spain.

+ *AS-D and PO-P contributed equally to this paper.*

* E-mail: javier.rojo@iiq.csic.es, ma.canales@quim.ucm.es and fj.ramos@iiq.csic.es

Table of contents

1. General S2
2. Synthesis and characterization........ S2
3. NMR spectra........ S15
4. Man_5/Man_9 interaction and competition studies........ S27
5. LBT–Man_9 interaction studies........ S33
6. Man_9-LBT pseudo contact shifts measurement........ S34
7. Man_9LBT-La^{3+} and Man_9LBT-Dy^{3+} NMR assignment at 298 K........ S36
8. Molecular Dynamics Simulations........ S38
9. φ / ψ / ω angle distributions for Man_9........ S39
10. Conformational analysis using MSpin Software........ S39
11. References........ S40

1. General

Reagents and solvents were purchased as reagent grade and used without further purification. Compound **3**,[1] *S*-tolyl-2,4-di-*O*-benzoyl-α-D-mannopyranoside (**4**),[2] *S*-tolyl-2,3,4,6-tetra-*O*-benzoyl-α-D-mannopyranoside (**7**),[3] 2,3,4,6-tetra-*O*-benzoyl-α-D-mannopyranosyl trichloroacetimidate (**8**),[4] β-D-mannopyranosyl azide **12**,[5] trisaccharide **14**[3] and pentasaccharide **17**[3] were prepared according to previously reported procedures. Column chromatography was carried out on silica gel 60 (0.040–0.063 mm or 0.015–0.04 mm; Merck) or by Sephadex LH20 or G25 (GE Healthcare, Barcelona, Spain) gel filtration. Thin layer chromatography (TLC) analyses were performed on silica gel 60 F_{254} precoated on aluminium plates (Merck), with detection by UV light (λ = 254 nm) and charred with Moustain or anisaldehyde as development reagents. ^{1}H and ^{13}C NMR spectra were obtained from solutions in CD_3OD, $CDCl_3$, or D_2O at 298 K on Bruker DRX400 and Bruker DRX-500 NMR spectrometers with residual solvent peaks as reference. NMR chemical shifts were reported in ppm (*δ*) and coupling constants (*J*) in Hertz (Hz). All the assignments were confirmed by one- and two-dimensional NMR experiments (^{1}H-^{1}H COSY, ^{1}H-^{13}C HSQC and ^{1}H-^{13}C HMBC). Coupled 2D ^{1}H-^{13}C HSQC NMR experiments were used to verify the stereochemistries of newly created linkage which were unambiguously confirmed by the magnitude of the $^{1}J_{C,H}$ coupling constant.[6] Electrospray mass spectra (ESI MS) were registered at the Mass Spectrometry Service of the Institute for Chemical Research (IIQ, CSIC-US) using an Elute UHPLC system coupled to a Bruker Amazon SL spectrometer instrument. High resolution mass spectra (HR MS) were carried out at Mass Spectrometry Service of CITIUS (University of Seville) using a Thermo Scientific Orbitrap Elite.

2. Synthesis and characterization

2-[2-(2-azidoethoxy)ethoxy]ethyl*O*-2,3,4,6-tetra-*O*-benzoyl-α-D-mannopyranosyl-(1→3)-2-*O*-benzoyl-4,6-*O*-benzylidene-α-D-mannopyranoside (5).

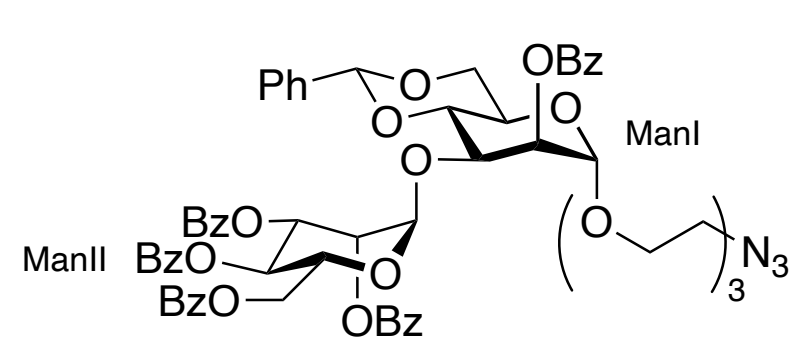

A mixture containing monosaccharide donor **4** (342 mg, 0.49 mmol), acceptor **3** (172 mg, 0.32 mmol) and 4Å MS in anhydrous DCM (8 mL) was stirred at -10 ºC for 15 min. Then, NIS (115 mg, 0.49 mmol) and TfOH (8.6 µL, 0.10 mmol) were added and the resulting mixture was stirred at -10 ºC for 10 min. The reaction mixture was filtered through Celite and the filtrate was washed successively with sat. $Na_2S_2O_3$ aq. soln. and sat. $NaHCO_3$ aq. soln. The organic layer was dried over anh. $MgSO_4$, concentrated and purified by silica gel column chromatography (acetone/toluene, 1:15) to give disaccharide **5** (271 mg, 75%) as a white amorphous solid. ^{1}H NMR (400 MHz, $CDCl_3$) *δ*: 8.31 (m, 2H, H_{Ar}), 8.18 (m, 2H, H_{Ar}), 8.03 (m, 2H, H_{Ar}), 7.92 (m, 2H, H_{Ar}), 7.82 (m, 2H, H_{Ar}), 7.68 (m, 1H, H_{Ar}), 7.65 – 7.49 (m, 7H), 7.48 – 7.23 (m, 12H), 6.17 (t, $^{3}J_{H4\text{-}H3}$ = $^{3}J_{H4\text{-}H5}$ = 10.0, 1H, H-4_{II}), 5.85 (dd, $^{3}J_{H2\text{-}H3}$ = 3.3, $^{3}J_{H2\text{-}H1}$ = 1.9, 1H, H-2_{II}), 5.83 – 5.77 (m, 2H, H-

3$_{II}$, H$_{acetal}$), 5.73 (dd, $^{3}J_{H2\text{-}H3}$ = 3.7, $^{3}J_{H2\text{-}H1}$ = 1.5, 1H, H-2$_{I}$), 5.55 (d, $^{3}J_{H1\text{-}H2}$ = 1.9, 1H, H-1$_{II}$), 5.13 (d, $^{3}J_{H1\text{-}H2}$ = 1.5, 1H, H-1$_{I}$), 4.79 (dd, $^{2}J_{H6a\text{-}H6b}$ = 12.3, $^{3}J_{H6a\text{-}H5}$ = 2.4, 1H, H-6a$_{II}$), 4.69 – 4.58 (m, 2H, H-3$_{I}$, H-5$_{II}$), 4.48 (dd, $^{2}J_{H6b\text{-}H6a}$ = 12.4, $^{3}J_{H6b\text{-}H5}$ = 3.5, 1H, H-6b$_{II}$), 4.40 (dd, $^{2}J_{H6a\text{-}H6b}$ = 10.1, $^{3}J_{H6a\text{-}H5}$ = 4.7, 1H, H-6a$_{I}$), 4.33 (t, $^{3}J_{H4\text{-}H3}$ = $^{3}J_{H4\text{-}H5}$ = 9.7, 1H, H-4$_{I}$), 4.11 (td, $^{3}J_{H5\text{-}H4}$ = 9.8, $^{3}J_{H5\text{-}H6}$ = 4.7, 1H, H-5$_{I}$), 3.97 (t, $^{2}J_{H6a\text{-}H6b}$ = 10.2, 1H, H-6b$_{I}$), 3.90 (m, 1H, C*H*HO), 3.79 – 3.65 (m, 9H, CH_2O, CH_2C$H_2$$N_3$), 3.35 (t, $^{3}J_{H\text{-}H}$ = 5.0, 2H, C$H_2$$N_3$); ^{13}C NMR (101 MHz, $CDCl_3$) δ: 166.3 (CO), 166.2 (CO), 165.3 (CO), 165.2 (CO), 165.0 (CO), 137.1 (C$_{Ar}$), 133.6 (C$_{Ar}$), 133.3 (C$_{Ar}$), 133.1 (C$_{Ar}$), 133.0 (C$_{Ar}$), 130.1 (C$_{Ar}$), 130.1 (C$_{Ar}$), 129.9 (C$_{Ar}$), 129.8 (C$_{Ar}$), 129.7 (C$_{Ar}$), 129.6 (C$_{Ar}$), 129.4 (C$_{Ar}$), 129.3 (C$_{Ar}$), 129.1 (C$_{Ar}$), 128.8 (C$_{Ar}$), 128.6 (C$_{Ar}$), 128.5 (C$_{Ar}$), 128.4 (C$_{Ar}$), 128.3 (C$_{Ar}$), 128.2 (C$_{Ar}$), 126.0 (C$_{Ar}$), 101.4 (C$_{acetal}$), 99.1 (C-1$_{II}$), 98.7 (C-1$_{I}$), 78.9 (C-4$_{I}$), 73.3 (C-3$_{I}$), 72.2 (C-2$_{I}$), 70.9, 70.7, 70.2 – 70.0 (CH_2O, C-2$_{II}$, C-3$_{II}$), 69.4 (C-5$_{II}$), 68.7 (C-6$_{I}$), 67.1 (CH_2O), 66.6 (C-4$_{II}$), 63.6 (C-5$_{I}$), 62.6 (C-6$_{II}$), 50.6 (CH_2N_3); ESI-MS: *m/z* calcd. for $C_{60}H_{57}N_3O_{18}$: 1107.4, found: 1130.3 $[M+Na]^+$; ESI-HRMS: *m/z* calcd. for $C_{60}H_{57}N_3O_{18}Na$ $[M+Na]^+$: 1130.3529, found: 1130.3525.

2-[2-(2-azidoethoxy)ethoxy]ethyl*O*-2,3,4,6-tetra-*O*-benzoyl-α-D-mannopyranosyl-(1→3)-2-*O*-benzoyl-α-D-mannopyranoside (6).

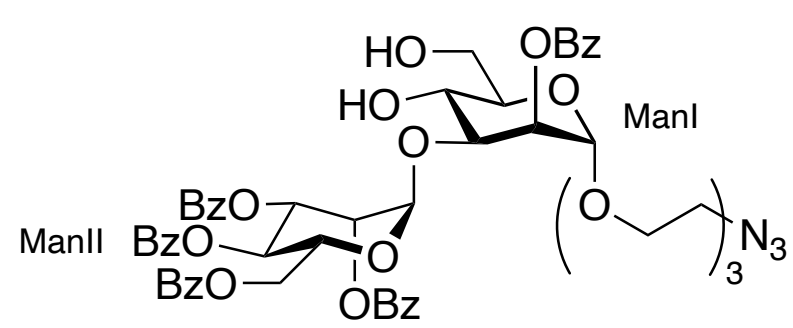

p-Toluenesulfonic acid monohydrate (113 mg, 0.58 mmol) was added to a stirred solution of disaccharide **5** (430 mg, 0.39 mmol) in ACN (20 mL). After 24 h of stirring at r.t., the reaction mixture was quenched with Et_3N (500 µL) and concentrated under vacuum. The residue was purified by silica gel column chromatography (acetone/toluene, 1:4) to give disaccharide **6** (339 mg, 86%) as a white amorphous solid. ^{1}H NMR (400 MHz, $CDCl_3$) δ: 8.18 (m, 2H, H$_{Ar}$), 8.10 (m, 2H, H$_{Ar}$), 7.96 (m, 2H, H$_{Ar}$), 7.80 (m, 2H, H$_{Ar}$), 7.68 (m, 2H, H$_{Ar}$), 7.60 – 7.36 (m, 9H, H$_{Ar}$), 7.33 – 7.22 (m, 6H), 6.10 (t, $^{3}J_{H4\text{-}H3}$ = $^{3}J_{H4\text{-}H5}$ = 10.0, 1H, H-4$_{II}$), 5.78 (dd, $^{3}J_{H2\text{-}H3}$ = 3.2, $^{3}J_{H2\text{-}H1}$ = 1.9 Hz, 1H, H-2$_{II}$), 5.72 (dd, $^{3}J_{H3\text{-}H4}$ = 10.1, $^{3}J_{H3\text{-}H2}$ = 3.2, 1H, H-3$_{II}$), 5.57 (dd, $^{3}J_{H2\text{-}H3}$ = 3.2, $^{3}J_{H2\text{-}H1}$ = 1.7, 1H, H-2$_{I}$), 5.46 (d, $^{3}J_{H1\text{-}H2}$ = 2.0, 1H, H-1$_{II}$), 5.06 (d, $^{3}J_{H1\text{-}H2}$ = 1.7, 1H, H-1$_{I}$), 4.61 (dd, $^{2}J_{H6a\text{-}H6b}$ = 12.5, $^{3}J_{H6a\text{-}H5}$ = 2.5, 1H, H-6a$_{II}$), 4.41 – 4.26 (m, 4H, H-3$_{I}$, H-4$_{I,}$ H-5$_{II}$, H-6b$_{II}$), 3.97 – 3.94 (m, 2H, 2H-6$_{I}$), 3.92 – 3.82 (m, 2H, H-5$_{I}$, C*H*HO), 3.73 – 3.61 (m, 9H, CH_2O, CH_2C$H_2$$N_3$), 3.37 (m, 2H, C$H_2$$N_3$); ^{13}C NMR (101 MHz, $CDCl_3$) δ: 166.1 (CO), 166.1 (CO), 165.7 (CO), 165.4 (CO), 165.1 (CO), 133.5 (C$_{Ar}$), 133.4 (C$_{Ar}$), 133.3 (C$_{Ar}$), 133.2 (C$_{Ar}$), 133.0 (C$_{Ar}$), 130.0 – 129.6 (C$_{Ar}$), 129.2 (C$_{Ar}$), 129.0 (C$_{Ar}$), 128.9 (C$_{Ar}$), 128.7 – 128.3 (C$_{Ar}$), 99.9 (C-1$_{II}$), 97.7 (C-1$_{I}$), 79.2 (C-4$_{I}$), 77.4, 72.3 – 72.3, 70.8 – 70.7, 70.5, 70.2 – 70.1 (CH_2O, C-2$_{I}$, C-2$_{II}$, C-3$_{I}$, C-3$_{II,}$ C-5$_{I}$), 69.5 (C-5$_{II}$), 67.1 (CH_2O), 66.2 (C-4$_{II}$), 62.4 (C-6$_{II}$), 62.2 (C-6$_{I}$), 50.6 (CH_2N_3); ESI-MS: *m/z* calcd. for $C_{53}H_{53}N_3O_{18}$: 1019.3, found: 1042.2 $[M+Na]^+$; ESI-HRMS: *m/z* calcd. for $C_{53}H_{53}N_3O_{18}Na$ $[M+Na]^+$: 1042.3216, found: 1042.3209.

***S*-Tolyl2,3,4,6-tetra-*O*-benzoyl-α-D-mannopyranosyl-(1→3)-[2,3,4,6-tetra-*O*-benzoyl-α-D mannopyranosyl-(1→6)]-2,4-di-*O*-benzoyl-α-D-mannopyranoside (9).**

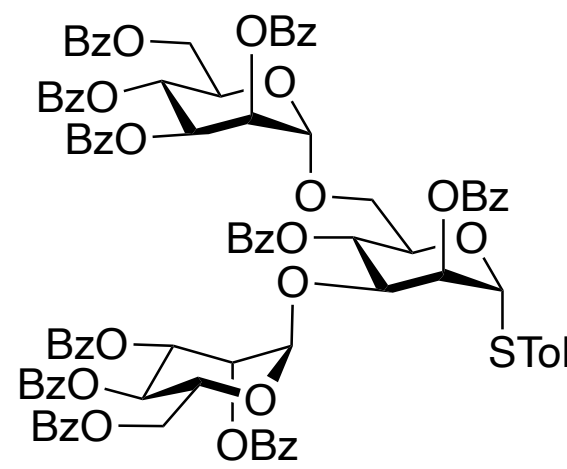

A mixture containing diol acceptor **7** (168 mg, 0.34 mmol), 2,3,4,6-tetra-*O*-benzoyl-α-D-mannopyranosyl trichloroacetimidate (**8**) (752 mg, 1.02 mmol) and 4 Å MS in anhydrous DCM (5 mL) was stirred at -10 ºC for 15 min. Then, TMSOTf (20 µL, 0.10 mmol) was added, and the reaction mixture was stirred at -10 ºC for 15 min. The reaction was neutralized with Et_3N (160 µL), filtered through Celite and washed several times with DCM. After removal of the solvent, the crude was purified by silica gel column chromatography (acetone/toluene, 1:40) to afford trisaccharide **9** (449 mg, 80%) as a white amorphous solid. ^{1}H NMR (400 MHz, $CDCl_3$) *δ*: 8.35 (dd, $^3J_{HAr-HAr}$ = 7.5, $^3J_{HAr-HAr}$ = 2.1, 2H, H_{Ar}), 8.22 – 8.15 (m, 4H, H_{Ar}), 8.09 – 8.02 (m, 6H, H_{Ar}), 7.92 – 7.87 (m, 4H, H_{Ar}), 7.82 (d, $^3J_{HAr-HAr}$ = 7.8, 2H, H_{Ar}), 7.75 (d, $^3J_{HAr-HAr}$ = 7.7, 2H, H_{Ar}), 7.62 – 7.30 (m, 30H, H_{Ar}), 7.24 – 7.18 (m, 4H), 6.18 (t, $^3J_{H4-H3}$ = $^3J_{H4-H5}$ = 10.1, 1H, H-4), 6.15 – 6.02 (m, 4H, H-2, 3xH-3), 5.88 (d, $^3J_{H2-H1}$ = 2.0, 1H, H-2), 5.84 – 5.77 (m, 2H, H-1, H-2), 5.49 – 5.42 (m, 2H, H-1), 5.18 (s, 1H, H-1), 4.92 (m, 1H, H-5), 4.67 – 4.28 (m, 8H, 4xH-6), 4.27 (m, 1H, H-6), 3.85 (m, 1H, H-6), 2.21 (s, 3H, CH_3Ph); ^{13}C NMR (101 MHz, $CDCl_3$) *δ*: 166.3 (CO), 166.3 (CO), 166.2 (CO), 165.7 (CO), 165.6 (CO), 165.4 (CO), 165.3 (CO), 164.8 (CO), 138.7 (C_{Ar}), 133.8 (C_{Ar}), 133.6 (C_{Ar}), 133.5 (C_{Ar}), 133.4 (C_{Ar}), 133.4 (C_{Ar}), 133.2-133.1 (C_{Ar}), 132.9 (C_{Ar}), 130.3 (C_{Ar}), 130.2 (C_{Ar}), 130.1 (C_{Ar}), 130.0 (C_{Ar}), 130.0 (C_{Ar}), 129.8 (C_{Ar}), 129.7 (C_{Ar}), 129.4 (C_{Ar}), 129.3 (C_{Ar}), 129.3 (C_{Ar}), 129.2 (C_{Ar}), 129.1 (C_{Ar}), 129.1 (C_{Ar}), 129.0 (C_{Ar}), 128.7 (C_{Ar}), 128.6 (C_{Ar}) 128.6 (C_{Ar}), 128.5 (C_{Ar}), 128.4 (C_{Ar}), 128.3 (C_{Ar}), 99.7 (C-1), 98.1 (C-1), 86.7 (C-1), 73.4, 70.8, 70.5, 70.3 (CH_2O), 69.9, 69.5, 69.0, 68.8, 67.3, 66.7, 66.6, 62.8, 62.7, 21.2 (*C*H_3Ph); ESI-MS: *m/z* calcd. for $C_{95}H_{78}O_{25}S$: 1650.4, found: 1673.0 $[M+Na]^+$; ESI-HRMS: *m/z* calcd. for $C_{95}H_{78}O_{25}SNa$ $[M+Na]^+$: 1673.4177, found: 1673.4457.

2-[2-(2-azidoethoxy)ethoxy]ethyl*O*-2,3,4,6-tetra-*O*-benzoyl-α-D-mannopyranosyl-(1→3)-{2,3,4,6-tetra-*O*-benzoyl-α-D-mannopyranosyl-(1→3)-[2,3,4,6-tetra-*O*-benzoyl-α-D-mannopyranosyl)-(1→6)]-2-*O*-benzoyl-α-D-mannopyranosyl)-(1→6)}-2-*O*-benzoyl-α-D-mannopyranoside (10).

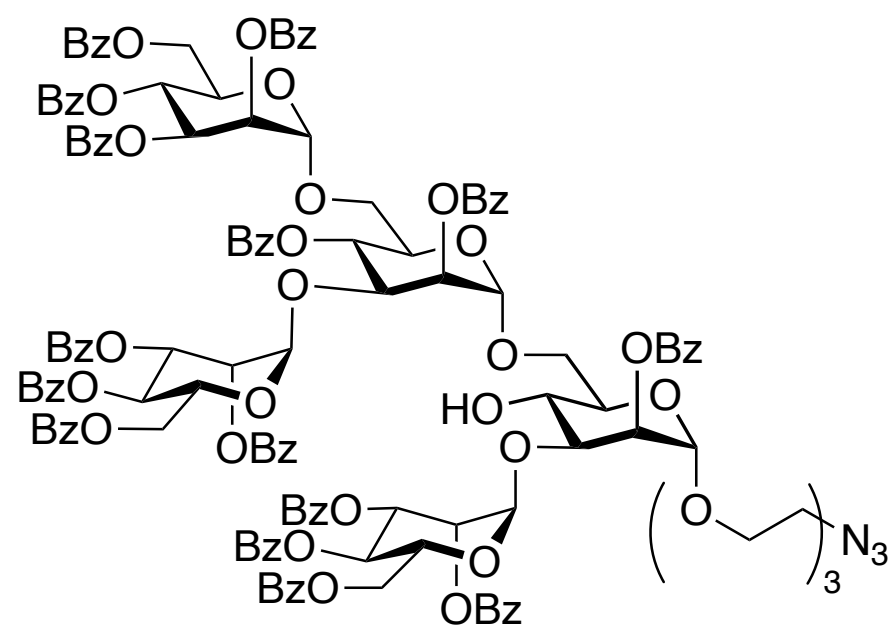

A mixture containing trisaccharide donor **9** (300 mg, 0.18 mmol), acceptor **6** (143 mg, 0.14 mmol) and 4Å MS in anhydrous DCM (10 mL) was stirred at -10 ºC for 15 min. Then, NIS (43 mg, 0.18 mmol) and TfOH (5 µL, 0.056 mmol) were added and the resulting mixture was stirred at -10 ºC for 15 min. The reaction mixture was filtered through Celite and the filtrate was washed successively with sat. $Na_2S_2O_3$ aq. soln. and sat. $NaHCO_3$ aq. soln. The organic layer was dried over anh. $MgSO_4$, concentrated and purified by silica gel column chromatography (acetone/toluene, 1:12) to obtain pentasaccharide **10** (344 mg, 97%) as a white amorphous solid. ^{1}H NMR (400 MHz, $CDCl_3$) *δ*: 8.27 (d, $^3J_{HAr-HAr}$ = 7.1, 2H, H_{Ar}), 8.20 – 8.03 (m, 10H, H_{Ar}), 8.01 – 7.96 (m, 4H, H_{Ar}),), 7.88 – 7.81 (m, 6H, H_{Ar}),), 7.73 – 7.66 (m, 8H, H_{Ar}), 7.64 – 7.49 (m, 10H, H_{Ar}), 7.48 – 7.16 (m, 33H, H_{Ar}), 7.14 (t, $^3J_{HAr-HAr}$ = 7.6, 2H, H_{Ar}), 6.14 (t, $^3J_{HAr-HAr}$ = 10.0, 1H, H_{Ar}), 6.10 – 6.01 (m, 3H), 5.88 (t, $^3J_{H-H}$ = 10.0, 1H), 5.83 (dd, $^3J_{H-H}$ = 3.5, $^3J_{H-H}$ = 1.6, 1H), 5.75 (dd, $^3J_{H-H}$ = 3.2, $^3J_{H-H}$ = 1.8, 1H), 5.73 – 5.67 (m, 3H), 5.63 (m, 1H), 5.43 (d, $^3J_{H1-H2}$ = 1.5, 1H, H-1), 5.39 (dd, *J* = 3.2, *J* = 1.9, 1H), 5.36 (m, 2H, 2xH-1), 5.19 (d, $^3J_{H1-H2}$ = 1.8, 1H, H-1), 5.12 (d, $^3J_{H1-H2}$ = 1.6, 1H, H-1), 4.72 (dd, $^3J_{H-H}$ = 9.7, $^3J_{H-H}$ = 3.5, 1H), 4.66 – 4.11 (m, 17H), 3.95 (m, 1H), 3.82 (dd, $^3J_{H-H}$ = 10.9, $^3J_{H-H}$ = 2.0, 1H), 3.78 – 3.51 (m, 9H), 3.29 (m, 2H, CH_2N_3); ^{13}C NMR (101 MHz, $CDCl_3$) *δ*: 166.3 (CO), 166.2 (3xCO), 166.1 (2xCO), 166.0 (CO), 165.6 (CO), 165.4 (CO), 165.2 (3xCO), 165.0 (CO), 164.8 (CO), 164.8 (CO), 133.7 (C_{Ar}), 133.5 (C_{Ar}), 133.4 (C_{Ar}), 133.3 (C_{Ar}), 133.2 (C_{Ar}), 133.0 (C_{Ar}), 130.3-129.5 (C_{Ar}), 129.4 (C_{Ar}), 129.3 (C_{Ar}), 129.3 (C_{Ar}), 129.2 (C_{Ar}), 129.1 (C_{Ar}), 129.0 (C_{Ar}), 128.8-128.0 (C_{Ar}), 99.9 (C-1), 99.9 (C-1), 97.5 (C-1), 97.5 (C-1), 96.7 (C-1), 79.0, under $CDCl_3$ (2xC), 72.4, 72.1, 70.8, 70.6, 70.4, 70.4, 70.3, 70.2, 70.1, 69.8, 69.7, 69.7, 69.5, 69.0, 68.8, 67.7, 67.0, 66.7, 66.4, 66.3, 62.8, 62.6, 62.3, 50.7 (CH_2N_3); ESI-MS: *m/z* calcd. for $C_{143}H_{123}N_3O_{43}$: 2546.7, found: 2569.5 $[M+Na]^+$ and 1296.2 $[M+2Na]^{2+}$; ESI-HRMS *m/z* calcd. for $C_{143}H_{123}N_3O_{43}Na$ $[M+Na]^+$: 2568.7423, found: 2568.7351.

2-[2-(2-azidoethoxy)ethoxy]ethyl *O*-α-D-mannopyranosyl-(1→3)-{α-D-mannopyranosyl-(1→3)-[α-D-mannopyranosyl)-(1→6)]-*O*-α-D-mannopyranosyl)-(1→6)}-*O*-α-D-mannopyranoside (1).

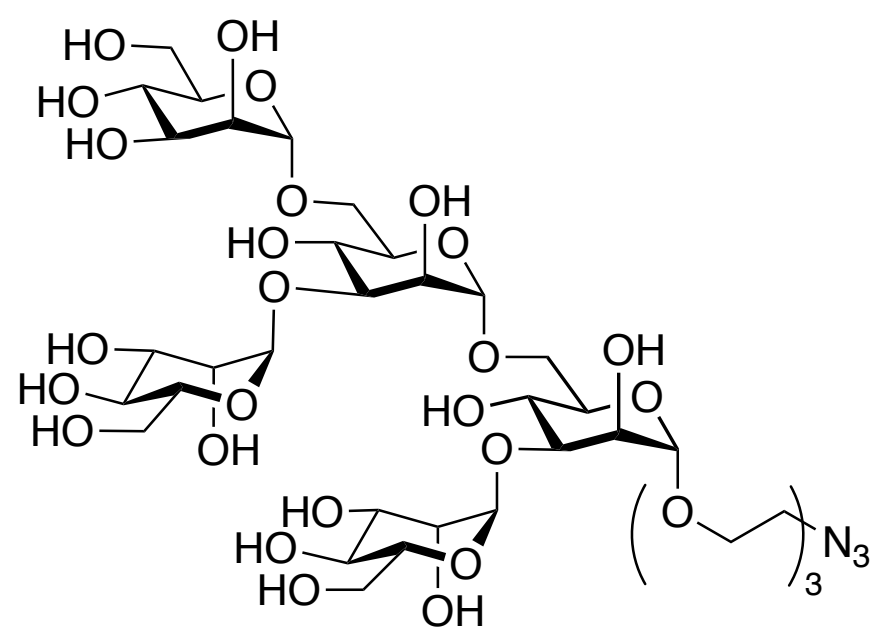

To a solution of pentasaccharide **10** (300 mg, 0,12 mmol) in MeOH/toluene (4:1, 5 mL), NaOMe (19 mg, 0.35 mmol) was added. The reaction mixture was stirred at r.t. for 3 h, then was neutralized with Amberlite IR-120 (H^+) resin, filtered and the solvent was removed under vacuum. The crude was purified by size-exclusion chromatography (Sephadex LH-20, MeOH), to give unprotected pentasaccharide **1** (110 mg, 95%) as a white amorphous solid. ^{1}H NMR (500 MHz, D_2O) *δ*: 5.15 (d, $^3J_{H1\text{-}H2}$ = 1.8, 1H, H-1), 5.13 (d, $^3J_{H1\text{-}H2}$ = 1.8, 1H, H-1), 4.92 (d, $^3J_{H1\text{-}H2}$ = 1.9, 1H, H-1), 4.89 – 4.86 (m, 2H, 2xH-1), 4.17 – 4.13 (m, 2H, 2xH-2), 4.10 – 4.07 (m, 2H, 2xH-2), 4.03 – 3.98 (m, 3H), 3.96 – 3.84 (m, 13H), 3.81 – 3.66 (m, 20H), 3.52 (m, 2H); ^{13}C NMR (126 MHz, D_2O) *δ*: 102.4 (C-1), 102.3 (C-1), 100.2 (C-1), 99.4 (C-1), 99.3 (C-1), 78.7 – 78.5, 73.3 – 73.3, 72.7, 70.9 – 70.9, 70.6, 70.4, 70.1 – 70.0, 69.7 – 69.6, 69.3, 66.7, 65.8 – 65.6, 65.3, 61.0 – 60.9, 50.1 (CH_2N_3); ESI-MS: *m/z* calcd. for $C_{36}H_{63}N_3O_{28}$: 985.4, found: 1008.1 [M+Na]$^+$; ESI-HRMS: *m/z* calcd. for $C_{36}H_{63}N_3O_{28}Na$ [M+Na]$^+$: 1008.3490, found: 1008.3478.

SYNTHESIS OF KEY INTERMEDIATE 13

Stepwise synthesis.

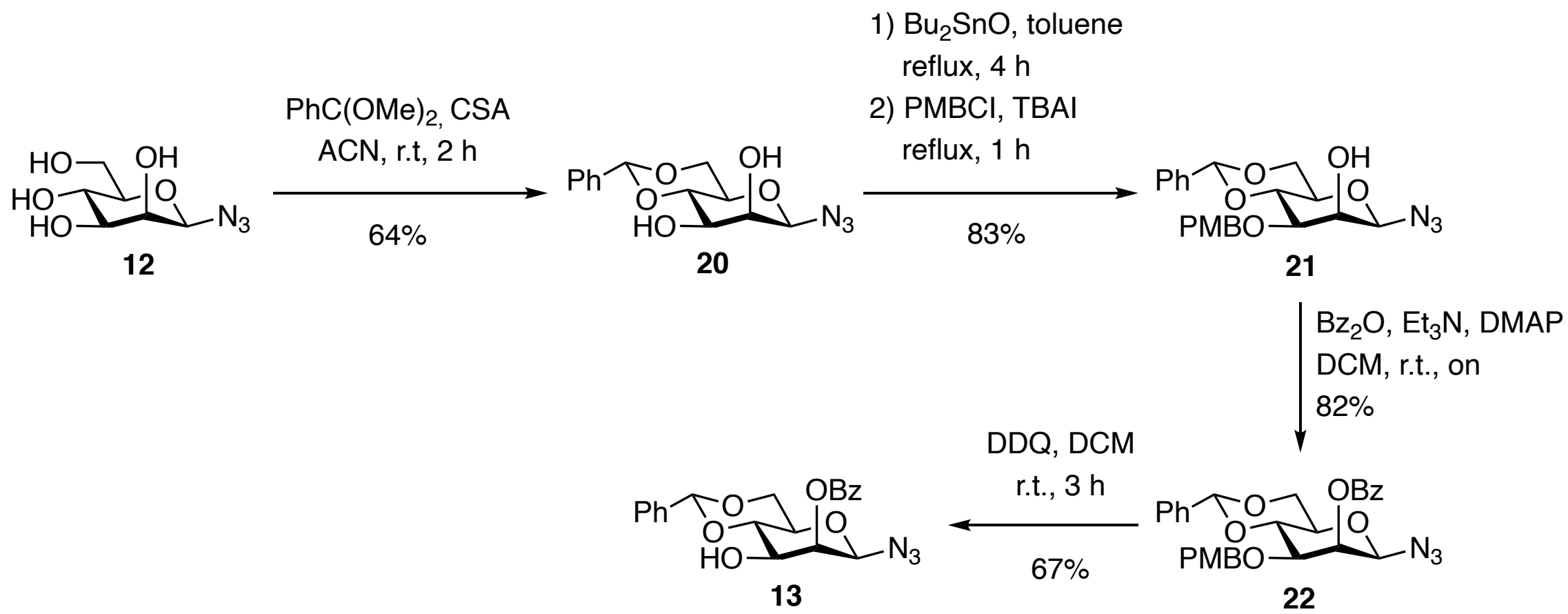

Azido 4,6-*O*-(phenylmethylene)-β-D-mannopyranoside (20)

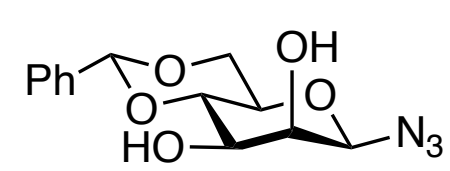

To a solution of unprotected β-D-mannopyranosyl azide **12** (190 mg, 0.93 mmol) in anhydrous ACN (3 mL), CSA (55 mg, 0.23 mmol) and benzaldehyde dimethyl acetal (0.15 mL, 1.02 mmol) were added. The reaction mixture was stirred at r.t. for 2 h and then quenched with Et_3N (0.3 mL). The mixture was concentrated, and the resulting crude was purified by column chromatography on silica gel (EtOAc/*n*-hexane, 1:2) to afford **20** (175 mg, 64%) as a pale-yellow syrup. ^{1}H NMR (400 MHz, $CDCl_3$) δ: 7.49 (m, 2H, $H_{o\text{-}Ph}$), 7.41 – 7.35 (m, 3H, $H_{m\text{-}Ph}$, $H_{p\text{-}Ph}$), 5.57 (s, 1H, H_{acetal}), 4.69 (m, 1H, H-1), 4.37 (dd, $^2J_{H6a\text{-}H6b}$ = 10.5, $^3J_{H6a\text{-}H5}$ = 4.9, 1H, H-6a), 4.05 (m, 1H, H-2), 3.93 (t, $^3J_{H4\text{-}H3}$ = $^3J_{H4\text{-}H5}$ = 9.5, 1H, H-4), 3.87 (t, $^2J_{H6b\text{-}H6a}$ = $^3J_{H6b\text{-}H5}$ = 10.3, 1H, H-6b), 3.81 (m, 1H, H-3), 3.46 (ddd, $^3J_{H5\text{-}H6b}$ = 10.3, $^3J_{H5\text{-}H4}$ = 9.7, $^3J_{H5\text{-}H6a}$ = 5.0, 1H, H-5), 2.82 (d, $^3J_{OH\text{-}H3}$ = 5.0, 1H, OH-3), 2.68 (d, $^3J_{OH\text{-}H2}$ = 2.2, 1H, OH-2); ^{13}C NMR (101 MHz, $CDCl_3$) δ: 137.1 ($C_{ipso\text{-}Ph}$), 129.5 ($C_{p\text{-}Ph}$), 128.5 ($C_{m\text{-}Ph}$), 126.4 ($C_{o\text{-}Ph}$), 102.4 (C_{acetal}), 87.8 (C-1), 78.1 (C-4), 71.4 (C-2), 70.9 (C-3), 69.0 (C-5), 68.4 (C-6); ESI-MS: *m/z* calcd. for $C_{13}H_{15}N_3O_5$: 293.1; found: 316.0 $[M+Na]^+$; ESI-HRMS: *m/z* calcd. for $C_{13}H_{15}N_3O_5Na$ $[M+Na]^+$: 316.0904; found: 316.0902.

Azido 3-*O*-[(4-methoxyphenyl)methyl]-4,6-*O*-(phenylmethylene)-β-D-mannopyranoside (21)

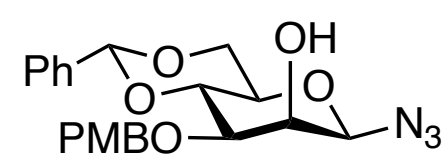

A mixture of compound **20** (175 mg, 0.60 mmol), Bu_2SnO (163 mg, 0.66 mmol) and 4Å MS in anhydrous toluene (9 mL) was refluxed for 4 h. Then, the reaction mixture was cooled down to r.t. and PMBCl (90 μL, 0.63 mmol) and TBAI (243 mg, 0.66 mmol) were added. After 1 h of refluxing, the reaction mixture was filtered and washed with DCM. The organic phase was washed with H_2O (x2) and then dried over anh. $MgSO_4$, filtered and concentrated under vacuum. The crude was purified by silica gel column chromatography (EtOAc/*n*-hexane, 1:4) to afford **21** (204 mg, 83%) as a white amorphous solid. ^{1}H NMR (400 MHz, $CDCl_3$) *δ*: 7.50 (m, 2H, $H_{o\text{-Ph}}$), 7.43 – 7.36 (m, 3H, $H_{m\text{-Ph}}$, $H_{p\text{-Ph}}$), 7.28 (d, $^3J_{Ho\text{-}Hm}$ = 8.7, 2H, $H_{o\text{-PMB}}$), 6.87 (d, $^3J_{Hm\text{-}Ho}$ = 8.7, 2H, $H_{m\text{-PMB}}$), 5.61 (s, 1H, H_{acetal}), 4.73 (AX system, 2H, $CH_{2\ PMB}$), 4.55 (d, $^3J_{H1\text{-}H2}$ = 1.5, 1H, H-1), 4.36 (dd, $^2J_{H6a\text{-}H6b}$ = 10.5, $^3J_{H6a\text{-}H5}$ = 4.9, 1H, H-6a), 4.12 (t, $^3J_{H4\text{-}H3}$ = $^3J_{H4\text{-}H5}$ = 9.5, 1H, H-4), 4.04 (dd, $^3J_{H2\text{-}H3}$ = 3.3, $^3J_{H2\text{-}H1}$ = 1.4, 1H, H-2), 3.90 (t, $^2J_{H6b\text{-}H6a}$ = $^3J_{H6b\text{-}H5}$ = 10.4, 1H, H-6b), 3.81 (s, 3H, $CH_{3,PMB}$), 3.64 (dd, $^3J_{H3\text{-}H4}$ = 9.5, $^3J_{H3\text{-}H2}$ = 3.3, 1H, H-3), 3.45 (ddd, $^3J_{H5\text{-}H6b}$ = 10.2, $^3J_{H5\text{-}H4}$ = 9.7, $^3J_{H5\text{-}H6a}$ = 5.0, 1H, H-5); ^{13}C NMR (101 MHz, $CDCl_3$) *δ*: 159.6 ($C_{ipso\text{-OMe,PMB}}$), 137.4 ($C_{ipso\text{-Ph}}$), 129.8 ($C_{o\text{-PMB}}$), 129.7 ($C_{ipso\text{-CH_2,PMB}}$), 129.2 ($C_{p\text{-Ph}}$), 128.4 ($C_{m\text{-Ph}}$), 126.2 ($C_{o\text{-Ph}}$), 114.1 ($C_{m\text{-PMB}}$), 101.7 (C_{acetal}), 87.5 (C-1), 78.1 (C-4), 76.5 (C-3), 72.7 ($CH_{2,PMB}$), 70.8 (C-2), 69.2 (C-5), 68.4 (C-6), 55.4 ($CH_{3,PMB}$); ESI-MS: *m/z* calcd. for $C_{21}H_{23}N_3O_6$: 413.2; found: 436.2 $[M+Na]^+$; ESI-HRMS: *m/z* calcd. for $C_{21}H_{23}N_3O_6Na$ $[M+Na]^+$: 436.1479; found: 436.1479.

Azido 2-*O*-benzoyl-3-*O*-[(4-methoxyphenyl)methyl]-4,6-*O*-(phenylmethylene)-β-D-mannopyranoside (22)

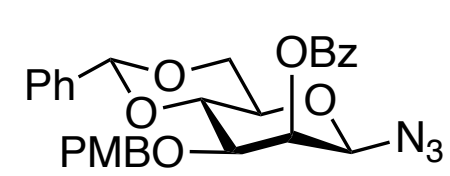

To a solution of **21** (204 mg, 0.49 mmol) in DCM (10 mL), Bz_2O (239 mg, 1.00 mmol), Et_3N (0.21 mL, 1.51 mmol) and a catalytic amount of DMAP were subsequently added, and the reaction mixture was stirred overnight at r.t. The reaction mixture was washed with 1M HCl, H_2O and brine. The organic phase was dried over anh. $MgSO_4$, filtered and concentrated. The crude was purified by silica gel column chromatography (EtOAc/*n*-hexane, 1:6) to give monosaccharide **22** (209 mg, 82%) as a white amorphous solid. ^{1}H NMR (400 MHz, $CDCl_3$) *δ*: 8.17 (m, 2H, $H_{o\text{-Bz}}$), 7.61 (m, 1H, $H_{p\text{-Bz}}$), 7.56 – 7.48 (m, 4H, $H_{m\text{-Bz}}$, $H_{o\text{-Ph}}$), 7.45 – 7.39 (m, 3H, $H_{m\text{-Ph}}$, $H_{p\text{-Ph}}$), 7.29 (d, $^3J_{Ho\text{-}Hm}$ = 8.7, 2H, $H_{o\text{-PMB}}$), 6.84 (d, $^3J_{Hm\text{-}Ho}$ = 8.7, 2H, $H_{m\text{-PMB}}$), 5.82 (dd, $^3J_{H2\text{-}H3}$ = 3.4, $^3J_{H2\text{-}H1}$ = 1.4, 1H, H-2), 5.68 (s, 1H, H_{acetal}), 4.75 (d, $^3J_{H1\text{-}H2}$ = 1.4, 1H, H-1), 4.67 (AX system, 2H, $CH_{2,PMB}$), 4.44 (dd, $^2J_{H6a\text{-}H6b}$ = 10.5, $^3J_{H6a\text{-}H5}$ = 4.9, 1H, H-6a), 4.14 (t, $^3J_{H4\text{-}H3}$ = $^3J_{H4\text{-}H5}$ = 9.5, 1H,

H-4), 3.98 (t, $^{2}J_{H6b-H6a}$ = $^{3}J_{H6b-H5}$ = 10.3, 1H, H-6b), 3.83 (dd, $^{3}J_{H3-H4}$ = 9.8, $^{3}J_{H3-H2}$ = 3.4, 1H, H-3), 3.78 (s, 3H, $CH_{3,PMB}$), 3.57 (ddd, $^{3}J_{H5-H6b}$ = 10.3, $^{3}J_{H5-H4}$ = 9.5, $^{3}J_{H5-H6a}$ = 4.9, 1H, H-5); ^{13}C NMR (101 MHz, $CDCl_3$) *δ*: 165.7 (CO), 159.4 ($C_{ipso\text{-}OMe,PMB}$), 137.3 ($C_{ipso\text{-}Ph}$), 133.5 ($C_{p\text{-}Bz}$), 130.2 ($C_{o\text{-}Bz}$), 129.6 ($C_{ipso\text{-}Bz}$), 129.5 ($C_{o\text{-}PMB}$), 129.5 ($C_{ipso\text{-}CH_2,PMB}$), 129.1 ($C_{p\text{-}Ph}$), 128.6 ($C_{m\text{-}Bz}$), 128.3 ($C_{m\text{-}Ph}$), 126.2 ($C_{o\text{-}Ph}$), 113.9 ($C_{m\text{-}PMB}$), 101.7 (C_{acetal}), 86.5 (C-1), 77.8 (C-4), 75.3 (C-3), 71.6 ($CH_{2,PMB}$), 69.9 (C-2), 69.7 (C-5), 68.4 (C-6), 55.3 ($CH_{3,PMB}$); ESI-MS: *m/z* calcd. for $C_{28}H_{27}N_3O_7$: 517.2; found: 540.3 $[M+Na]^+$; ESI-HRMS: *m/z* calcd. for $C_{28}H_{27}N_3O_7Na$ $[M+Na]^+$: 540.1741; found: 540.1733.

Azido 2-*O*-benzoyl-4,6-*O*-(phenylmethylene)-β-D-mannopyranoside (13)

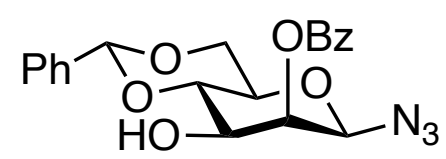

To a solution of mannoside derivative **22** (209 mg, 0.41 mmol) in DCM (12 mL) and H_2O (1 mL), DDQ (299 mg, 1.29 mmol) was added and the reaction mixture was stirred at r.t. for 3 h. Then, the reaction mixture was diluted with DCM and washed with H_2O (x3) and brine. The organic phase was dried over anh. $MgSO_4$, filtered and the solvent was evaporated. The crude was purified by silica gel column chromatography (EtOAc/*n*-hexane, 1:4) to afford compound **13** (108 mg, 67%) as a white amorphous solid. ^{1}H NMR (400 MHz, $CDCl_3$) *δ*: 8.15 (d, $^{3}J_{Ho-Hm}$ = 7.6, 2H, $H_{o\text{-}Bz}$), 7.64 (t, $^{3}J_{Hp-Hm}$ = 7.5, 1H, $H_{p\text{-}Bz}$), 7.55 – 7.48 (m, 4H, $H_{m\text{-}Bz}$, $H_{o\text{-}Ph}$), 7.44 – 7.38 (m, 3H, $H_{m\text{-}Ph}$, $H_{p\text{-}Ph}$), 5.68 (m, 1H, H-2), 5.66 (s, 1H, H_{acetal}), 4.79 (m, 1H, H-1), 4.46 (dd, $^{2}J_{H6a-H6b}$ = 10.6, $^{3}J_{H6a-H5}$ = 5.0, 1H, H-6a), 4.10 (m, 1H, H-3), 4.04 – 3.94 (m, 2H, H-4, H-6b), 3.60 (ddd, ^{3}J = 10.4, ^{3}J = 9.6, $^{3}J_{H5-H6a}$ = 5.0, 1H, H-5), 2.71 (d, $^{3}J_{OH-H3}$ = 3.0, 1H, OH); ^{13}C NMR (101 MHz, $CDCl_3$) *δ*: 166.0 (CO), 136.8 ($C_{ipso\text{-}Ph}$), 133.6 ($C_{p\text{-}Bz}$), 130.1 ($C_{o\text{-}Bz}$), 129.5 ($C_{p\text{-}Ph}$), 129.2 ($C_{ipso\text{-}Bz}$), 128.6 ($C_{m\text{-}Bz}$), 128.4 ($C_{m\text{-}Ph}$), 126.3 ($C_{o\text{-}Ph}$), 102.3 (C_{acetal}), 86.4 (C-1), 78.4 (C-4), 71.9 (C-2), 70.0 (C-3), 69.2 (C-5), 68.3 (C-6); ESI-MS: *m/z* calcd. for $C_{20}H_{19}N_3O_6$: 397.1; found: 420.1 $[M+Na]^+$; ESI-HRMS: *m/z* calcd. for $C_{20}H_{19}N_3O_6Na$ $[M+Na]^+$: 420.1166; found: 420.1160.

Consecutive approach.

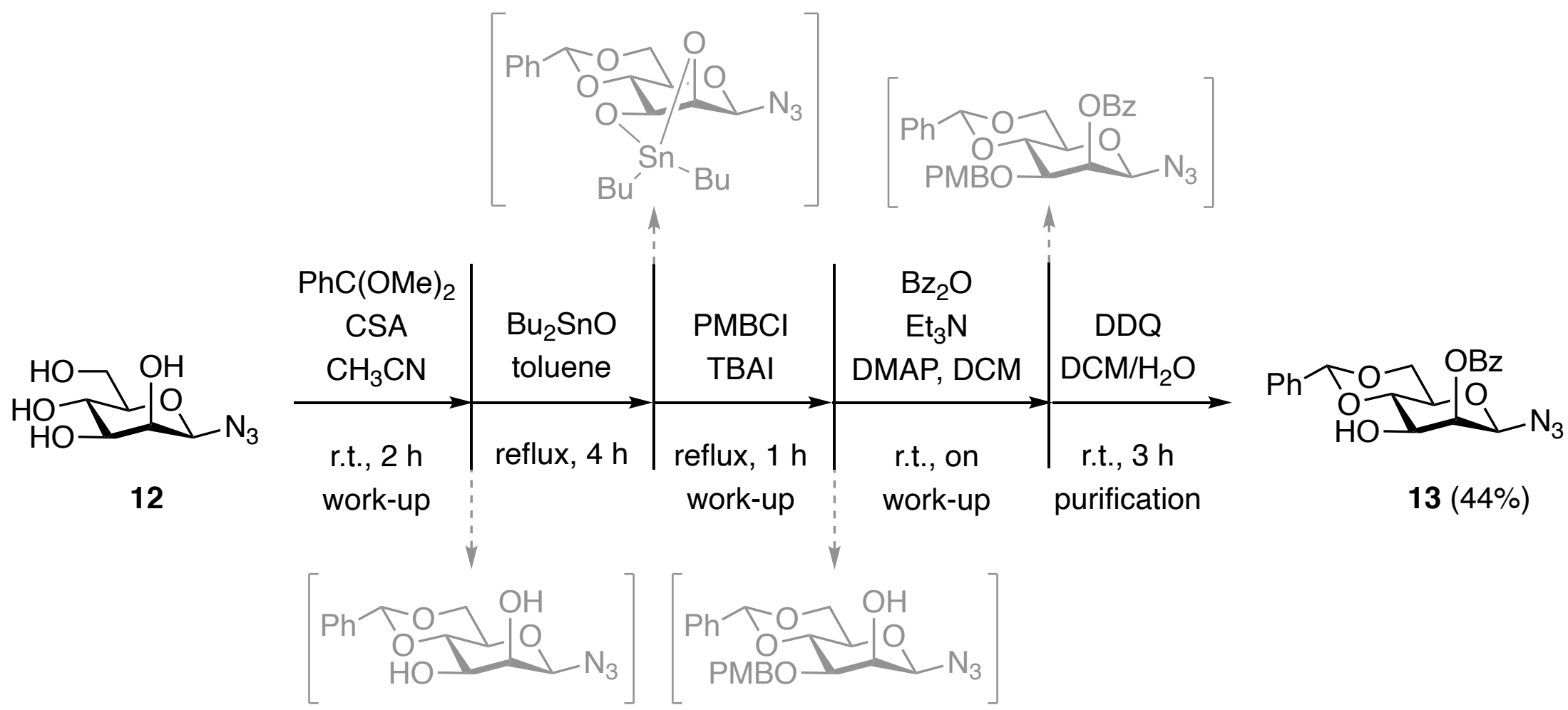

To a solution of unprotected β-D-mannopyranosyl azide **12** (211 mg, 1.03 mmol) in anhydrous ACN (3 mL), CSA (84 mg, 0.35 mmol) and benzaldehyde dimethyl acetal (0.17 mL, 1.12 mmol) were added. The reaction mixture was stirred at r.t. for 2 h and then quenched with Et_3N (0.3 mL). The mixture was diluted with EtOAc, and the organic phase was washed with H_2O. The aqueous phase was extracted with EtOAc (x2), and the combined organic layers were washed with brine, dried over anh. $MgSO_4$, filtered and concentrated. The resulting crude containing the 4,6-*O*-benzylidene derivative (276 mg, 1.11 mmol), Bu_2SnO (0.282 mg, 1.13 mmol) and 4 Å MS was dissolved in anhydrous toluene (10 mL) and then refluxed for 4 h. Then, the reaction mixture was cooled down to r.t. and PMBCl (0.17 mL, 1.20 mmol) and TBAI (436 mg, 1.16 mmol) were added. After refluxing for 1 h, the reaction mixture was filtered and washed with DCM. The organic phase was washed with H_2O (x2), dried over anh. $MgSO_4$, filtered and concentrated under vacuum. To the resulting mixture containing the 4,6-*O*-benzylidene-3-*O*-*p*-methoxybenzyl derivative in DCM (12 mL), Bz_2O (454 mg, 2.01 mmol), Et_3N (0.43 mL, 3.09 mmol) and a catalytic amount of DMAP were subsequently added. After stirring overnight at r.t., the reaction mixture was washed with 1M HCl, H_2O and brine. The organic phase was dried over anh. $MgSO_4$, filtered and the solvent was removed. To the crude containing the fully-protected mannoside derivative in DCM (12 mL) and H_2O (1 mL), DDQ (726 mg, 3.13 mmol) was added. The reaction mixture was stirred at r.t. for 3 h, and then it was diluted with DCM. The mixture was washed with H_2O (x3) and brine. The organic phase was dried over anh. $MgSO_4$, filtered and concentrated. The crude was purified by silica gel column chromatography (EtOAc/*n*-hexane, 1:4) to give compound **13** (179 mg, 44%) as a white amorphous solid.

Tetrasaccharide 15.

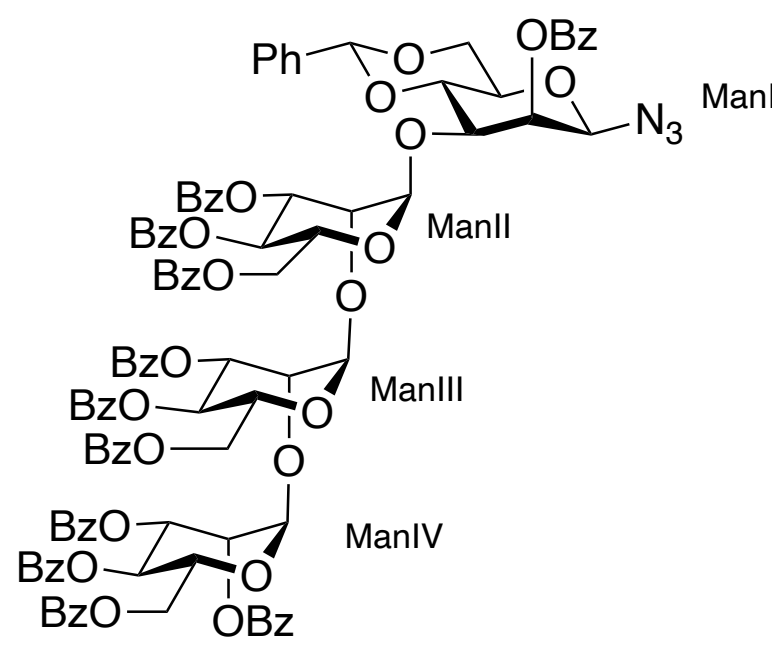

A mixture containing donor **14** (286 mg, 0.17 mmol), acceptor **13** (53 mg, 0.13 mmol) and 4Å MS in anhydrous DCM (7 mL) was stirred at 0 ºC for 15 min. Then, NIS (85 mg, 0.36 mmol) and TfOH (6 µL, 66.45 µmol) were added, and the reaction was stirred at 0 ºC for 2 h. The reaction was quenched with sat. $NaHCO_3$ aq. soln. (1 mL), filtered through celite and washed with DCM. The organic phase was washed with sat. $Na_2S_2O_3$ aq. soln. and then dried over anh. $MgSO_4$, filtered and concentrated. Purification of the crude by silica gel column chromatography (acetone/toluene, 1:60→1:50), afforded tetrasaccharide **15** (213 mg, 83%) as a white amorphous solid. ^{1}H NMR (400 MHz, $CDCl_3$) *δ*: 8.23 (m, 2H, H_{Ar}), 8.14 (m, 2H, H_{Ar}), 8.06 – 7.98 (m, 6H, H_{Ar}), 7.98 – 7.82 (m, 10H, H_{Ar}), 7.74 (m, 2H, H_{Ar}), 7.65 – 7.27 (m, 29H, H_{Ar}), 7.25 – 7.22 (m, 3H, H_{Ar}), 7.15 – 7.01 (m, 5H, H_{Ar}), 6.83 (m, 1H, H_{Ar}), 6.01 – 5.83 (m, 5H, H-4_{II}, H-3_{III}, H-4_{III}, H-3_{IV}, H-4_{IV}), 5.79 – 5.73 (m, 2H, H-2_{I}, H-2_{IV}), 5.58 (dd, $^3J_{H3-H4}$ = 9.2, $^3J_{H3-H2}$ = 3.2, 1H, H-3_{II}), 5.52 (m, 1H, H-1_{II}), 5.34 (br s, 1H, H_{acetal}), 5.29 (m, 1H, H-1_{III}), 4.92 (br s, 1H, H-1_{IV}), 4.83 (m, 1H, H-5_{II}), 4.70 (m, 2H, H-6_{II}), 4.49 (d, $^3J_{H1-H2}$ = 0.9, 1H, H-1_{I}), 4.42 – 4.32 (m, 3H, H-2_{II}, H-$6a_{I}$, H-2_{III}), 4.31-4.13 (m, 4H, H-5_{III}, H-5_{IV}, H-6, H-3_{I}), 4.11 – 3.96 (m, 3H, H-4_{I}, 2 x H-6), 3.91– 3.79 (m, 2H, H-$6b_{I}$, H-6), 3.36 (m, 1H, H-5_{I}); ^{13}C NMR (101 MHz, $CDCl_3$) *δ*: 166.4 (CO), 165.9 (CO), 165.8 (CO), 165.6 (CO), 165.5 (CO), 165.4 (CO), 165.3 (CO), 165.1 (CO), 164.8 (CO), 136.7 (C_{Ar}), 133.8 (C_{Ar}), 133.5 (C_{Ar}), 133.4 (C_{Ar}), 133.3 (C_{Ar}), 133.2 (C_{Ar}), 132.9 (C_{Ar}), 132.9 (C_{Ar}), 130.3 (C_{Ar}), 130.2 – 129.6 (C_{Ar}), 130.1 (C_{Ar}), 129.4 (C_{Ar}), 129.3 (C_{Ar}), 129.2 (C_{Ar}), 129.2 (C_{Ar}), 129.1 (C_{Ar}), 129.1 (C_{Ar}), 128.9 (C_{Ar}), 128.8 – 128.1 (C_{Ar}), 125.8 (C_{Ar}), 101.8 (C_{acetal}), 99.8 (C-1_{III}), 99.7 (C-1_{IV}), 99.6 (C-1_{III}), 86.5 (C-1_{I}), 78.2 (C-4_{I}), under $CDCl_3$ (C-2_{III}), 75.5 (C-2_{II}), 72.9 (C-3_{I}), 71.6 (C-2_{I}), 71.0 (C-3_{II}), 70.3, 70.0, 69.8, 69.7, 69.7, 69.3, 68.3 (C-6_{I}), 67.6, 67.2, 66.4 (C-6_{II}), 64.1, 63.3, 62.6; ESI-MS: *m/z* calcd. for $C_{108}H_{89}N_3O_{31}$: 1923.5; found: 1947.2 $[M+Na]^+$; ESI-HRMS: *m/z* calcd. for $C_{108}H_{89}N_3O_{31}Na$ $[M+Na]^+$: 1946.5372; found: 1946.5367.

Tetrasaccharide 16.

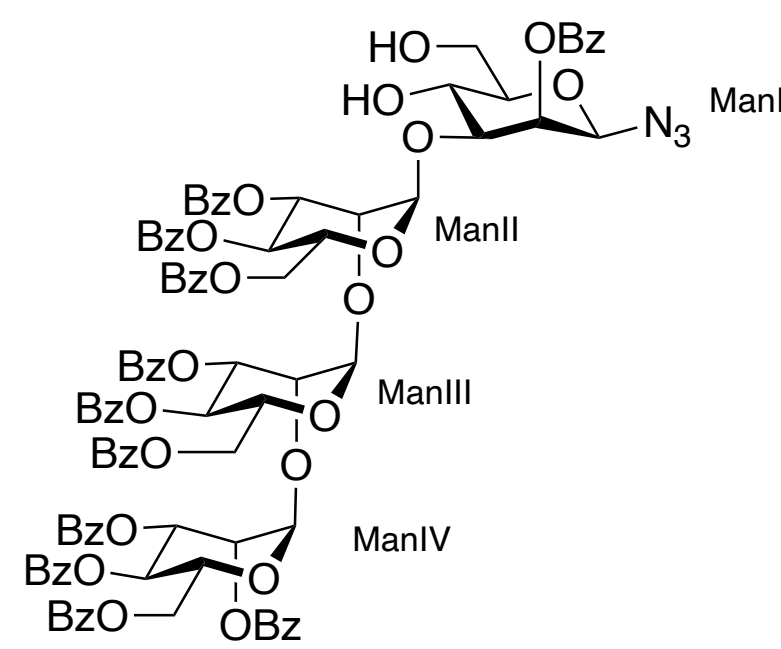

A solution of tetrasaccharide **15** (280 mg, 0.15 mmol) and PTSA·H_2O (56 mg, 0.29 mmol) in ACN (7 mL) was stirred overnight at 40 ºC. The reaction mixture was quenched with Et_3N (0.2 mL) and concentrated under vacuum. The crude was purified by silica gel column chromatography (EtOAc/*n*-hexane, 1:2→1:1) to give tetrasaccharide **16** (248 mg, 93%) as a white amorphous solid. ^{1}H NMR (400 MHz, $CDCl_3$) *δ*: 8.16 (m, 2H, H_{Ar}), 8.05 – 7.93 (m, 14H, H_{Ar}), 7.91 (m, 2H, H_{Ar}), 7.88 – 7.82 (m, 4H, H_{Ar}), 7.59 – 7.27 (m, 33H, H_{Ar}), 6.14 (t, $^3J_{H4\text{-}H3}$ = $^3J_{H4\text{-}H5}$ = 10.1, H-4_{IV}), 5.99 – 5.86 (m, 3H, H-3_{III}, H-3_{IV}, H-4_{IV}), 5.80 – 5.69 (m, 3H, H-2_{IV}, H-4_{II}, H-2_{I}), 5.60 – 5.55 (m, 2H, H-3_{II}, H-1_{II}), 5.48 (m, 1H, H-1_{III}), 5.07 (br s, 1H, H-1_{IV}), 4.77 – 4.63 (m, 4H, H-5**II**, 3 x H-6), 4.57 (m, 1H, H-1_{I}), 4.52 – 4.37 (m, 4H, H-2_{II}, H-5_{IV}, H-5, H-6), 4.31 – 4.20 (m, 3H, H-2_{III}, H-4_{I}, H-5), 4.15 – 3.89 (m, 5H, H-6, 2 x H-6_{I}, H-3_{I}, OH-4), 3.40 (m, 1H, H-5_{I}), 2.23 (t, $^3J_{OH\text{-}H6a}$ = $^3J_{OH\text{-}H6b}$ = 6.7, OH-6); ^{13}C NMR (101 MHz, $CDCl_3$) *δ*: 166.4 (CO), 166.0 (CO), 165.6 (CO), 165.5 (CO), 165.4, (CO) 165.4 (CO), 165.1 (CO), 164.9 (CO), 133.5 (C_{Ar}), 133.4 (C_{Ar}), 133.1 (C_{Ar}), 130.1 (C_{Ar}), 130.1 – 129.7 (C_{Ar}), 129.7 (C_{Ar}), 129.2 (C_{Ar}), 129.2 (C_{Ar}), 129.1 (C_{Ar}), 128.9 (C_{Ar}), 128.8 (C_{Ar}), 128.7 – 128.4 (C_{Ar}), 128.3 (C_{Ar}), 100.4 (C-1_{II}), 99.3 (C-1_{IV}), 98.1 (C-1_{III}), 85.9 (C-1_{I}), 78.7 (C-5_{I}), 77.7 (C-3_{I}), under $CDCl_3$ (C-2_{III}) 74.2 (C-2_{II}), 71.8 (C-2_{I}), 70.4, 70.2, 69.9, 69.7, 69.5, 68.6, 67.8, 67.7, 66.5 (C-4_{IV}), 63.9, 63.6, 62.6, 62.0 (C-6_{I}); ESI-MS: *m/z* calcd. for $C_{101}H_{85}N_3O_{31}$: 1835.5; found: 1859.3 $[M+Na]^+$; ESI-HRMS: *m/z* calcd. for $C_{101}H_{85}N_3O_{31}Na$ $[M+Na]^+$: 1858.5059; found: 1858.5046.

Nonasaccharide 18

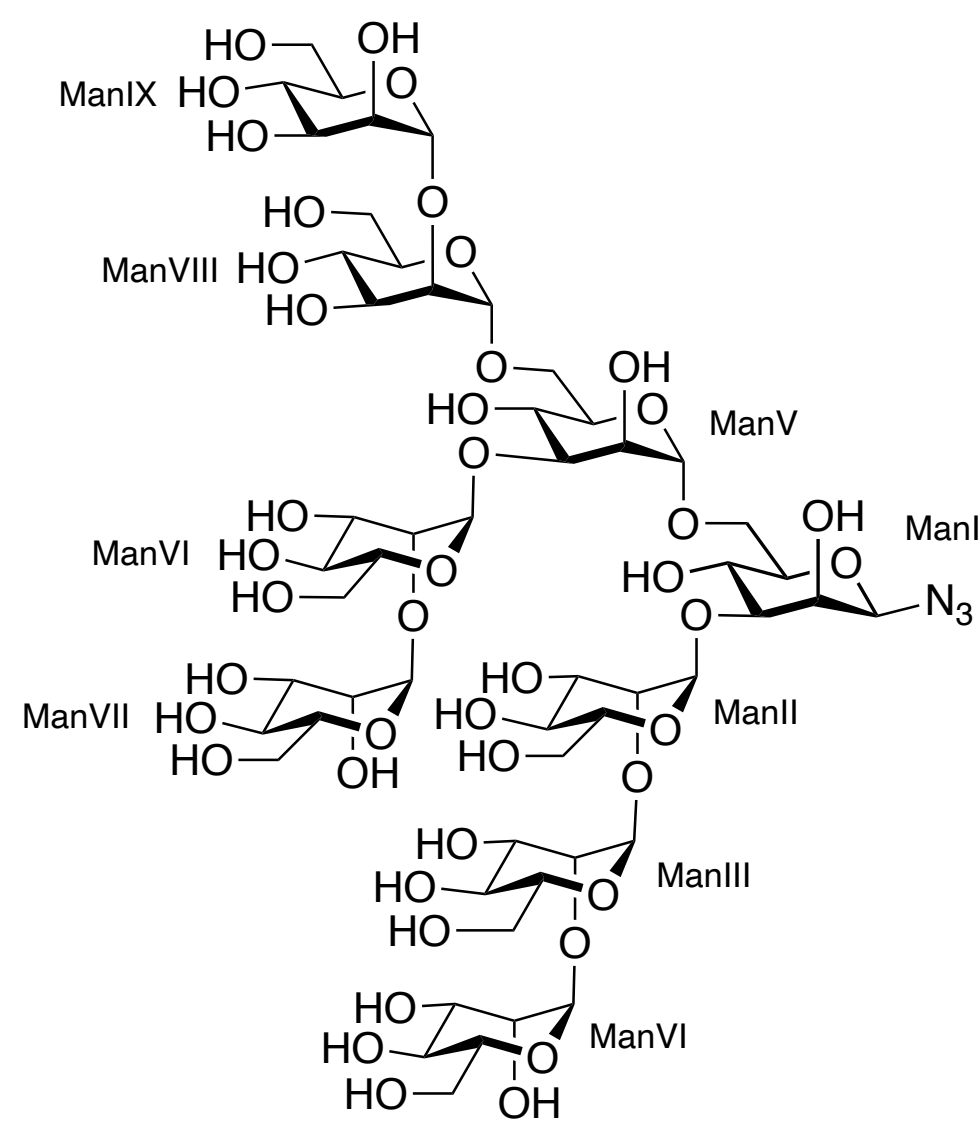

A mixture containing acceptor **16** (23.4 mg, 12.74 µmol), pentasaccharide **17** (43.8 mg, 16.84 µmol) and 4Å MS in anhydrous DCM (2 mL) was stirred at 0 ºC for 15 min. Then, NIS (17.6 mg, 74.32 µmol) and TfOH (1.0 µL, 11.07 µmol) were added. The reaction mixture was stirred at 0 ºC for 1 h, and then it was quenched with sat. $NaHCO_3$ aq. soln. (0.5 mL). The mixture was filtered through celite and washed with DCM. The organic phase was washed with sat. $Na_2S_2O_3$ aq. soln. and then dried over anh. $MgSO_4$, filtered and concentrated. The crude was purified by silica gel column chromatography (acetone/toluene, 1:15) to give the corresponding nonasaccharide as a white amorphous solid. To a solution of the resulting nonasaccharide in MeOH/toluene (4:1, 1 mL), NaOMe (3.7 mg, 0.73 mmol) was added and the reaction was stirred at r.t. for 1 h. Then, 1M NaOH solution (0.4 mL) was added and the reaction mixture was heated at 50 ºC for 6 h. After neutralization with Amberlite IR-120 (H^+) resin, the solution was filtered and concentrated *in vacuo*. The crude was purified by size-exclusion chromatography (Sephadex G25, H_2O/MeOH, 9:1), furnishing unprotected nonasaccharide **18** (16 mg, 84%) as a white amorphous solid. ^{1}H NMR (400 MHz, D_2O) δ: 5.41 (d, $^3J_{H1\text{-}H2}$ = 1.5, 1H, H-1_{III} or H-1_{VI}), 5.35 (d, $^3J_{H1\text{-}H2}$ = 1.3, 1H, H-1_{II}), 5.31 (d, $^3J_{H1\text{-}H2}$ = 1.6, 1H, H-1_{III} or H-1_{VI}), 5.15 (d, $^3J_{H1\text{-}H2}$ = 1.6, 1H, H-1_{VIII}), 5.06 – 5.04 (m, 3H, H-1_{IV}, H-1_{VII}, H-1_{IX}), 4.92 (m, 1H, H-1_{I}), 4.88 (d, $^3J_{H1\text{-}H2}$ = 1.6, 1H, H-1_{V}), 4.18 – 4.14 (m, 2H, H-2_{V}, H-2_{I}), 4.12 – 4.06 (m, 6H, H-2_{II}, H-2_{III}, H-2_{IV}, H-2_{VI}, H-2_{VII}, H-2_{IX}), 4.05 – 3.60 (m, 46H); ^{13}C NMR (101 MHz, D_2O) δ: 102.3 (C-1_{IV}, C-1_{VII} or C-1_{IX}), 102.2 (C-1_{IV}, C-1_{VII} or C-1_{IX}), 102.2 (C-1_{IV}, C-1_{VII} or C-1_{IX}), 100.7 (C-1_{II}, C-1_{III} or C-1_{VI}), 100.7 (C-1_{II}, C-1_{III} or C-1_{VI}), 100.6 (C-1_{II}, C-1_{III} or C-1_{VI}), 99.6 (C-1_{V}), 98.0 (C-1_{VIII}), 87.4 (C-1_{I}), 80.8 (C-3_{I}), 78.9 (C-3_{V}), 78.6, 78.5, 76.2, 73.3, 73.3 – 73.1, 72.7, 71.2, 70.5, 70.3, 70.1 – 69.9, 69.9, 69.5, 67.0 – 66.7, 66.9, 66.8, 65.6, 65.4, 65.2, 61.3 – 60.7; ESI-MS: *m/z* calcd. for $C_{54}H_{91}N_3O_{45}$: 1501.5; found: 1524.1 $[M+Na]^+$; ESI-HRMS: *m/z* calcd. for $C_{54}H_{91}N_3O_{45}Na$ $[M+Na]^+$: 1524.4817; found: 1524.4810.

Nonasaccharide 11

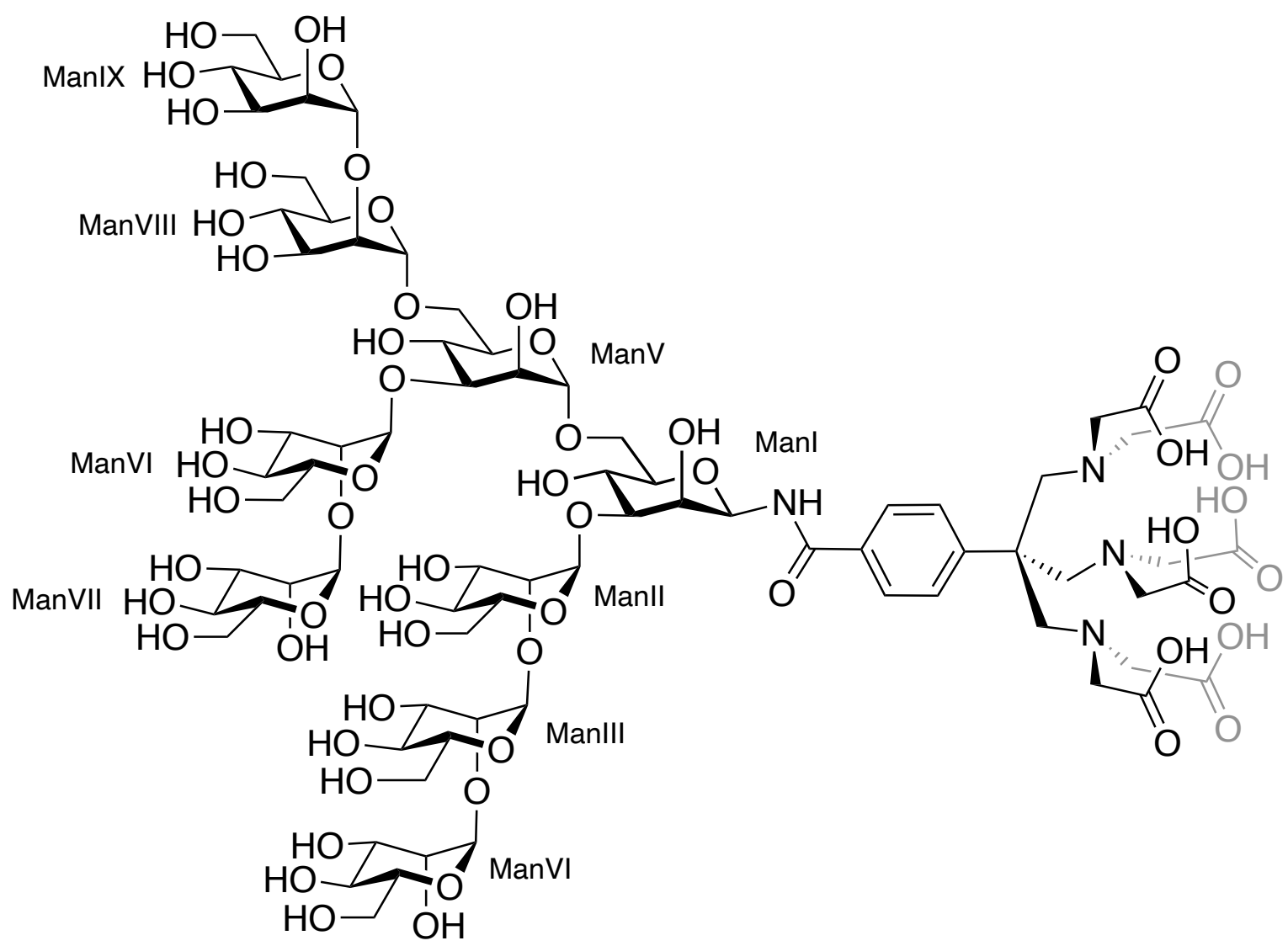

Nonasaccharide **18** (106.7 mg, 71.02 μmol) was dissolved in MeOH/HCl 1M solution (9:1, 9 mL) and Pd/C (10%, 9.7 mg) was added. After stirring at r.t. for 2 h under H_2 atmosphere (1 bar), the reaction mixture was filtered over celite, washed with MeOH and concentrated. Then, to a mixture containing the nonasaccharide derivative in anhydrous DIPEA (270 μL, 1.54 mmol) and anhydrous DMF (4.5 mL), a mixture of the chelating tag **19** (172.2 mg, 0.19 mmol), HATU (75.5 mg, 0.19 mmol) and HOBt (26.8 mg, 0.19 mmol) in anhydrous DMF (2.4 mL) was added. The reaction mixture was stirred at r.t. for 24 h and concentrated. The crude was purified by size-exclusion chromatography (Sephadex LH20, MeOH) to give the corresponding amide derivative. The resulting amide was dissolved in TFA/H_2O 9:1 (1 mL) and stirred at r.t. for 24 h. After removal of the solvent, the crude was purified by size-exclusion chromatography (Sephadex G25, H_2O/MeOH 9:1), followed by HPLC (Luna® 10 μm C_{18} 100Å, MiliQ H_2O 0.1% formic acid/MeOH 98:2, isocratic, flow rate 3 mL/min, t_R 7.4 min), affording compound **11** (11.5 mg, 9% over 3 steps) as a white amorphous solid. ^1H NMR (500 MHz, D_2O) δ: 7.89 (d, $^3J_{Ho\text{-}Hm}$ = 7.9, 2H, $H_{o\text{-Ph}}$), 7.76 (d, $^3J_{Hm\text{-}Ho}$ = 8.1, 2H, $H_{m\text{-Ph}}$), 5.46 (m, 1H, H-1_{I}), 5.42 – 5.40 (m, 2H, H-1_{III} or H-1_{VI}, H-1_{II}), 5.32 (m, 1H, H-1_{III} or H-1_{VI}), 5.13 (m, 1H, H-1_{VIII}), 5.06 – 5.02 (m, 3H, H-1_{IV}, H-1_{VII}, H-1_{IX}), 4.86 (m, 1H, H-1_{V}), 4.23 (m, 1H, H-2_{I}), 4.17 – 4.06 (m, 7H, H-2_{II}, H-2_{III}, H-2_{IV}, H-2_{V}, H-2_{VI}, H-2_{VII}, H-2_{IX}), 4.05 – 3.58 (m, 64H); ^{13}C NMR (126 MHz, D_2O) δ: 173.0 (COOH), 169.6 (CONH), 143.7 ($C_{ipso\text{-Ph}}$), 132.3 ($C_{p\text{-Ph}}$), 128.5 ($C_{o\text{-Ph}}$), 127.1 ($C_{m\text{-Ph}}$), 102.3 (C-1_{IV}, C-1_{VII}, or C-1_{IX}), 102.2 (C-1_{IV}, C-1_{VII}, or C-1_{IX}), 100.7 (C-1_{II}, C-1_{III}, or C-1_{VI}), 100.6 (C-1_{II}, C-1_{III}, or C-1_{VI}), 100.5 (C-1_{II}, C-1_{III}, or C-1_{VI}), 99.6 (C-1_{V}), 98.0 (C-1_{VIII}), 81.0 (C-3_{I}), 78.8, 78.7, 78.6, 78.6, 78.5 (C-1_{I}), 75.8, 73.4, 73.3, 73.2, 73.2, 72.7, 71.1, 70.3, 70.2, 70.1, 70.0, 70.0, 69.9, 69.8, 69.4 (C-2_{I}), 67.0, 66.9, 66.9, 66.9, 66.8, 66.8, 65.6, 65.4, 65.2, 61.1, 61.0, 60.9, 56.4 (*C*H_2COOH), 46.2 (Ph*C*($CH_2NR_2)_3$); ESI-MS: *m/z* calcd. for $C_{77}H_{120}N_4O_{58}$: 2028.7; found: 1026.9 $[M+H+Na]^{2+}$; ESI-HRMS: *m/z* calcd. for $C_{77}H_{121}N_4O_{58}Na$ $[M+Na+H]^{2+}$: 1026.3264; found: 1026.3295.

3. NMR spectra

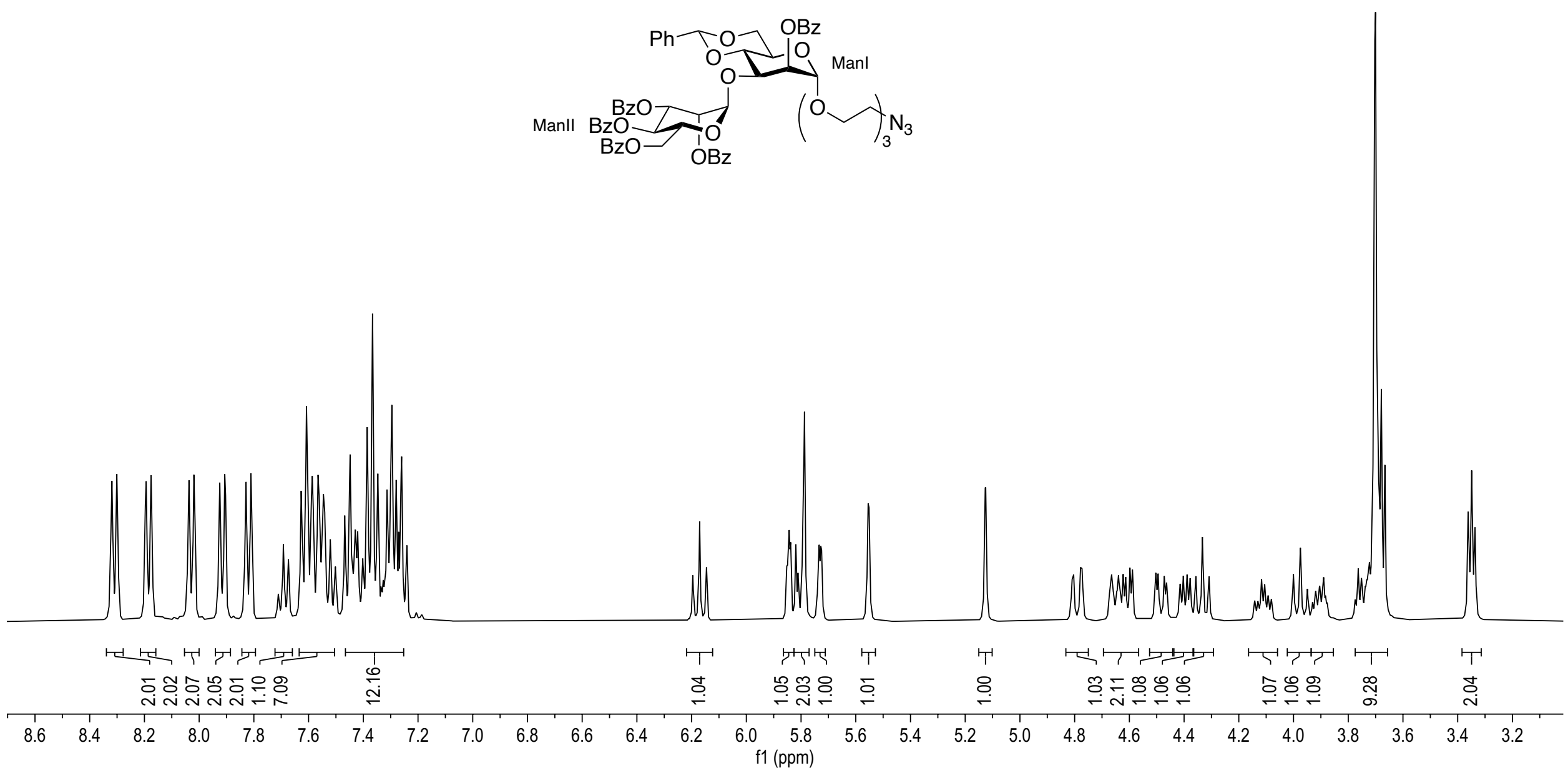

Figure S1. 1D ^{1}H NMR spectrum of **5** (400 MHz, $CDCl_3$).

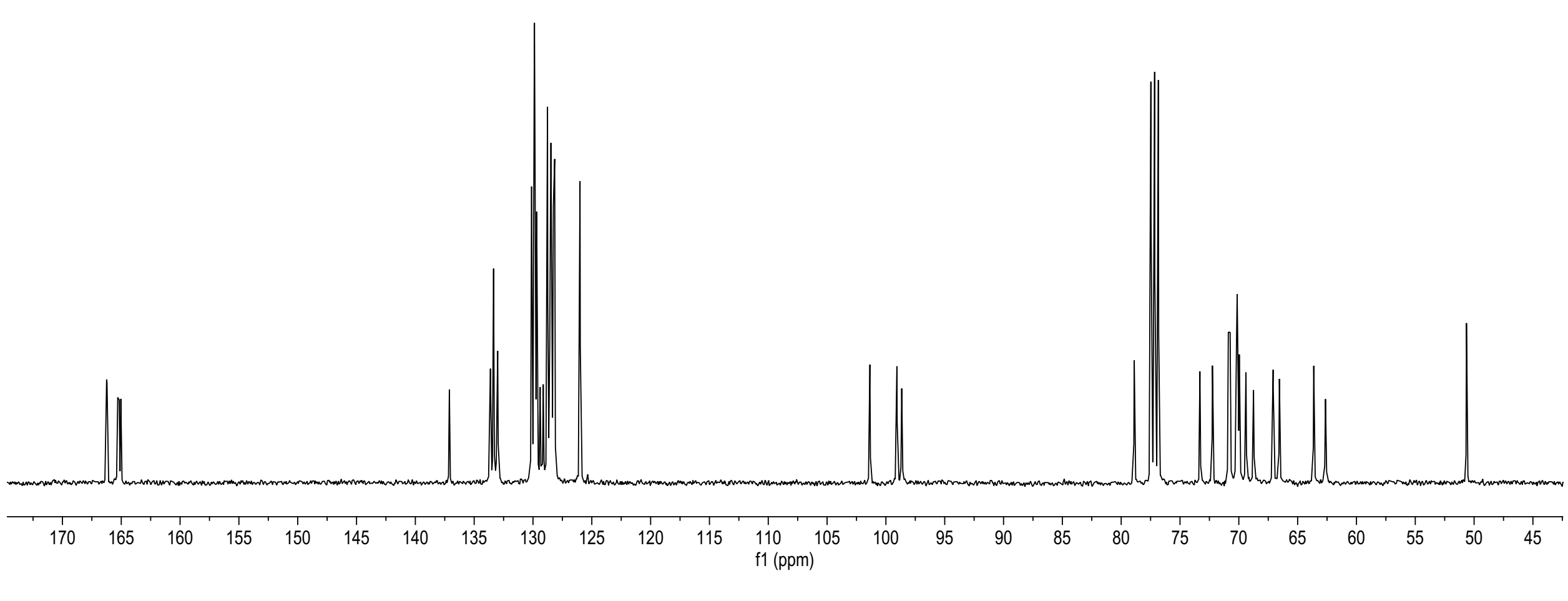

Figure S2. 1D $^{13}C\{^{1}H\}$ NMR spectrum of **5** (101 MHz, $CDCl_3$).

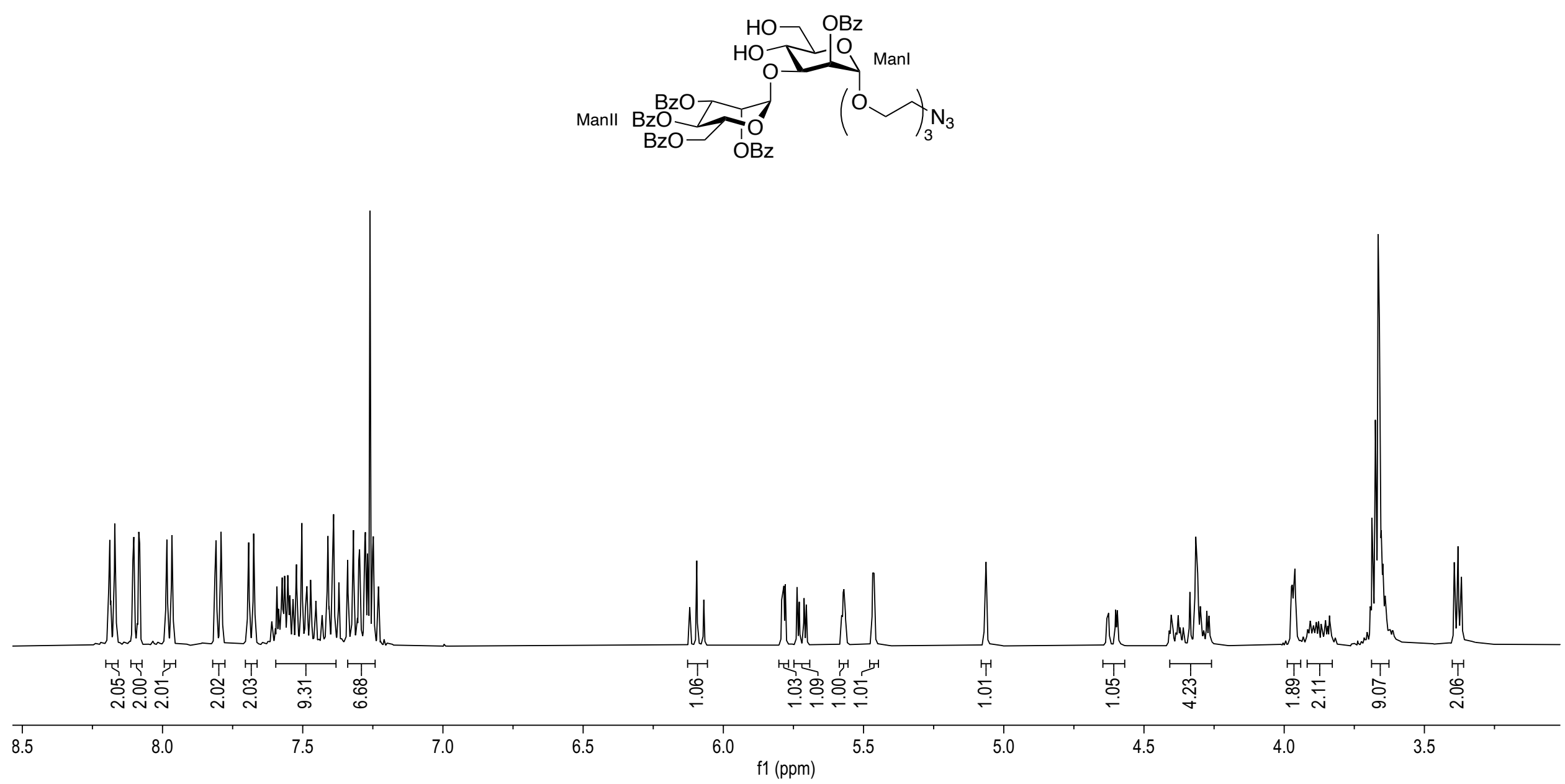

Figure S3. 1D ^{1}H NMR spectrum of **6** (400 MHz, $CDCl_3$).

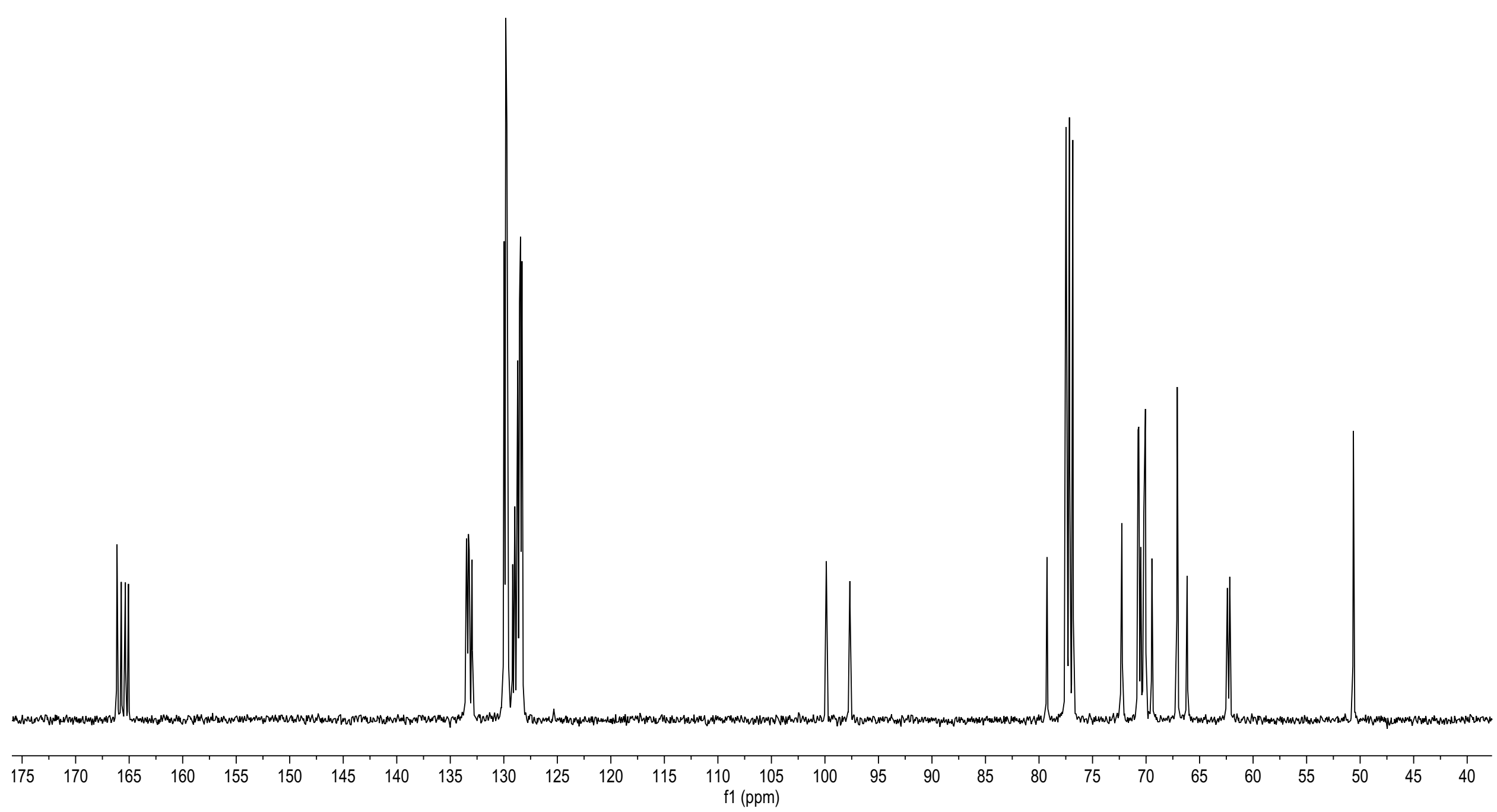

Figure S4. 1D $^{13}C\{^{1}H\}$ NMR spectrum of **6** (101 MHz, $CDCl_3$).

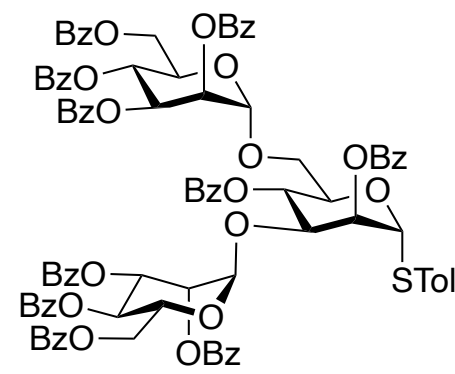

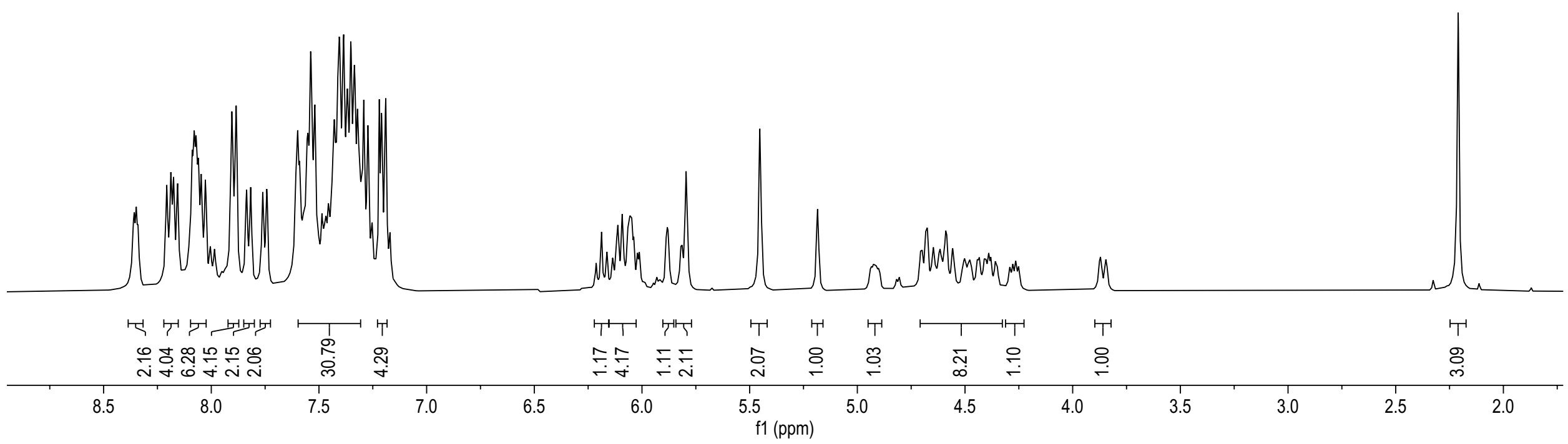

Figure S5. 1D ^{1}H NMR spectrum of **9** (400 MHz, $CDCl_3$).

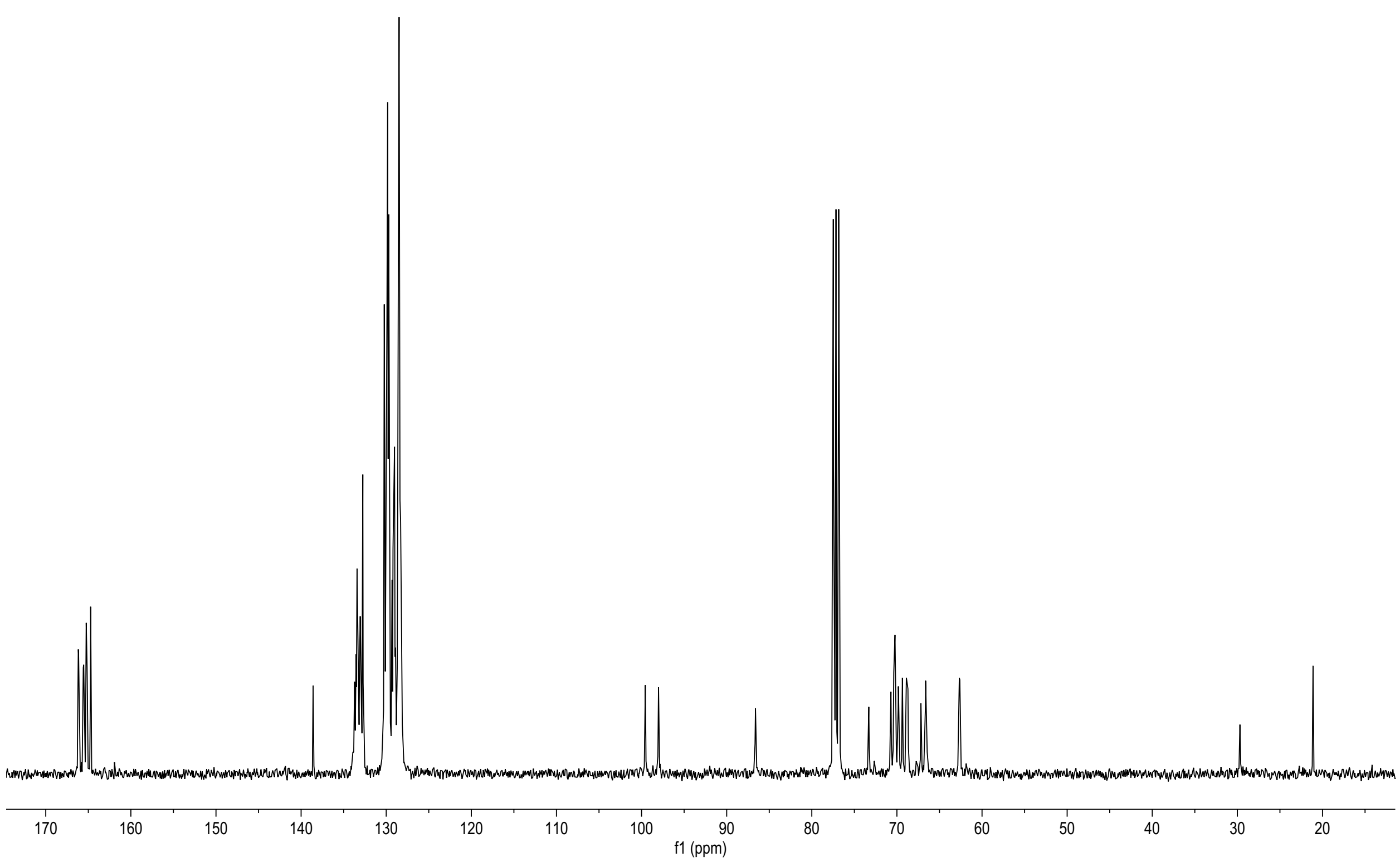

Figure S6. 1D ^{13}C{^{1}H} NMR spectrum of **9** (101 MHz, $CDCl_3$).

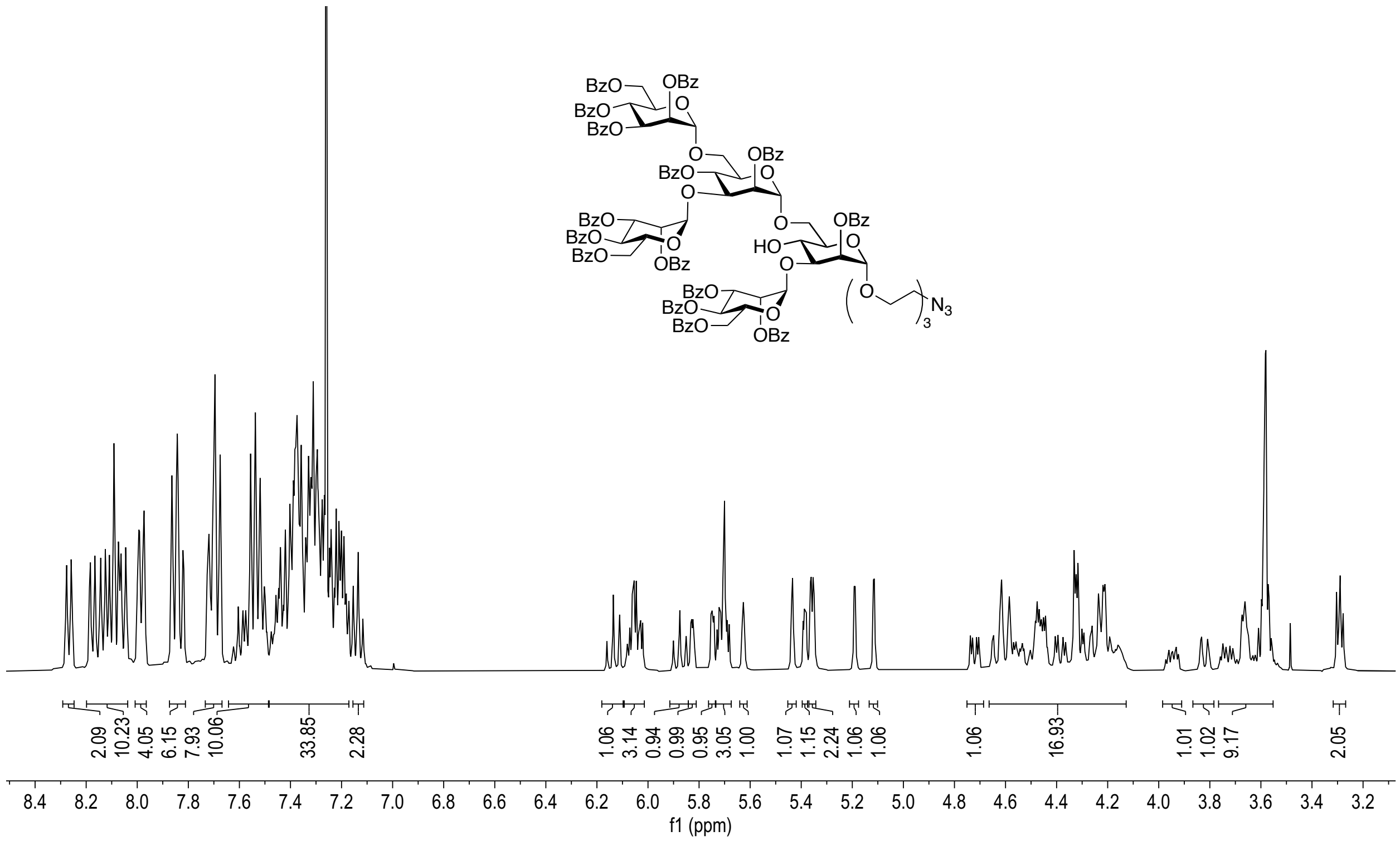

Figure S7. 1D ^{1}H NMR spectrum of **10** (400 MHz, $CDCl_3$).

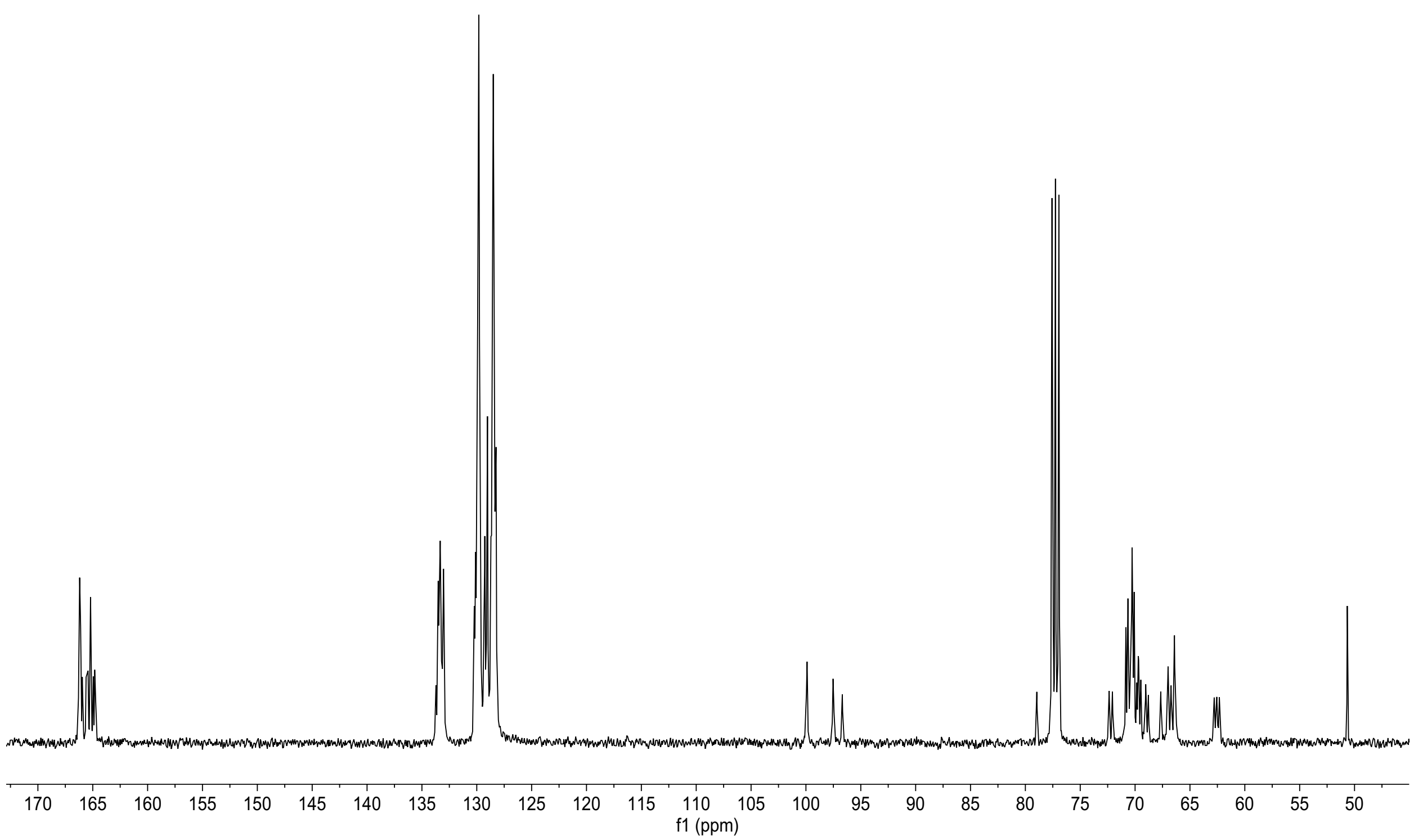

Figure S8. 1D $^{13}C\{^{1}H\}$ NMR spectrum of **10** (101 MHz, $CDCl_3$).

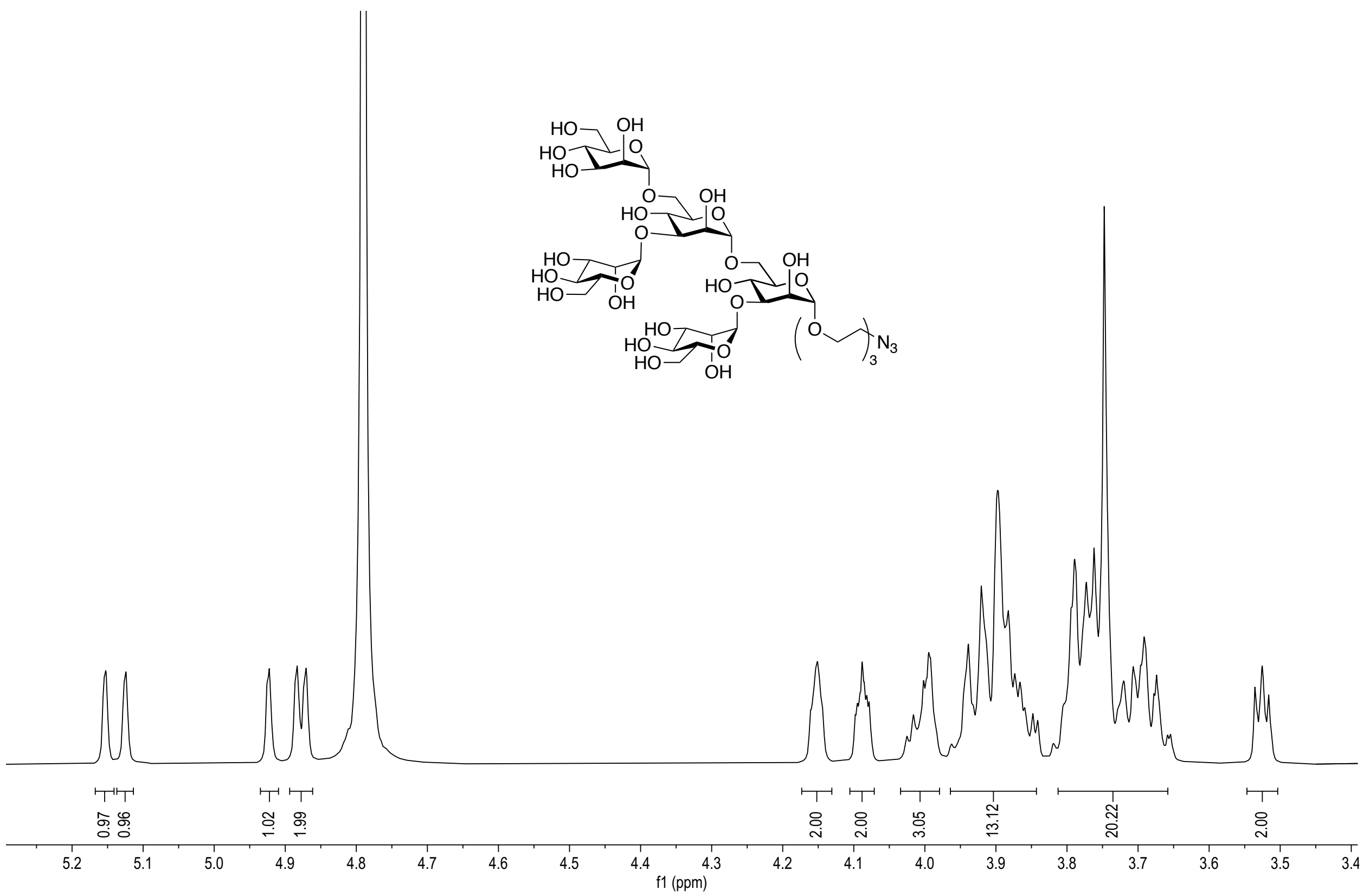

Figure S9. 1D $^{13}C\{^{1}H\}$ NMR spectrum of **1** (500 MHz, D_2O).

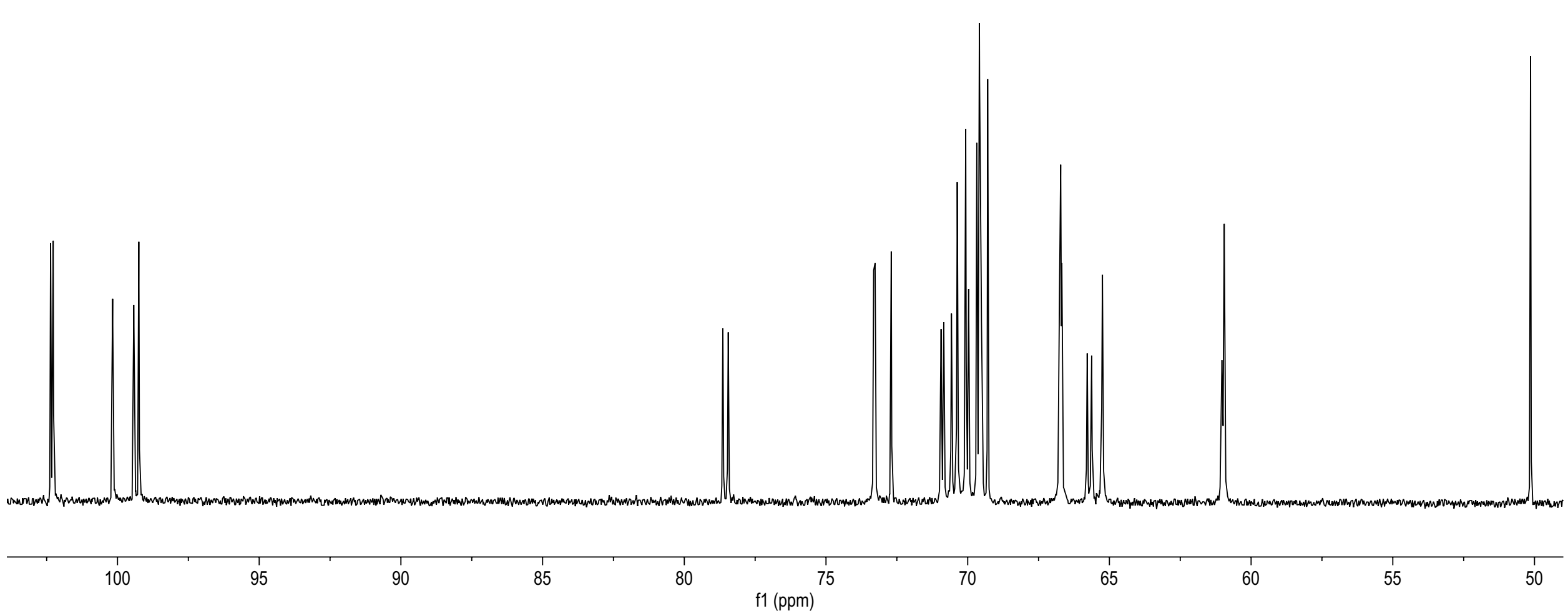

Figure S10. 1D $^{13}C\{^{1}H\}$ NMR spectrum of **1** (126 MHz, D_2O).

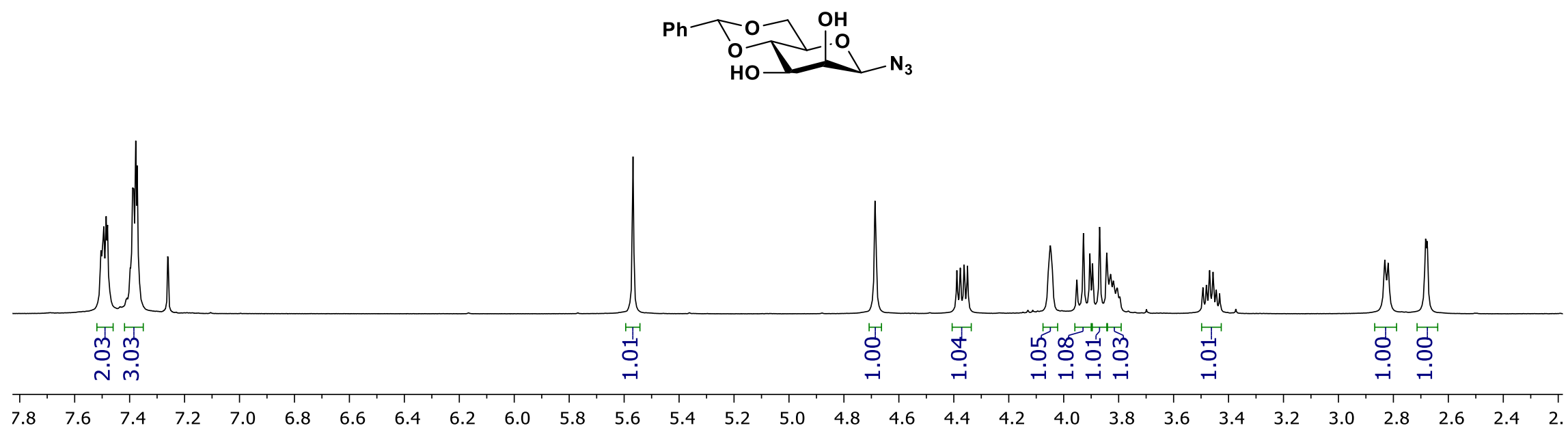

Figure S11. ^{1}H NMR spectrum of **20** (400 MHz, $CDCl_3$).

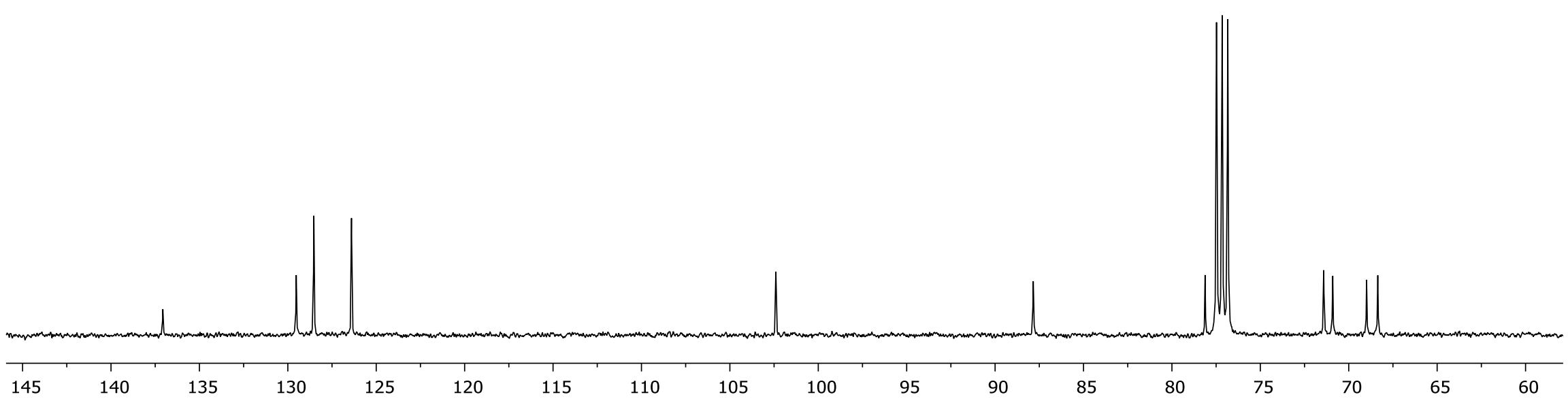

Figure S12. 1D $^{13}C\{^1H\}$ NMR spectrum of **20** (101 MHz, $CDCl_3$).

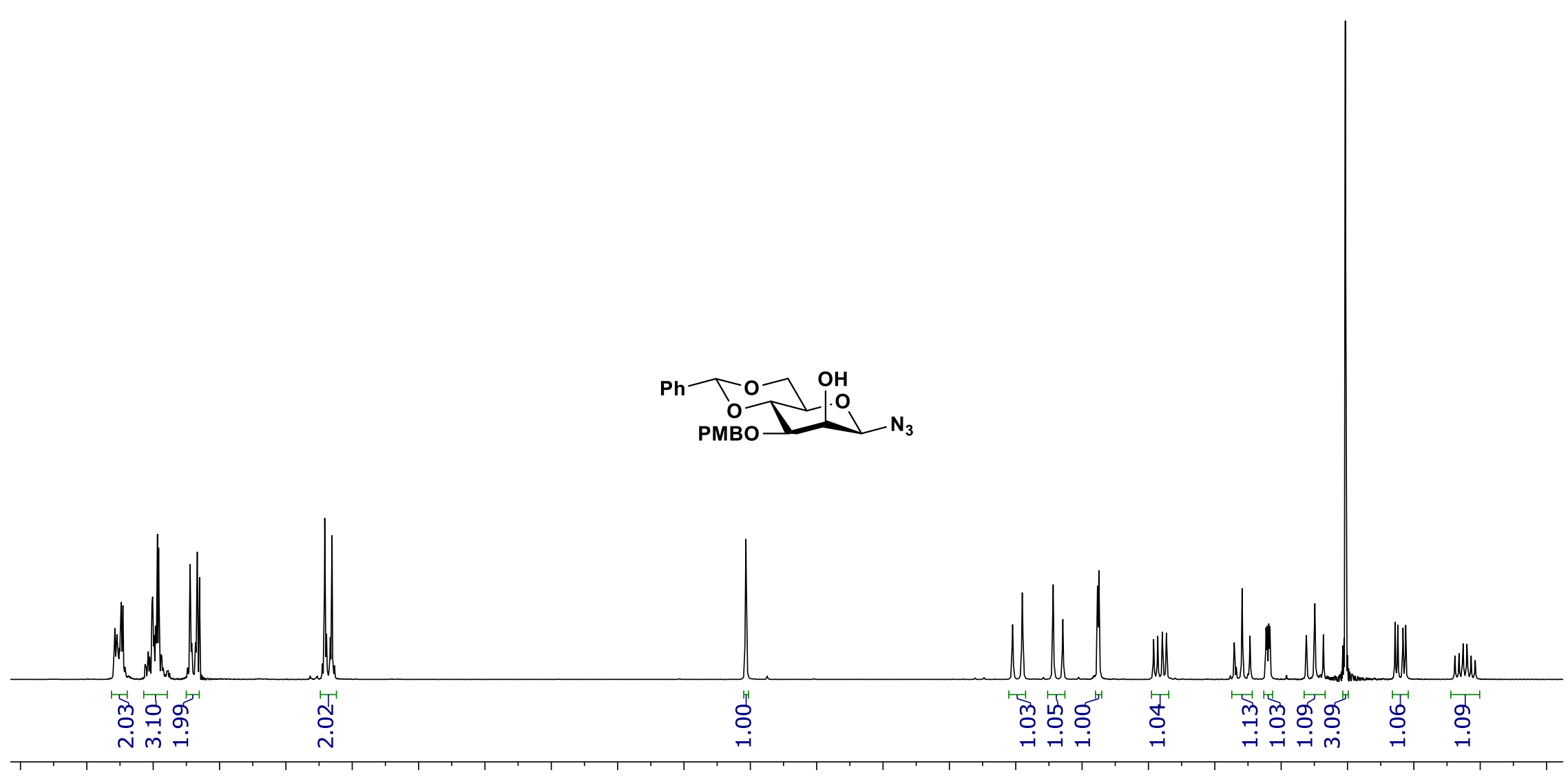

Figure S13. 1D ^{1}H NMR spectrum of **21** (400 MHz, $CDCl_3$).

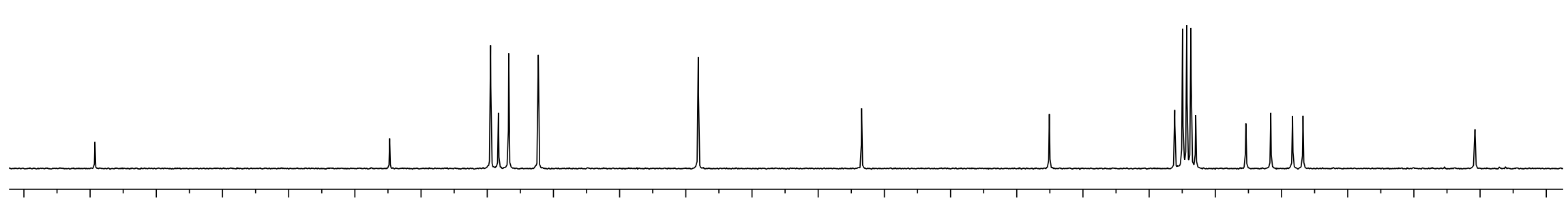

Figure S14. 1D $^{13}C\{^1H\}$ NMR spectrum of **21** (101 MHz, $CDCl_3$).

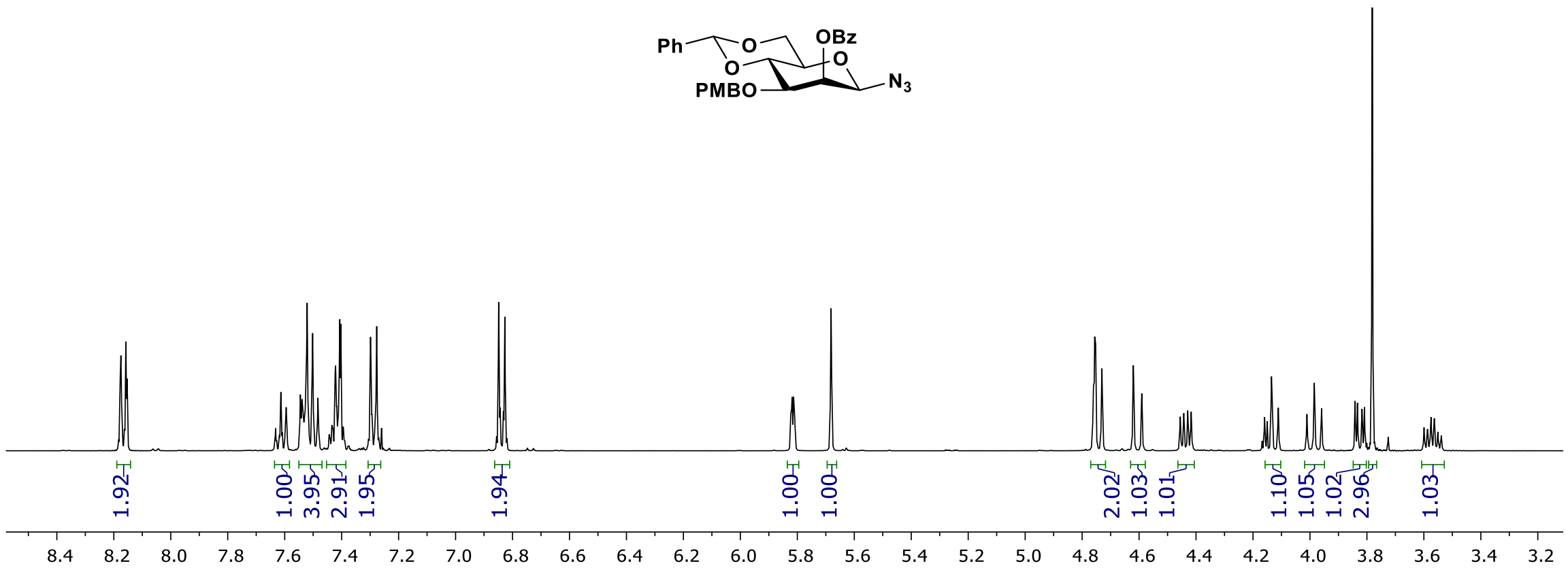

Figure S15. 1D ^{1}H NMR spectrum of **22** (400 MHz, $CDCl_3$).

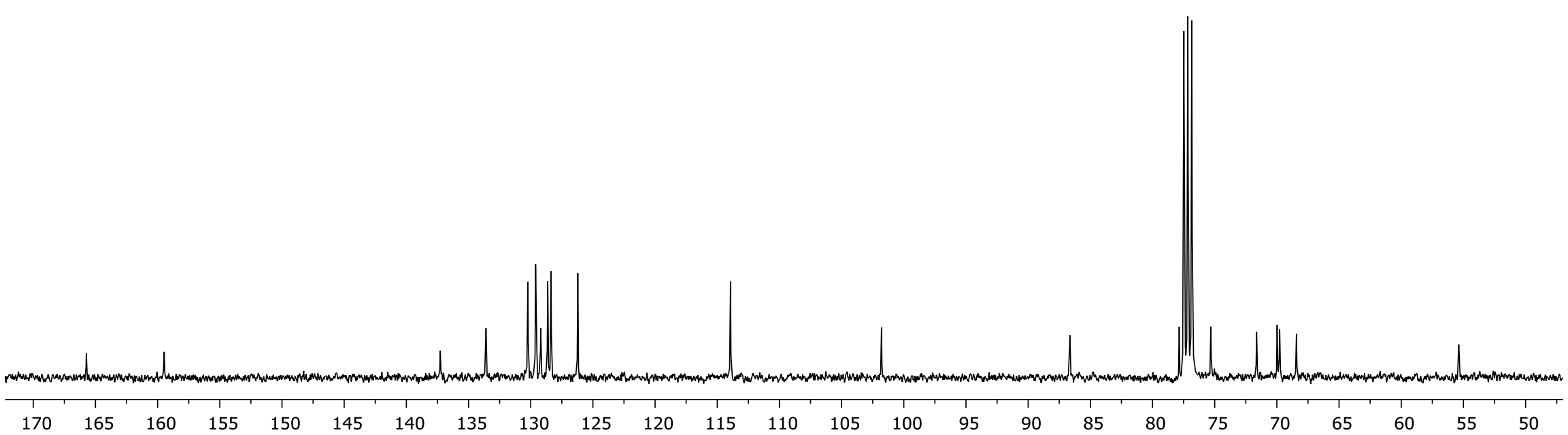

Figure S16. 1D $^{13}C\{^{1}H\}$ NMR spectrum of **22** (101 MHz, $CDCl_3$).

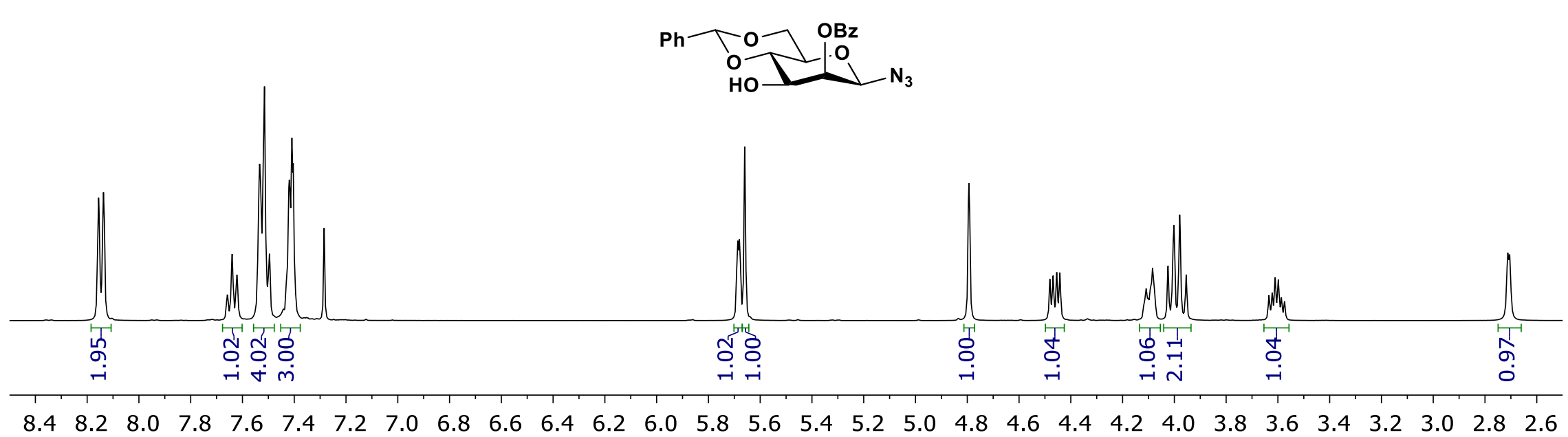

Figure S17. 1D 1H NMR spectrum of **13** (400 MHz, $CDCl_3$).

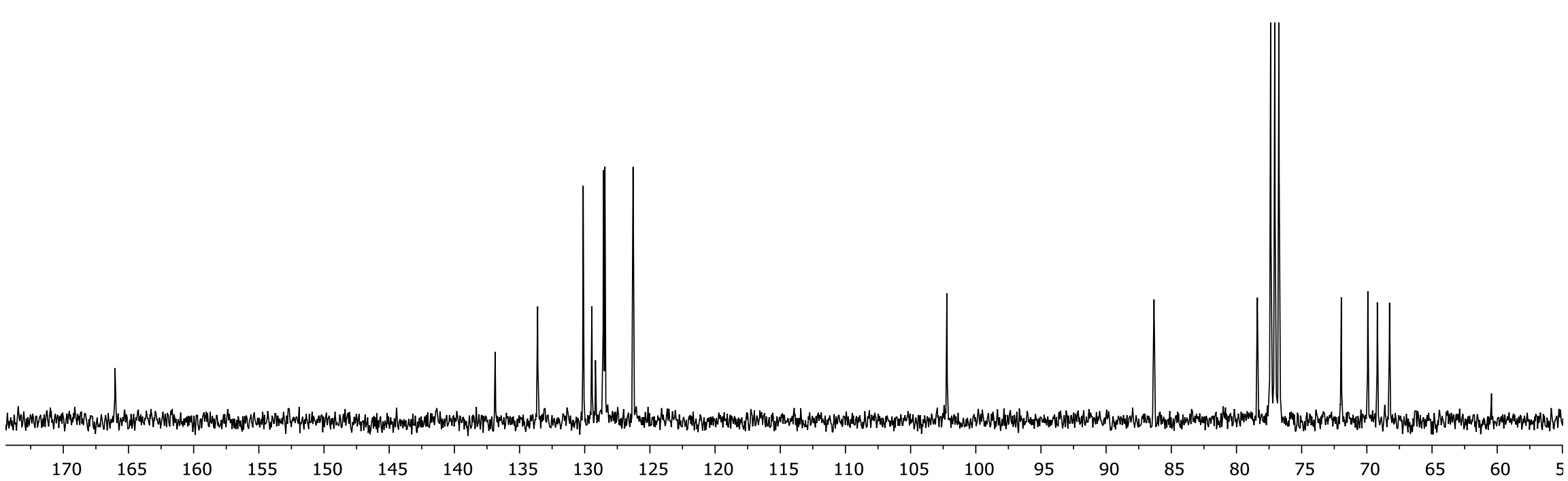

Figure S18. 1D $^{13}C\{^{1}H\}$ NMR spectrum of **13** (101 MHz, $CDCl_3$).

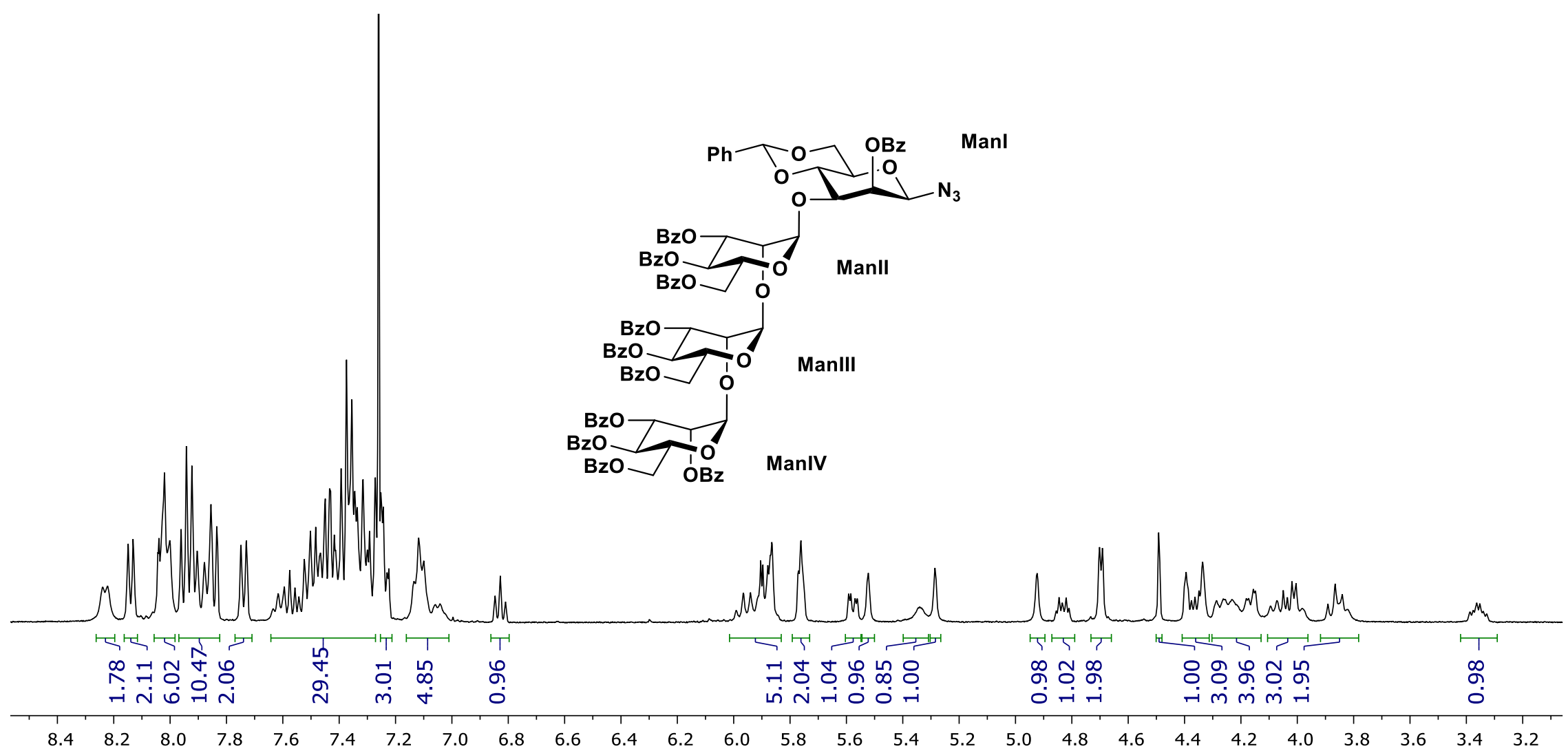

Figure S19. 1D ^{1}H NMR spectrum of **15** (400 MHz, $CDCl_3$).

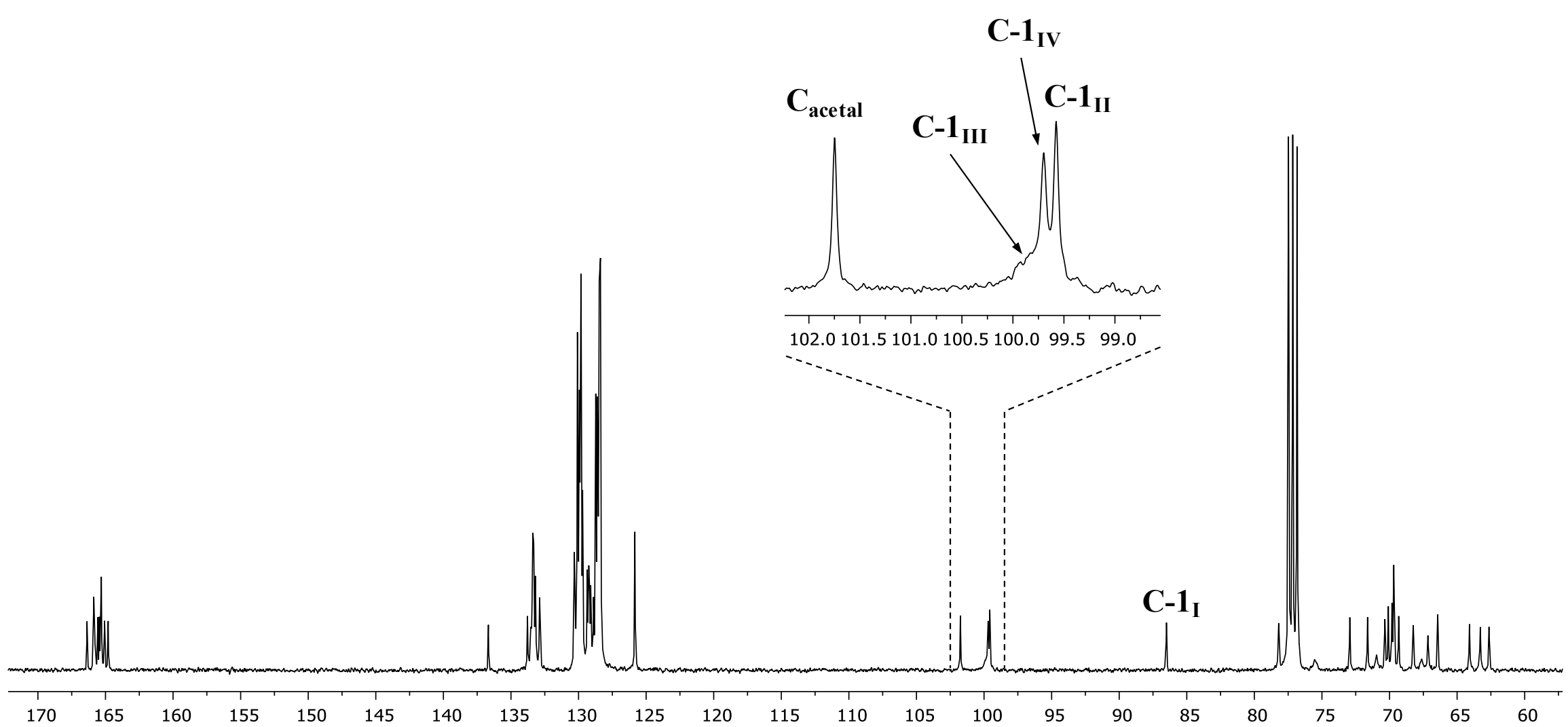

Figure S20. 1D ^{13}C{^{1}H} NMR spectrum of **15** (101 MHz, $CDCl_3$).

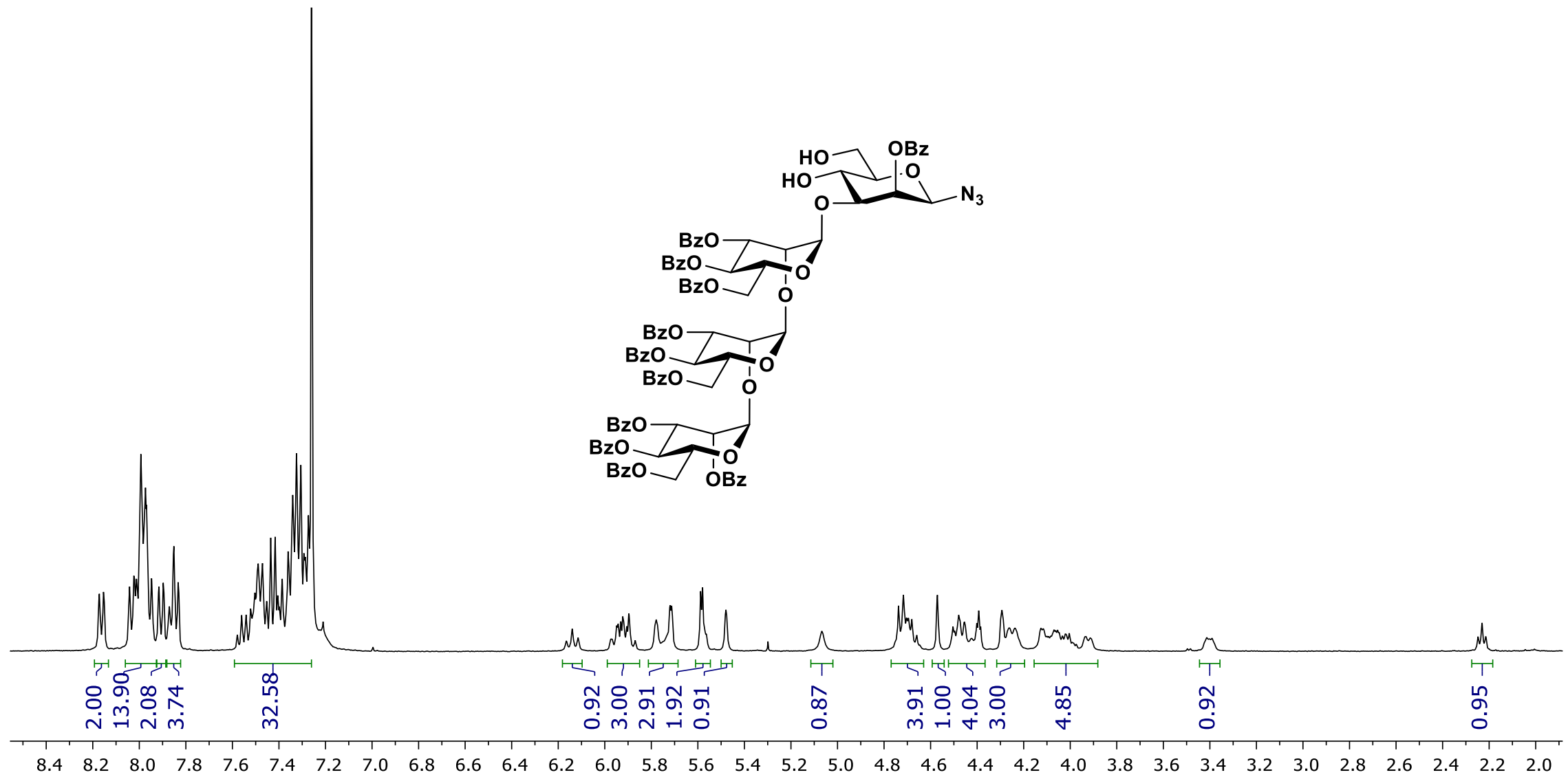

Figure S21. 1D ^{1}H NMR spectrum of **16** (400 MHz, $CDCl_3$).

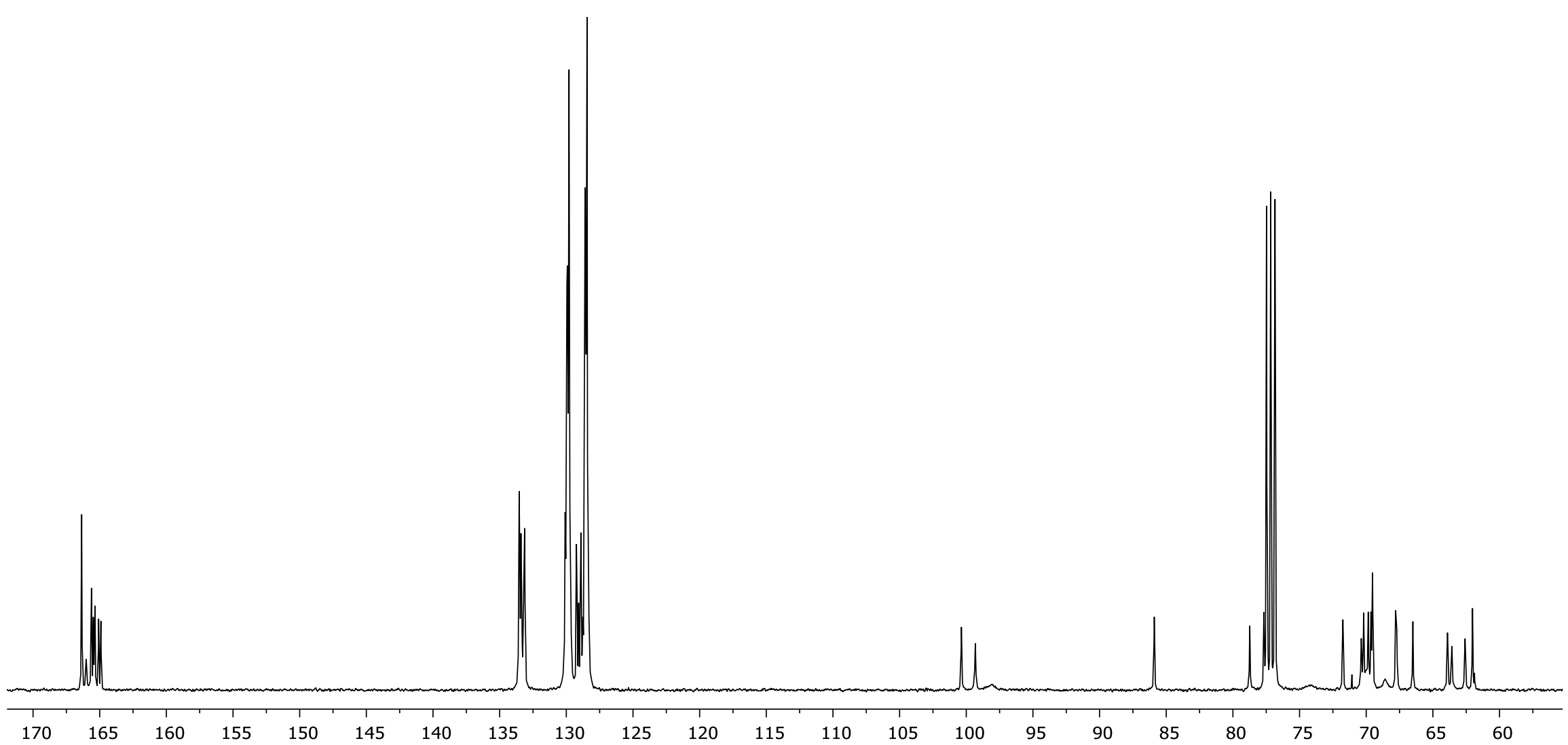

Figure S22. 1D $^{13}C\{^{1}H\}$ NMR spectrum of **16** (101 MHz, $CDCl_3$).

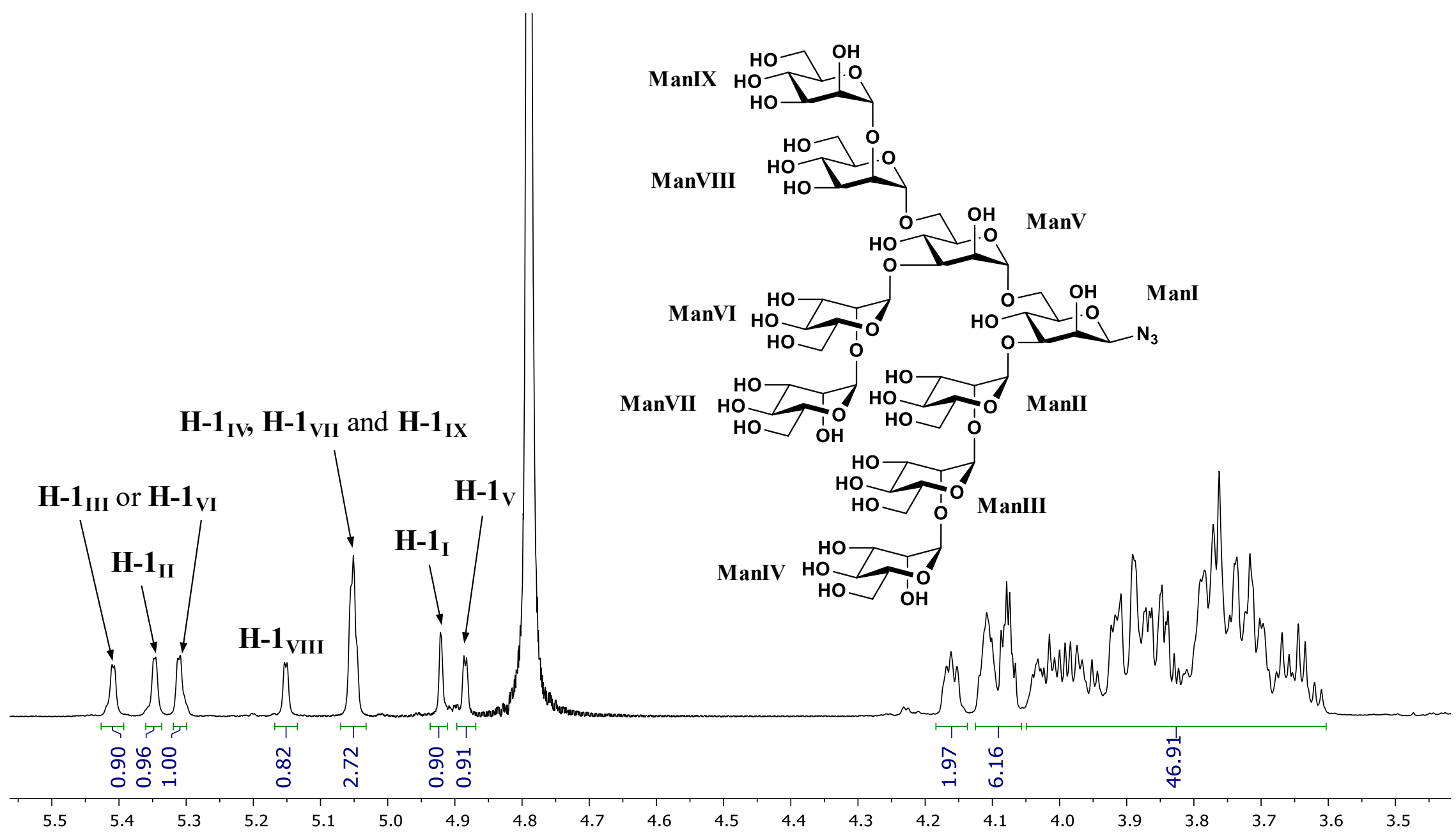

Figure S23. 1D ^{1}H NMR spectrum of **18** (400 MHz, D_2O).

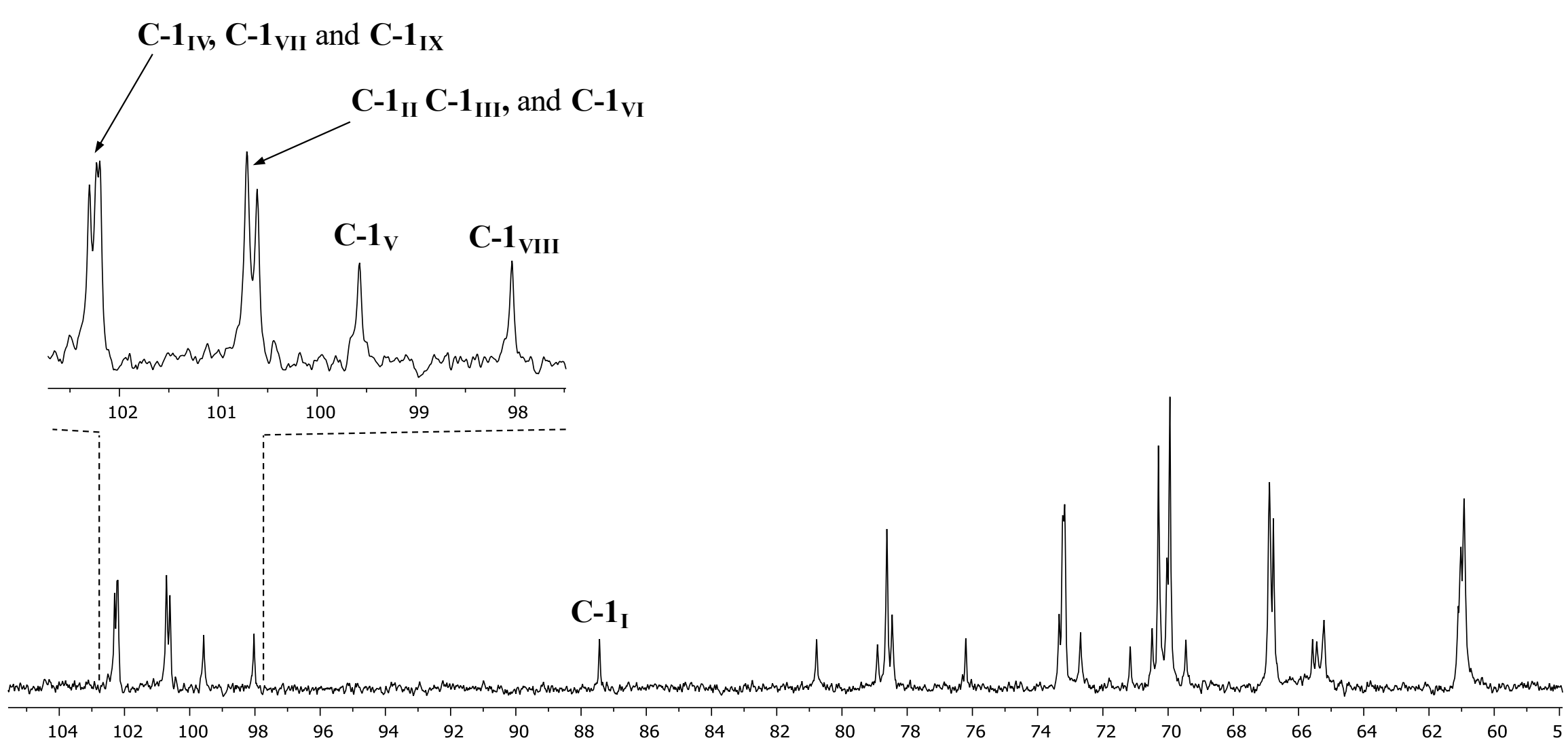

Figure S24. 1D ^{13}C{^{1}H} NMR spectrum of **18** (101 MHz, D_2O).

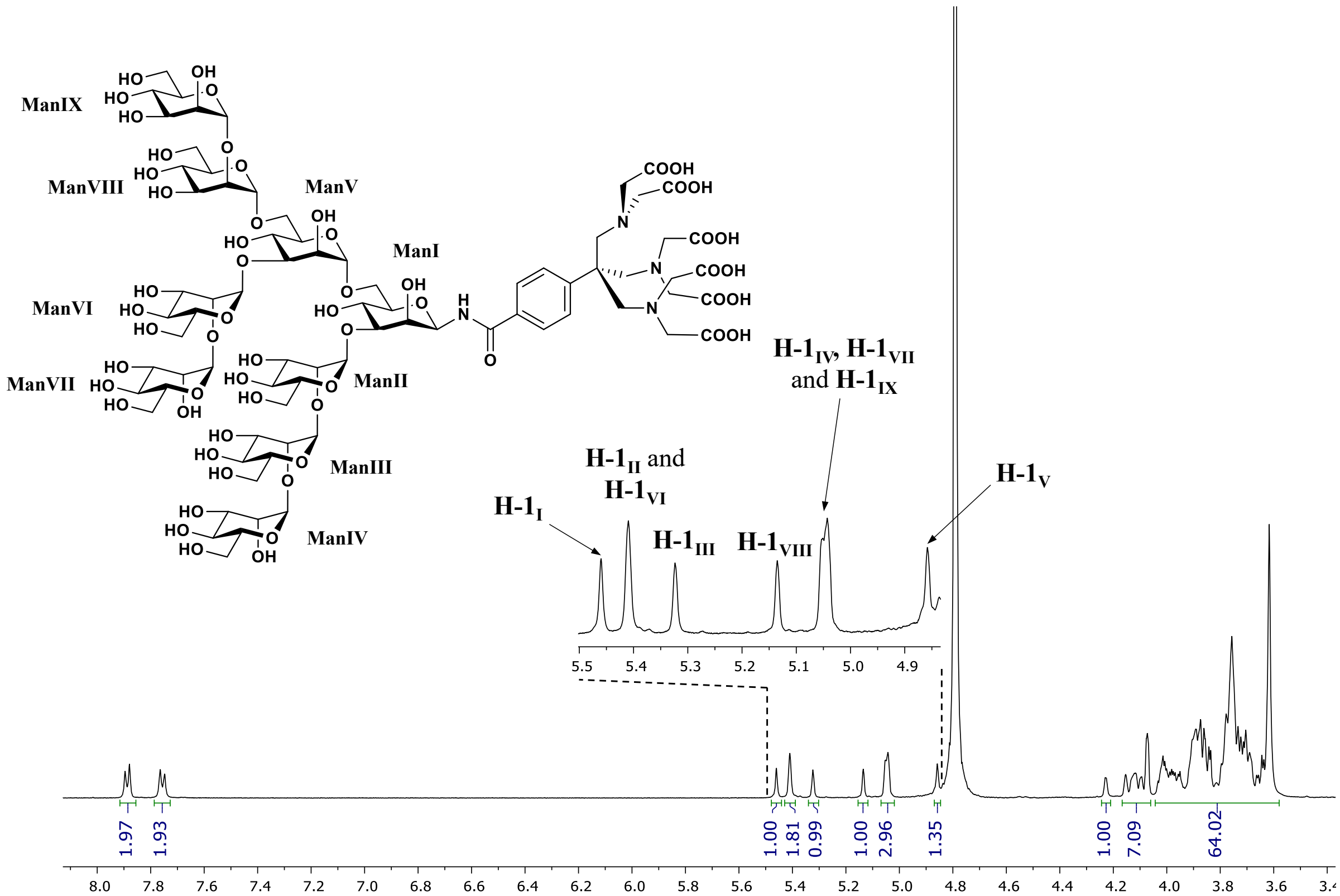

Figure S25. 1D ^{1}H NMR spectrum of **11** (500 MHz, D_2O).

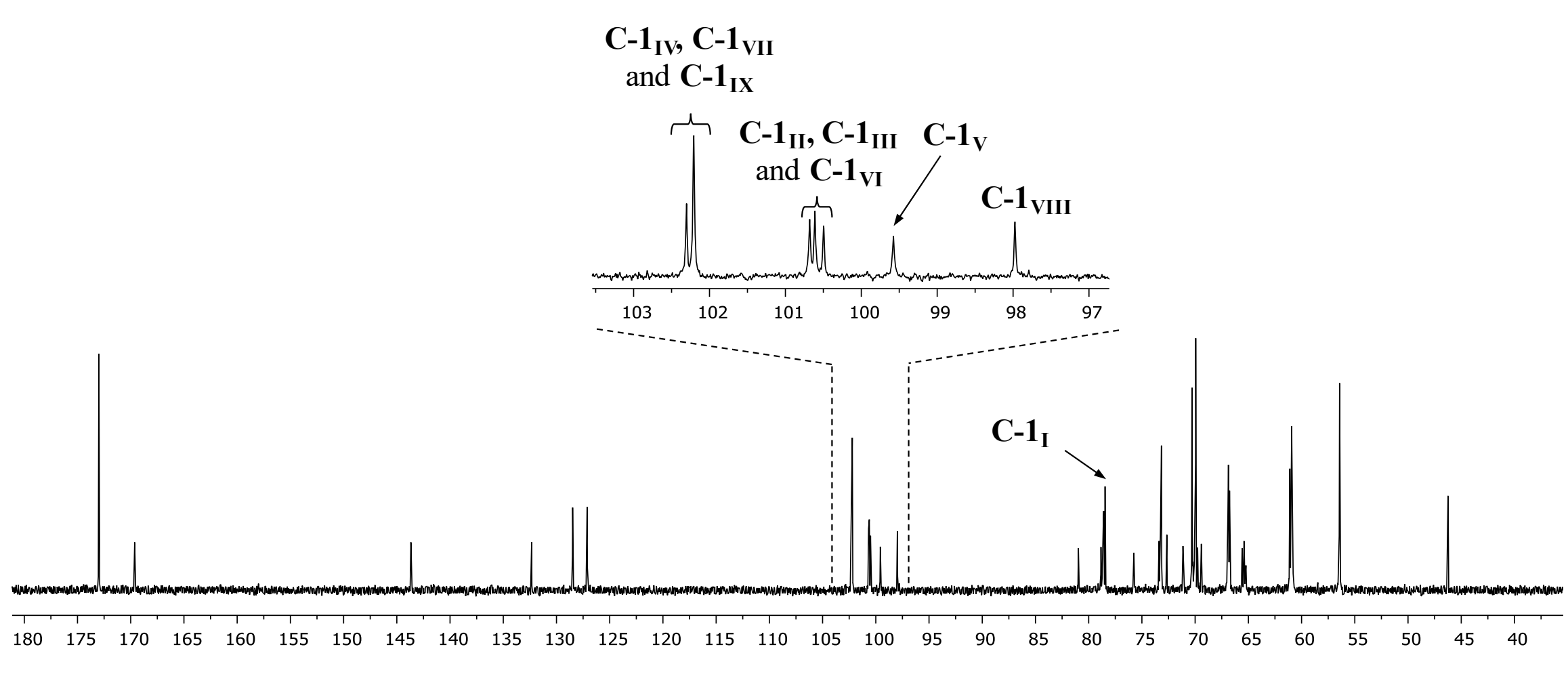

Figure S26. 1D $^{13}C\{^{1}H\}$ NMR spectrum of **11** (126 MHz, D_2O).

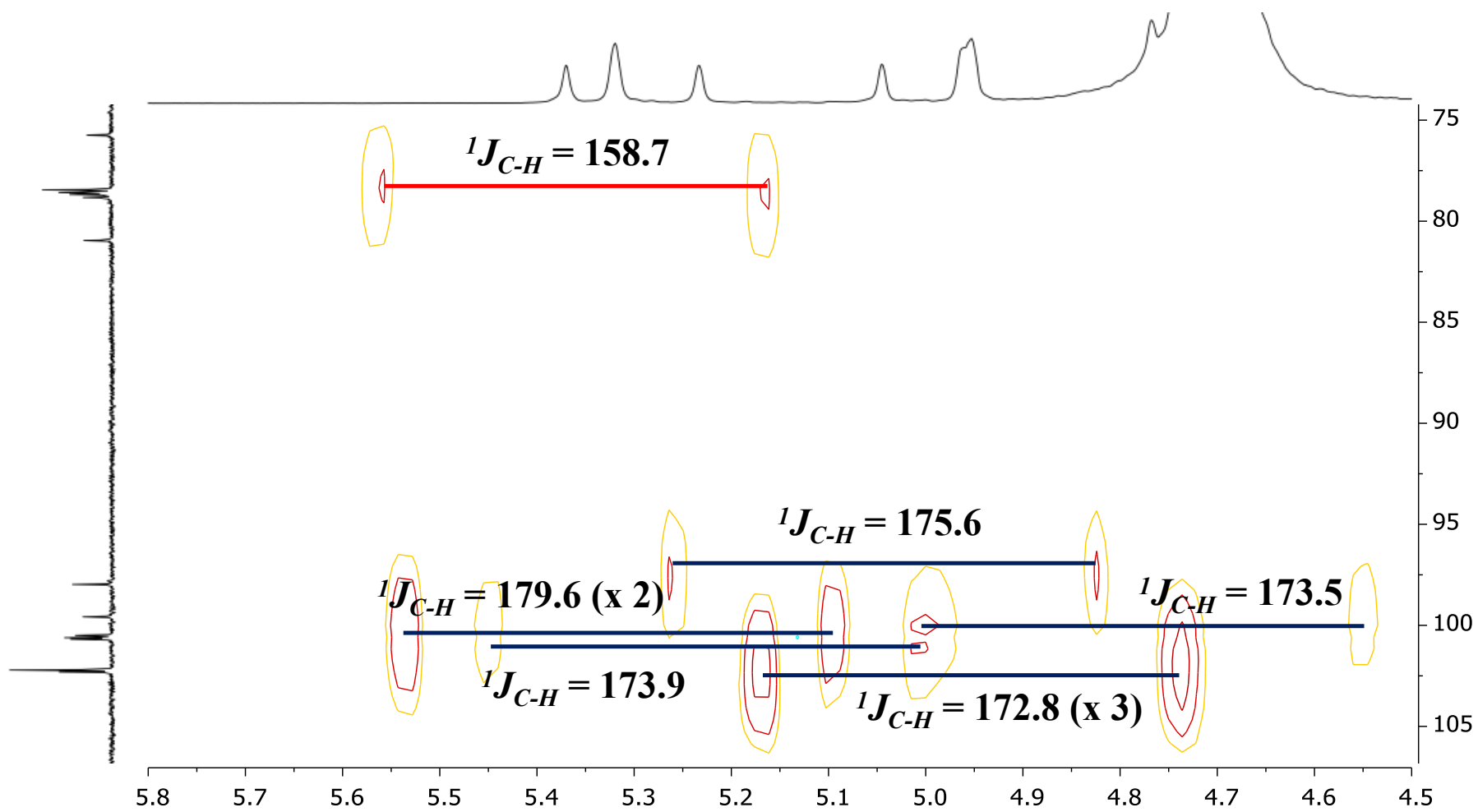

Figure S27. Coupled 2D ^{1}H-^{13}C HSQC NMR spectrum of **11** (500 MHz, D_2O).

4. Man_5/Man_9 interaction and competition studies

Experimental details

Samples containing 0.6 mM of Man_5 and 12 μM of 2G12 antibody, 50:1 Man5:2G12 molecular ratio (Polymun, lot number C2G12 090322A, product number AB002) in deuterated Tris-d_{11} buffer (50 mM Tris-d_{11}, NaCl 150 mM, pH 8.2) using D_2O as solvent were employed in the binding studies. Competition experiments were performed by adding Man_9 to the previously described Man_5 /2G12 sample to reach a final concentration of 0.6 mM of each oligomannoside (Man_5:Man_9:2G12, 50:50:1 molecular ratio). Control samples lacking the protein were also prepared in the same buffer, with Man_5 and Man_9 at concentrations of 0.6 mM and 1.0 mM, respectively.

For both control and protein-containing samples, ^1H STD NMR experiments were recorded using 3 mm NMR tubes on a Bruker AV 600 MHz NMR spectrometer equipped with a 5 mm cryoprobe with a maximum nominal z-gradient strength of 53.5 G cm^{-1} at 298 K and processed using the Bruker TopSpin software package (version 3.5.0). The experiments were acquired using the standard Bruker pulse program stddiffesgp with 4096 transients, 32,768 complex data points, a spectral width of 7.2 kHz (12 ppm), and an experiment time of 12 h. Solvent suppression at 4.7 ppm was achieved using 6 ms Squa100.1000 selective ^1H 180° radiofrequency pulses in the excitation sculpting element. A recovery delay of 3 s was used, and saturation was applied during 2 s using a train of 50 ms (power level of 50 dB) Gaus1.1000 selective ^1H 90° pulses at −0.1 ppm (on-resonance experiment) and at 100 ppm (off-resonance experiment).

^1H STD NMR spectra were also recorded on a Bruker AVIII 700 MHz NMR spectrometer equipped with a 5 mm cryoprobe with a maximum nominal z gradient strength of 53.5 G cm^{-1} at 298 K, and processed using the Bruker Topspin software package (version 3.5.0.). ^1H STD NMR spectra were acquired using the standard Bruker pulse program *stddiffesgp* with 4096 transients, 32768 complex data points, a spectral width of 10.5 kHz (15 ppm), and an experiment time of 10 h. 6 ms (and power level of 0.08 mW, 40.9 dB) Squa100.1000 selective ^1H 180° radiofrequency pulses were used in the excitation sculpting element to suppress the solvent signal at 4.7 ppm. A recovery delay of 3 s was used, saturation was applied during 2 s using a train of 50 ms (and power level of 0.01 mW, 50 dB) Gaus1.1000 selective ^1H 90° at −0.1 ppm (on-resonance experiment) and at 100 ppm (off-resonance experiment).

Figure S28 shows the 600 MHz STD ^{1}H NMR control spectrum of Man_5; no STD signals were detected in the absence of the protein.

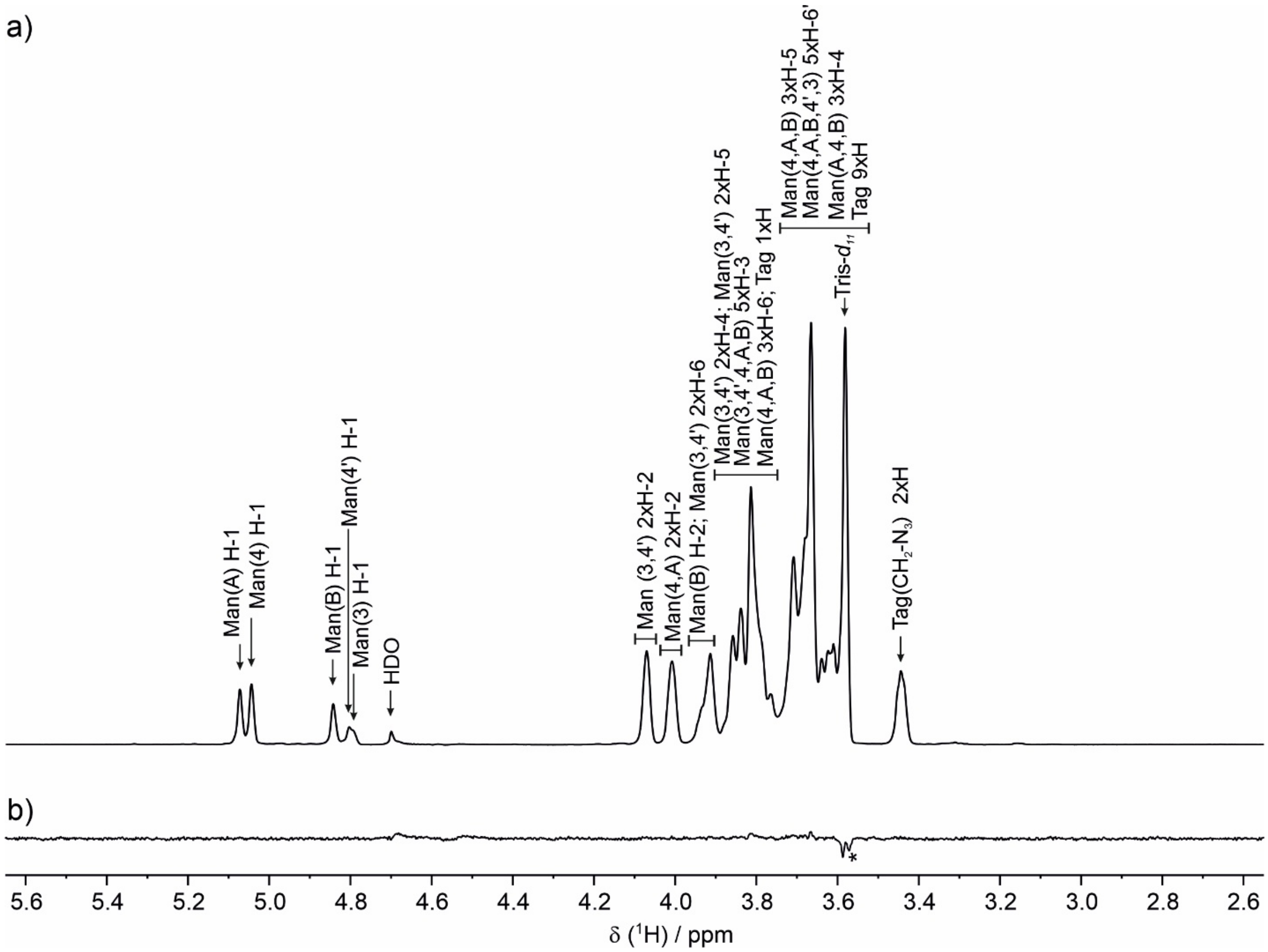

Figure S28. 600 MHz a) off-resonance and b) STD ^{1}H NMR spectrum of Man_5. Asterisk indicates a subtraction artefact from the Tris-d_{11} buffer signals.

Figure S29 shows the 600 MHz STD ^{1}H NMR spectrum of Man_5 in the presence of 2G12, revealing the interaction between 2G12 antibody and the oligomannoside.

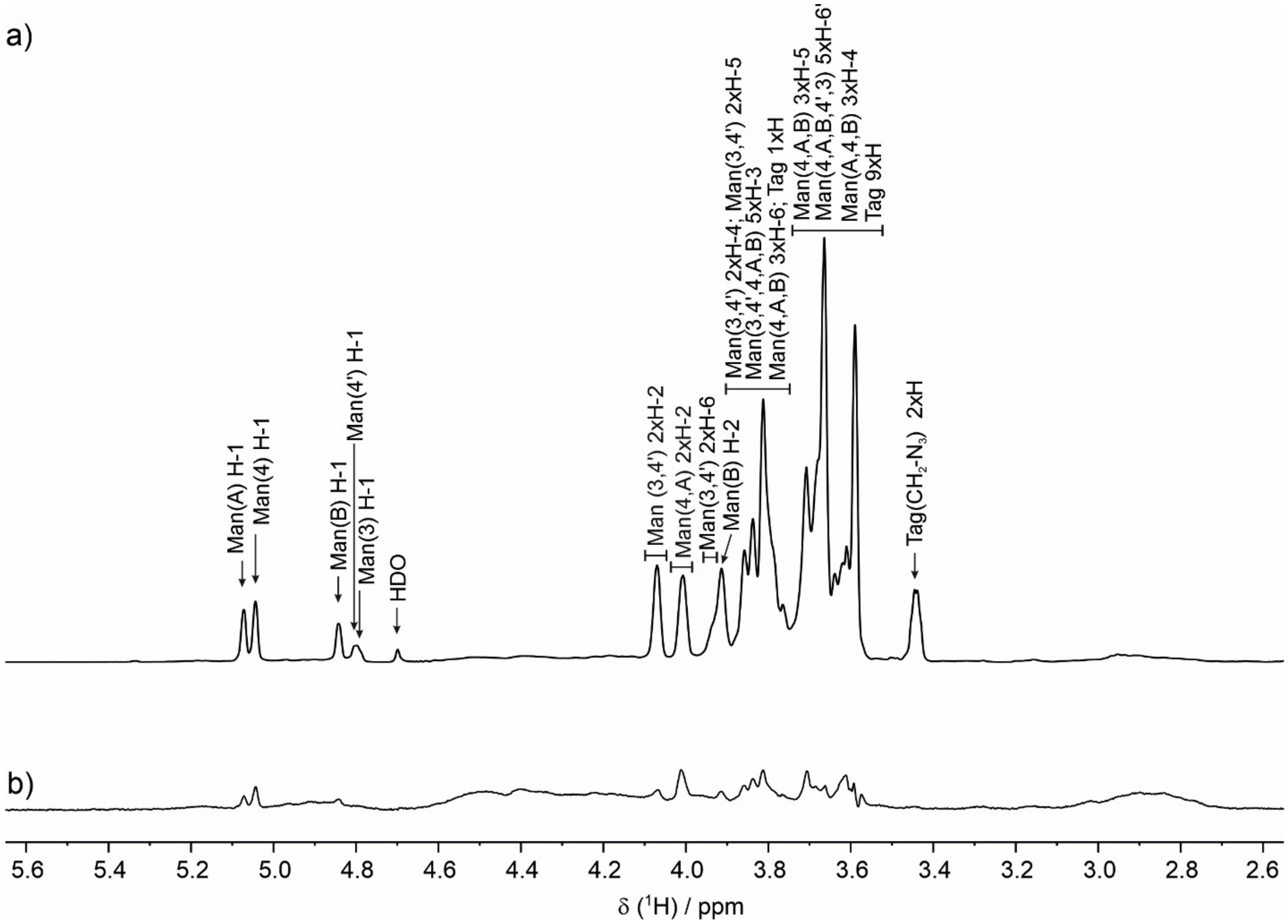

Figure S29. 600 MHz a) off-resonance and b) STD ^{1}H NMR spectrum of Man_5 in the presence of 2G12.

Figure S30 shows the 600 MHz STD ^{1}H NMR control spectrum of Man_9; no STD signals were detected in the absence of the protein.

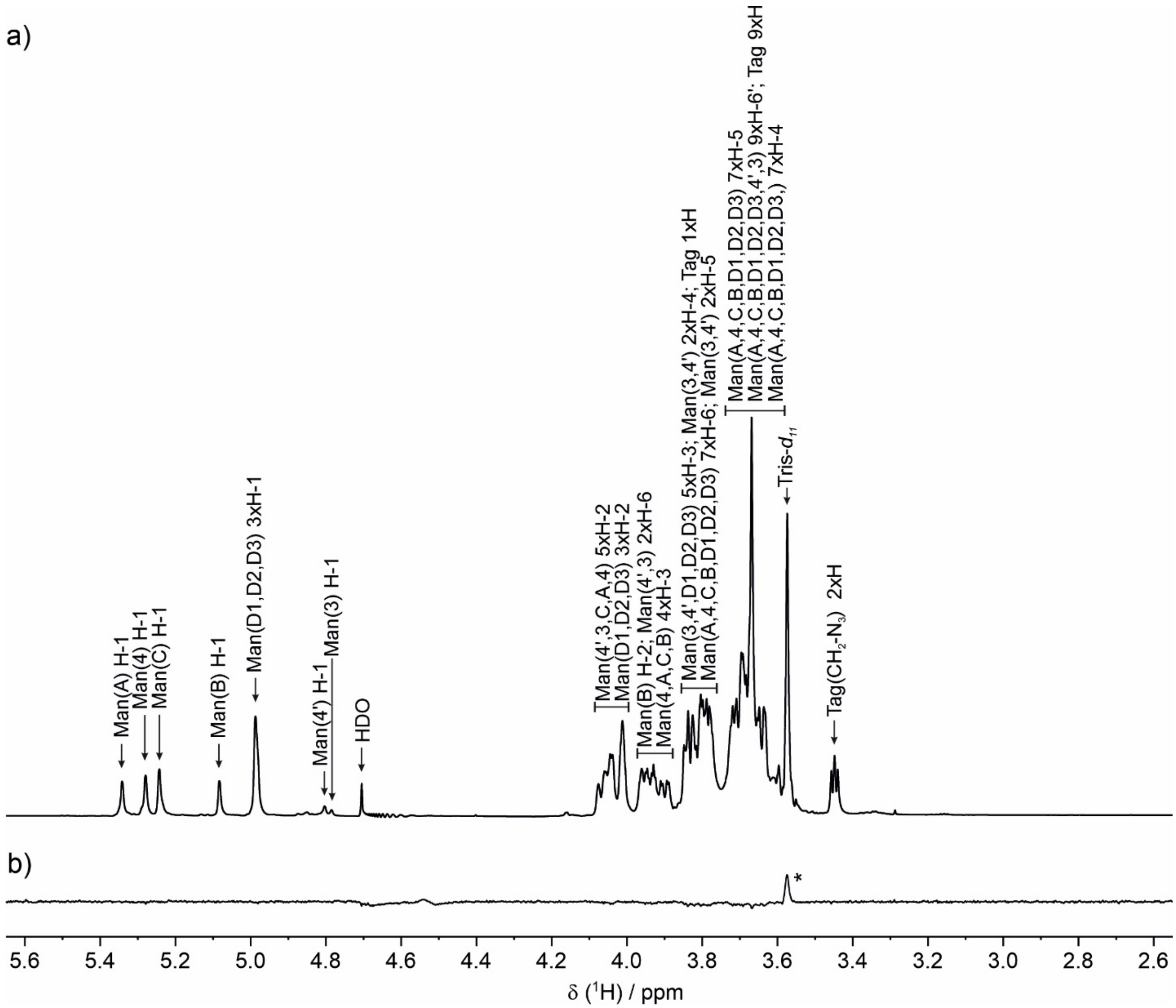

Figure S30. 600 MHz a) off-resonance and b) STD ^{1}H NMR spectrum of Man_9. * Asterisk indicates a subtraction artefact from the Tris-d_{11} buffer signals.

Figure S31 shows the 600 MHz STD ^{1}H NMR full spectra of Man_5 in the presence of the 2G12 upon addition of Man_9 acquired for monitoring the competition of both oligosaccharides for 2G12 binding. No STD signals were detected for the polyethylene glycol tag.

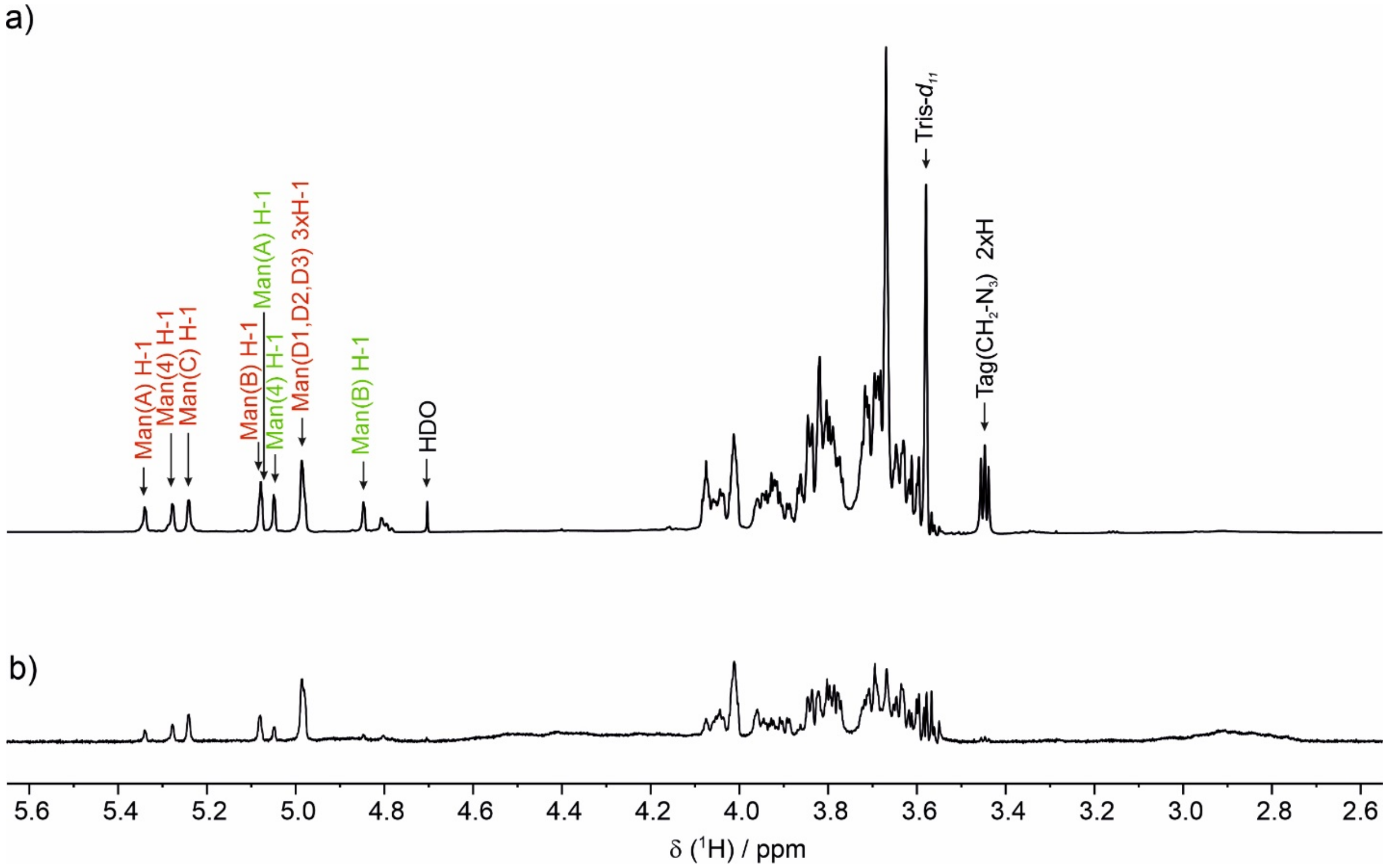

Figure S31. 600 MHz a) off resonance and b) STD ^{1}H NMR spectrum of Man_5/Man_9 sample in the presence of 2G12. Signals of Man_5 and Man_9 are highlighted in green and red, respectively.

Figure S32 shows the 700 MHz STD ^{1}H NMR full spectra of Man_5 in the presence of the 2G12 illustrating the interaction between the oligosaccharide and the antibody. No STD signals was detected for the polyethylene glycol tag.

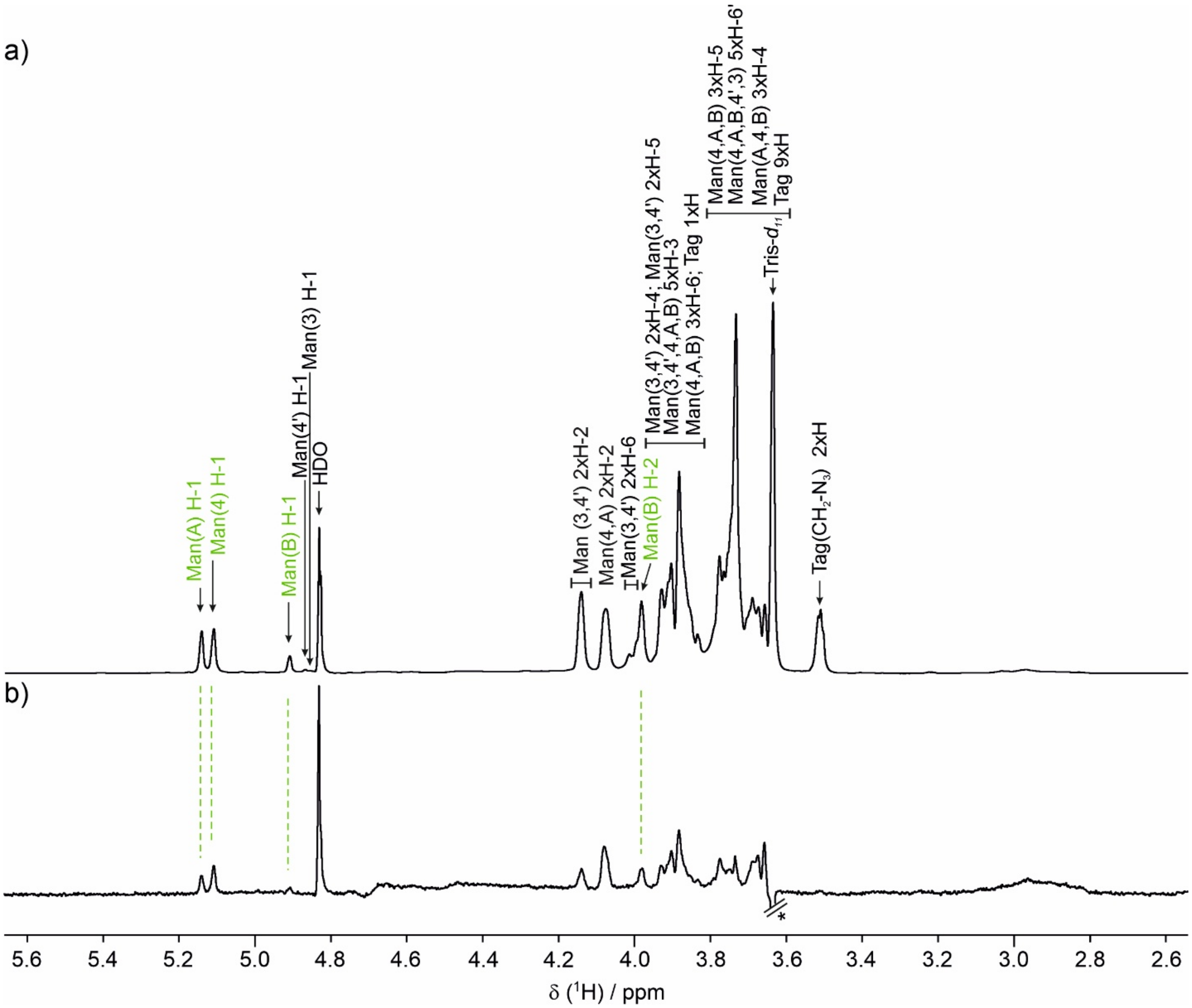

Figure S32. 700 MHz a) off resonance and b) STD ^{1}H NMR spectrum of Man_5 in the presence of 2G12. Signals that are not overlapped in the STD spectrum are highlighted in green. Asterisk indicates a subtraction artefact from the Tris-d_{11} buffer signals.

Figure S33 shows the 700 MHz STD ^{1}H NMR full spectra of Man_5 in the presence of the 2G12 upon addition of Man_9 acquired for monitoring the competition of both oligosaccharides for 2G12 binding. No STD signals were detected for the polyethylene glycol tag.

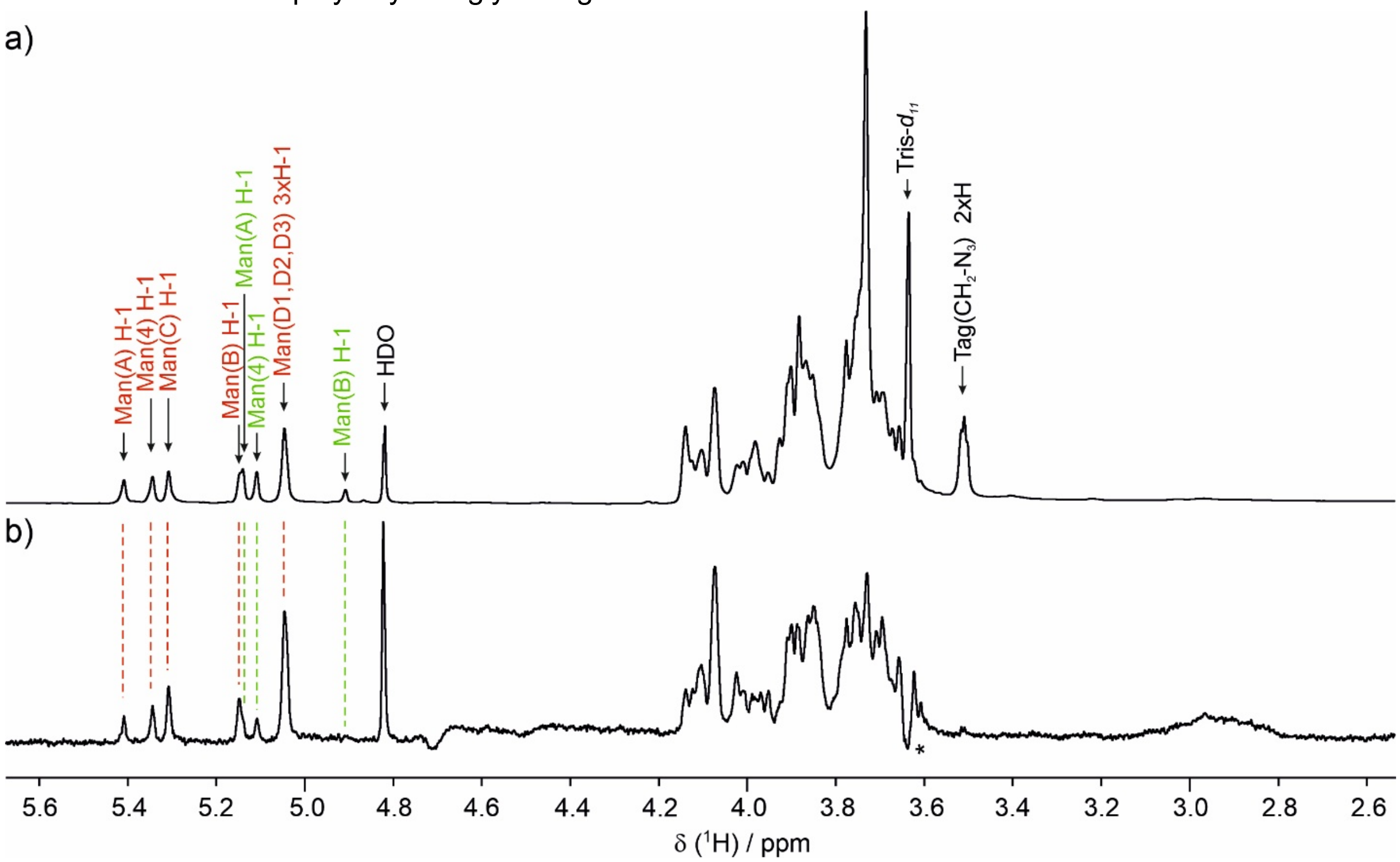

Figure S33. 700 MHz a) off-resonance and b) STD ^{1}H NMR spectrum the Man_5/2G12 sample upon addition of Man_9. Signals of Man_5 and Man_9 are highlighted in green and red, respectively. Asterisk indicates a subtraction artefact from the Tris-d_{11} buffer signals.

5. LBT–Man_9 interaction studies

Experimental details

A sample containing 1 mM of LBT–Man_9 loaded with Dy^{3+} and 20 μM 2G12 (Polymun, lot number C2G12 090322A, product number AB002) in deuterated Tris-d_{11} buffer (50 mM Tris-d_{11}, NaCl 150 mM, pH 8.2) using D_2O as solvent was used for the binding studies.

NMR spectra were recorded using a 3 mm NMR tube on a Bruker 600 MHz NMR spectrometer equipped with a 5 mm cryoprobe with a maximum nominal z gradient strength of 53.5 G cm^{-1} at 298 K, and processed using the Bruker Topspin software package (version 3.5). All ^{1}H STD NMR spectra shown in the main manuscript and SI were acquired using the standard Bruker pulse program *stddiffesgp* with 8192 transients, 32768 complex data points, a spectral width of 8.4 kHz (14 ppm), and an experiment time of 20 h. 3.5 ms (and 43.4 dB attenuation of power level) Squa100.1000 selective ^{1}H 180° radiofrequency pulses were used in the excitation sculpting element to suppress the solvent signal at 4.7 ppm. A recovery delay of 3 s was used, saturation was applied during 2 s using a train of 50 ms (and power level of 50 dB) Gaus1.1000 selective ^{1}H 90° pulses, at −0.1 ppm (on-resonance experiment) and at 100 ppm (off-resonance experiment). Prior to Fourier transformation, an exponential apodization with LB of 1 Hz was used.

^{1}H STD NMR spectra

Figure S34 shows the full STD ^{1}H NMR spectra of LBT-Man$_9$ loaded with dysprosium in the presence of 2G12 which exhibits a greater signal dispersion due to the presence of the paramagnetic ion.

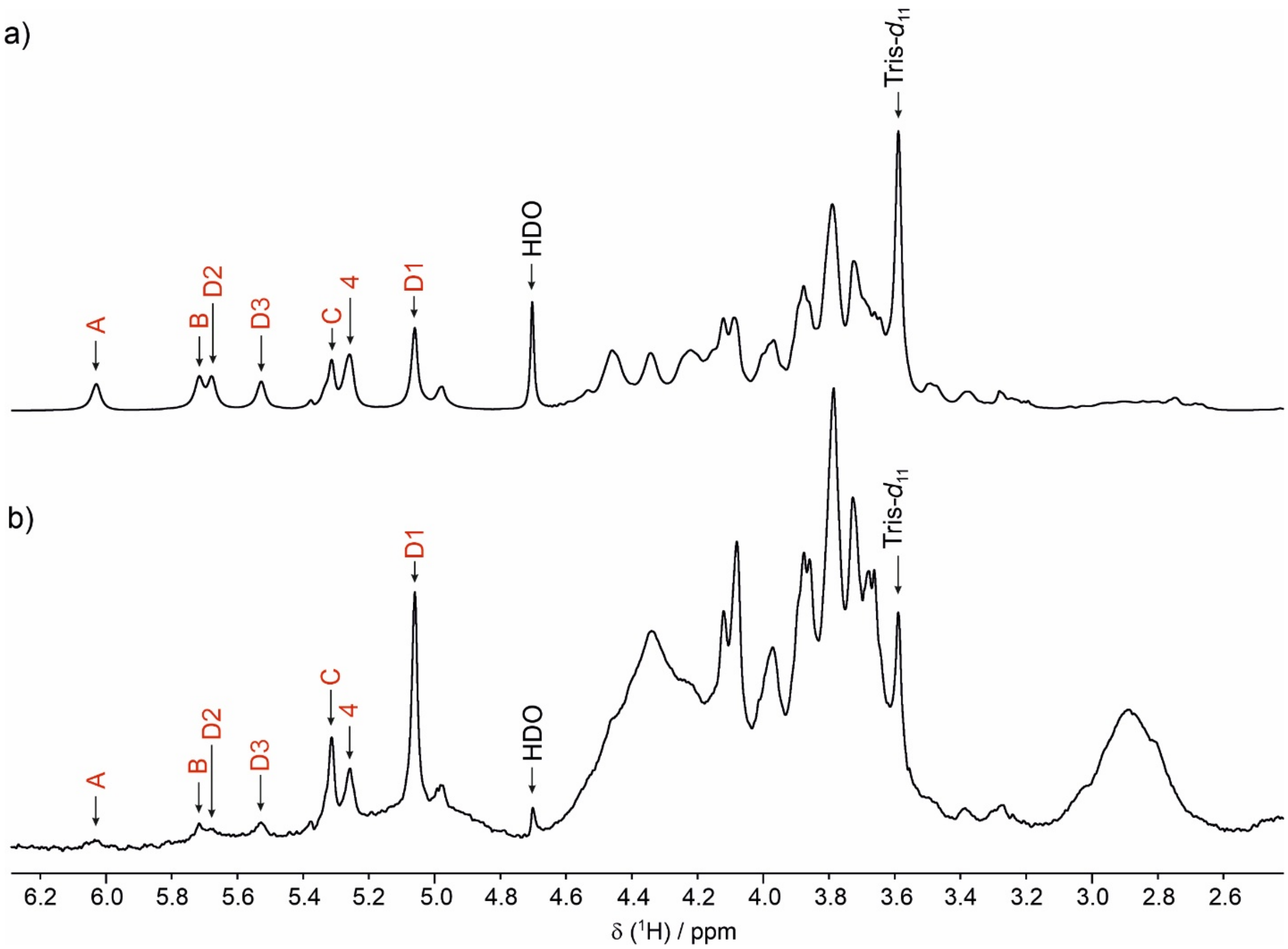

Figure S34. 600 MHz a) off resonance and b) STD ^{1}H NMR spectrum of LBT-Man$_9$ loaded with Dy^{3+} in the presence of 2G12. Signals that were used in the STD analysis are highlighted in red. The 4′ H1 signal is weak under paramagnetic conditions due to paramagnetic relaxation enhancement induced by the dysprosium ion and is not observed in the presence of the protein due to additional line broadening upon binding.

6. Man$_9$-LBT pseudo contact shifts measurement

Experimental details

Two samples were prepared for the measurement of pseudocontact shifts (PCSs). The reference sample contained 1 mM LBT-Man$_9$ in deuterated Tris-d$_{11}$ buffer (25 mM Tris-d$_{11}$, 150 mM NaCl, pH 8.2), using D_2O as the solvent. $LaCl_3$ was added to a final concentration of 1 mM, corresponding to a 1:1 molar ratio of $LaCl_3$ to LBT-Man$_9$. The paramagnetic sample was prepared in the same manner, substituting $LaCl_3$ with $DyCl_3$ at an identical final concentration of 1 mM.

NMR spectra were recorded using a 3 mm NMR tube on a Bruker AVIII 700 MHz NMR spectrometer equipped with a 5 mm cryoprobe with a maximum nominal z gradient strength of 53.5 G cm^{-1} at 298 K, and

processed using the Bruker Topspin software package (version 3.5). 2D ^{1}H-^{13}C HSQC spectra for both diamagnetic (La^{3+}) and paramagnetic (Dy^{3+}) samples were acquired the standard Bruker pulse program *hsqcetgpsisp.2* with 48 transients, 2048 complex data points, 256 increments, a ^{1}H spectral width of 7 kHz (10 ppm), a ^{13}C spectral width of 21.1 kHz (120 ppm), and an experiment time of 4 h. The experiments were optimised to observed $^{1}J_{CH}$ of 145 Hz and the sensitivity improvement delay was set to $1/8^{1}J_{CH}$ for improving all multiplicities. Prior to Fourier transformation, zero-filling to 4096 and 1024 complex points was applied in ^{1}H and ^{13}C, respectively and a QSINE apodization with SSB 2 for both dimensions.

Pseudo Contact Shifts (PCSs) were calculated as the difference in ^{1}H chemical shift measured between diamagnetic and paramagnetic 2D ^{1}H-^{13}C HSQC spectra. The peak assignments were confirmed by using 2D ^{1}H-^{13}C HMQC-TOCSY, 2D ^{1}H-^{1}H TOCSY and 2D ^{1}H-^{1}H NOESY experiments (data not shown).

2D ^{1}H-^{13}C HSQC NMR spectra

Figure S35 shows two 2D ^{1}H–^{13}C HSQC spectra overlapped, corresponding to the sample containing LBT-Man_9 loaded with lanthanum (in red) and with dysprosium (in blue), highlighting the increased signal dispersion induced by the paramagnetic dysprosium ion. The signals over 7.5 ppm in the red spectrum correspond to the aromatic ring of the lanthanide binding tag structure. These signals cannot be detected in paramagnetic conditions (blue spectrum) due to the paramagnetic relaxation enhancement effect of dysprosium. Residual signals of unbound glycan were observed in the dysprosium spectrum (in blue) at low intensity level; therefore, most of them cannot be seen at the signal intensity plotted in Figures S35 and in Figure 3 of the main manuscript. In Figure 3, the residual signal of the three anomeric protons H1 D1, H1 D2, and H1 D3, which are overlapped in the unbound glycan, can be seen as a weak peak below the signal of the same protons in the lanthanum spectrum. To avoid nonspecific line broadening due to metal excess, no additional dysprosium was added to the sample.

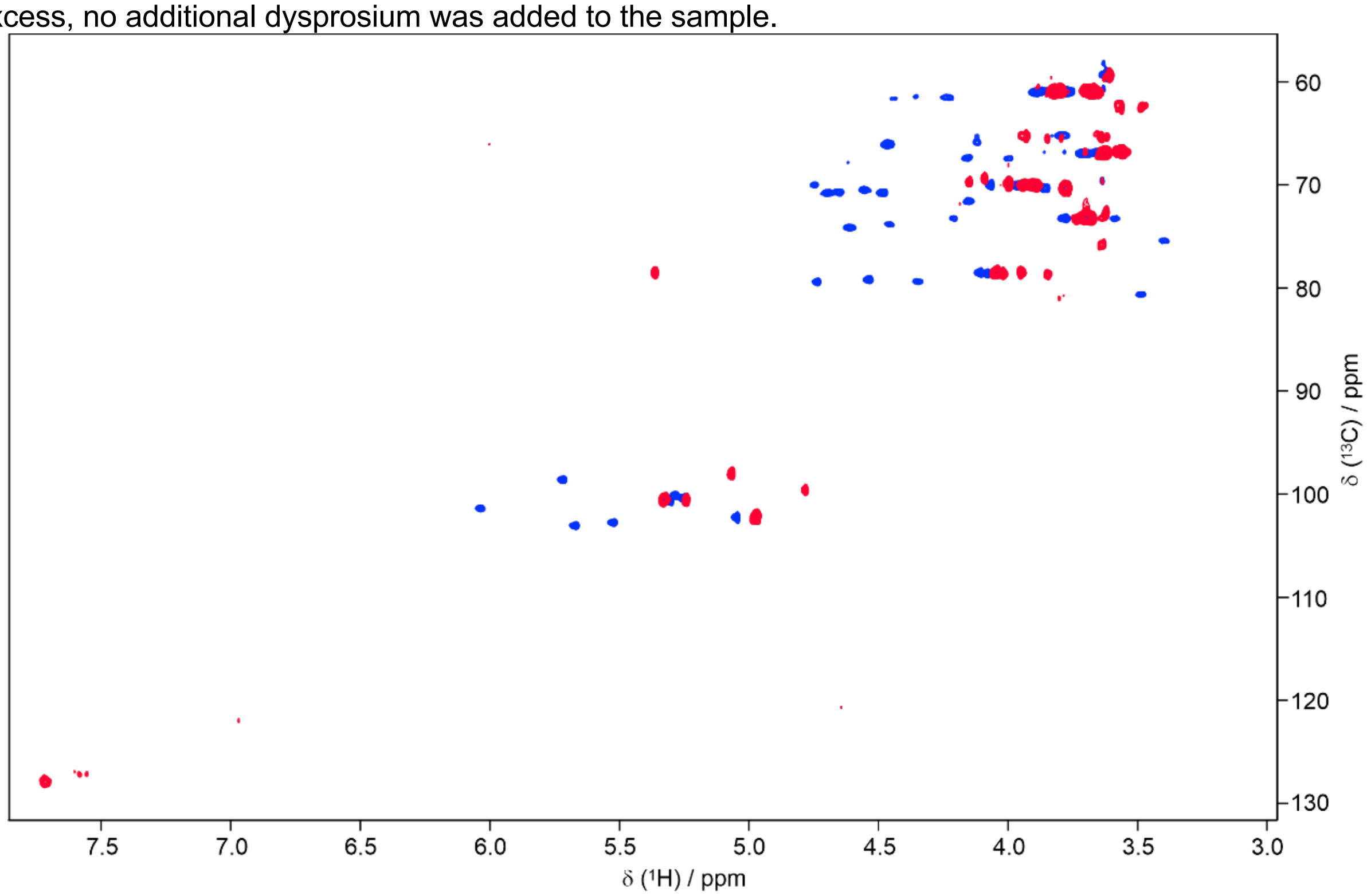

Figure S35. Overlaid of the 2D ^{1}H–^{13}C HSQC spectrum of LBT-Man_9 loaded with La^{3+} (in red) and the same experiment of the LBT-Man_9 loaded with Dy^{3+} (in blue).

Man_9-LBT-La^{3+} and Man_9-LBT-Dy^{3+} NMR assignment

Table S1. Man_9-LBT-La^{3+} NMR assignment at 298 K.

Man3		
Proton	δ (^{1}H) / ppm	δ (^{13}C) / ppm
H1	5.36	78.5
H2	4.14	69.7
H3	3.78	80.9
H4	3.78	65.5
H5	3.63	75.7
H6/H6'	3.94/3.63	65.2

ManB		
Proton	δ (^{1}H) / ppm	δ (^{13}C) / ppm
H1	5.05	98.0
H2	3.94	78.6
H3	3.88	70.0
H4	3.62	66.9
H5	3.69	73.2
H6/H6'	3.80/3.68	60.9

Man4		
Proton	δ (^{1}H) / ppm	δ (^{13}C) / ppm
H1	5.31	100.5
H2	4.04	78.6
H3	3.94	70.1
H4	3.62	66.9
H5	3.77	71.1
H6/H6'	3.80/3.68	60.9

ManA		
Proton	δ (^{1}H) / ppm	δ (^{13}C) / ppm
H1	5.33	100.6
H2	4.01	78.5
H3	3.88	70.0
H4	3.62	66.9
H5	3.69	73.2
H6/H6'	3.80/3.68	60.9

Man4'		
Proton	δ (^{1}H) / ppm	δ (^{13}C) / ppm
H1	4.77	99.6
H2	4.08	69.4
H3	3.83	78.8
H4	3.84	65.4
H5	3.77	71.1
H6/H6'	3.92/3.63	65.4

ManC		
Proton	δ (^{1}H) / ppm	δ (^{13}C) / ppm
H1	5.23	100.6
H2	4.03	78.4
H3	3.88	70.0
H4	3.62	66.9
H5	3.69	73.2
H6/H6'	3.80/3.68	60.9

ManD1D2D3		
Proton	δ (^{1}H) / ppm	δ (^{13}C) / ppm
H1	4.97	102.2
H2	3.99	69.9
H3	3.77	70.3
H4	3.56	66.8
H5	3.69	73.2
H6/H6'	3.81/3.65	61.1

LBT		
Proton	δ (^{1}H) / ppm	δ (^{13}C) / ppm
Ph-o	7.71	127.9
Ph-m	7.58	127.1
CH_2a		
CH_2b		

Table S2. Man_9-LBT-Dy^{3+} NMR assignment at 298 K.

**Man3**		
Proton	δ (^{1}H) / ppm	δ (^{13}C) / ppm
H1	4.24	77.2
H2		
H3	3.48	80.6
H4		
H5	3.40	75.4
H6/H6'		

**ManB**		
Proton	δ (^{1}H) / ppm	δ (^{13}C) / ppm
H1	5.70	98.6
H2	4.53	79.2
H3		
H4		
H5		
H6/H6'		

**Man4**		
Proton	δ (^{1}H) / ppm	δ (^{13}C) / ppm
H1	5.24	100.4
H2		
H3		
H4		
H5		
H6/H6'		

**ManA**		
Proton	δ (^{1}H) / ppm	δ (^{13}C) / ppm
H1	6.03	101.4
H2	4.73	79.4
H3		
H4		
H5		
H6/H6'		

**Man4'**		
Proton	δ (^{1}H) / ppm	δ (^{13}C) / ppm
H1	5.28	100.1
H2		
H3		
H4	4.34	79.4
H5	4.14	71.6
H6/H6'		

**ManC**		
Proton	δ (^{1}H) / ppm	δ (^{13}C) / ppm
H1	5.30	100.6
H2		
H3		
H4	3.68	66.9
H5		
H6/H6'		

**ManD1**		
Proton	δ (^{1}H) / ppm	δ (^{13}C) / ppm
H1	5.05	102.2
H2	4.06	70,0
H3	3.84	70.3
H4		
H5		
H6/H6'		

**ManD2**		
Proton	δ (^{1}H) / ppm	δ (^{13}C) / ppm
H1	5.67	103.0
H2	4.65	70.7
H3		
H4		
H5		
H6/H6'		

**ManD2**		
Proton	δ (^{1}H) / ppm	δ (^{13}C) / ppm
H1	5.53	102.7
H2	4.55	70.5
H3		
H4		
H5		
H6/H6'		

**LBT**		
Proton	δ (^{1}H) / ppm	δ (^{13}C) / ppm
Ph-o		
Ph-m		
CH_2a		
CH_2b		

7. Molecular dynamics simulations

System preparation

Four distinct conformations of the Man_9 glycan were constructed using GLYCAM carbohydrate builder[7] to represent the different combinations of extended and folded orientations of its two main glycosidic arms (α1–3 and α1–6): extended–extended, extended–folded, folded–extended, and folded–folded. Each initial structure was manually assembled in the correct topology using tleap from the AMBER20 package,[8] with glycosidic linkages defined explicitly via atom-specific bonding commands. The GLYCAM06-j1 force field[9] was used to parameterize all carbohydrate residues, and TIP3P water molecules were used for explicit solvation. The system was embedded in an octahedral water box extending 15 Å sides from the solute.

Energy minimization, heating, and equilibration

Energy minimization was performed in two stages. In the first stage, solvent molecules were relaxed with harmonic restraints applied to the glycan heavy atoms (force constant = 500 $kcal \cdot mol^{-1} \cdot Å^{-2}$). In the second stage, the entire system was minimized without restraints. Each minimization step included 99,999 cycles with a convergence criterion of 0.02 $kcal \cdot mol^{-1} \cdot Å^{-1}$ and a non-bonded cutoff of 15 Å.

The minimized system was gradually heated from 0 K to 298 K over 200 ps under constant volume conditions (NVT), employing a Langevin thermostat and harmonic restraints of 20 $kcal \cdot mol^{-1} \cdot Å^{-2}$ on the glycan. This was followed by 100 ps of density equilibration under constant pressure (NPT) using isotropic pressure coupling at 1 atm. A multi-step equilibration protocol followed, consisting of four sequential 175 ps simulations (700 ps total) under NPT conditions. During this phase, restraints on the glycan were progressively decreased (20 → 10 → 5 → 2.5 $kcal \cdot mol^{-1} \cdot Å^{-2}$), allowing the glycan to relax gradually while maintaining structural integrity.

Production Simulations

All production simulations were performed in the NPT ensemble at 298 K and 1 atm, with a 2 fs integration time step. The Langevin thermostat (γ = 3.0 ps^{-1}) and isotropic pressure coupling (τ_p= 2.0 ps) were used throughout. Long-range electrostatics were treated using Particle Mesh Ewald (PME) with a real-space cutoff of 8 Å. All hydrogen-containing bonds were constrained using SHAKE. Each production trajectory was propagated for 100 ns (50,000,000 steps), saving coordinates and energy data every 5,000 steps.

For each of the four initial conformations, three independent 100 ns trajectories were run to assess convergence. Additionally, a fourth trajectory of 900 ns was performed for each conformation to ensure exhaustive sampling and enable high-resolution analysis of glycosidic torsion angles. In total, this yielded 1.2 μs of sampling per conformation and 4.8 μs overall.

Dihedral Angle Analysis

Torsional analysis focused on key glycosidic dihedrals governing the orientation of the α1–3 and α1–6 branches. Specific φ, ψ (and in some cases ω) dihedral definitions were manually assigned based on the glycan topology and residue numbering using cpptraj from AmberTools. Time series of dihedral angles were extracted from the 900 ns trajectories.

Two-dimensional (2D) histograms of the torsion angles were generated using custom Python scripts, which binned the angle pairs into a 360×360 grid covering the full dihedral range (–180° to 180°). The resulting distributions were visualized using in-house plotting tools to produce density maps and contour plots for structural analysis.

8. φ / ψ / ω angle distributions for Man_9

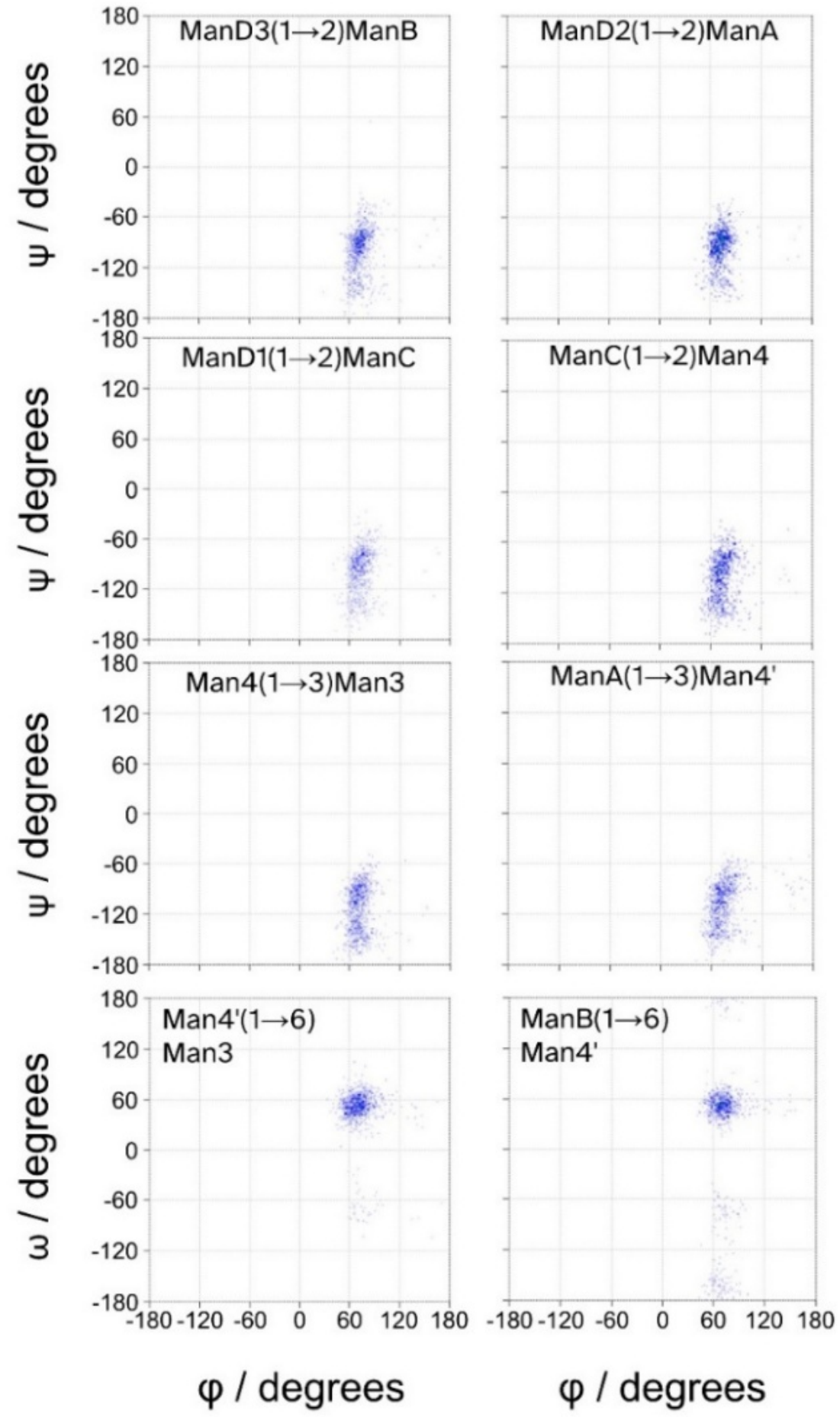

$1{\to}6$: $\varphi = O_5\text{-}C_1\text{–}O_6\text{-}C_6$; $\omega = C_4\text{-}C_5\text{-}C_6\text{-}O_6$
$1{\to}3$: $\varphi = O_5\text{-}C_1\text{–}O_3\text{-}C_3$; $\psi = C_1\text{-}O_3\text{-}C_3\text{-}C_2$
$1{\to}2$: $\varphi = O_5\text{-}C_1\text{–}O_2\text{-}C_2$; $\psi = C_1\text{-}O_2\text{-}C_2\text{-}C_1$

Figure S36. Torsional angles obtained by molecular dynamic simulations.

9. Conformational analysis using MSpin software

The lanthanide binding tag was added to the structures obtained by molecular dynamics simulations by using Schödinger Maestro2023-3.[10] The conformations were evaluated with MSpin software[11] to obtain the ensemble of conformations that better fit the experimental data. MSpin was used to back-calculate the expected PCSs values for the different conformations. The quality of the fitting between the experimental and calculated values is given by the Cornilescu Q factor, which is defined by the following expression:

$$Q = \sqrt{\frac{PCS_{calc}^2 - PCS_{exp}^2}{PCS_{exp}^2}}$$

Equation S1. Cornilescu Q factor

Different ensembles of conformations were considered in the calculations and the best correlation between experimental and calculated data were obtained when 1-6 glycosidic linkages adopt the gauche-gauche

conformation, ω (O6C6C5C4) ~60°. In the case of ψ1 and ψ2 angles, characteristic values of both extended (ψ ~180°) and folded conformations (ψ ~90°) were included in the calculations and the best correlation with the experimental data was obtained considering: folded/extended (ψ_1 ~90°, ψ_2 ~180) , extended/folded (ψ_1 ~180°, ψ_2 ~90), and folded/folded (ψ_1 ~90°, ψ_2 ~90) conformations with populations of 61%, 28% and 11%, respectively. The population weights were computed using the "Fit Populations" tool implemented in the MSpin software. The coordinates of a representative structure from each conformer family were considered in MSpin calculations.

Experimental and calculated PCSs used in the conformational analysis using MSpin

Table S3. Experimental PCS values (ppm) of Man_9LBT observed with Dy^{3+} and calculated PCSs obtained with MSpin software for the ensemble of conformations folded/extended, extended/folded, and folded/folded with populations of 61%, 28% and 11%, respectively.

	**Experimental ^{1}H PCS** (ppm)	**Calculated ^{1}H PCS** (ppm)
H1 Man3	1.12	1.03
H3 Man3	0.30	0.45
H5 Man3	0.23	0.27
H1 Man4	0.07	0.11
H1 Man4'	-0.51	-0.56
H3 Man4'	-0.51	-0.62
H5 Man4'	-0.37	-0.40
H1 ManB	-0.65	-0.51
H2 ManB	-0.59	-0.60
H1 ManA	-0.70	-0.63
H2 ManA	-0.72	-0.75
H1 ManC	-0.07	-0.07
H4 ManC	-0.06	-0.03
H1 ManD3	-0.56	-0.58
H2 ManD3	-0.56	-0.57
H1 ManD2	-0.70	-0.61
H2 ManD2	-0.66	-0.70
H1 ManD1	-0.08	-0.01
H2 ManD1	-0.07	-0.05
H3 ManD1	-0.07	-0.13

10. References

[1] I. Alves, B. Santos-Pereira, N. de la Cruz, A. Campar, V. Pinto, P. M. Rodrigues, M. Araújo, S. Santos, J. Ramos-Soriano, C. Vasconcelos, R. Silva, N. Afonso, F. Mira, C. C. Barrias, N. L. Alves, J. Rojo, L. Santos, A. Marinho, S. S. Pinho, *Sci. Transl. Med.* **2023**, *15*.

[2] P. H. Tam, T. L. Lowary, *Carbohydr. Res.* **2007**, *342*, 1741-1772.

[3] J. Ramos-Soriano, M. C. De La Fuente, N. De La Cruz, R. C. Figueiredo, J. Rojo, J. J. Reina, *Org. Biomol. Chem.* **2017**, *15*, 8877-8882.

[4] S. Di Pietro, V. Bordoni, D. Iacopini, S. Achilli, M. Pineschi, M. Thépaut, F. Fieschi, P. Crotti, V. Di Bussolo, *Bioorg. Chem.* **2021**, *107*.

[5] P. I. Abronina, V. V. Kachala, L. O. Kononov, *Carbohydr. Res.* **2009**, *344*, 240-244.

[6] K. Bock, C. Pedersen, I. Bock, I. Lundt, C. Pedersen.

[7] O. C. Grant, D. Wentworth, S. G. Holmes, R. Kandel, D. Sehnal, X. Wang, Y. Xiao, P. Sheppard, T. Grelsson, A. Coulter, G. Miller, B. L. Foley, R. J. Woods, **2025**.
[8] D. A. Case, K. Belfon, I. Y. Ben-Shalom, S. R. Brozell, D. S. Cerutti, T. E. Cheatham, III, V. W. D. Cruzeiro, T. A. Darden, R. E. Duke, G. Giambasu, M. K. Gilson, H. Gohlke, A. W. Goetz, R. Harris, S. Izadi, S. A. Izmailov, K. Kasavajhala, A. Kovalenko, R. Krasny, T. Kurtzman, T. S. Lee, S. LeGrand, P. Li, C. Lin, J. Liu, T. Luchko, R. Luo, V. Man, K. M. Merz, Y. Miao, O. Mikhailovskii, G. Monard, H. Nguyen, A. Onufriev, F. Pan, S. Pantano, R. Qi, D. R. Roe, A. Roitberg, C. Sagui, S. Schott-Verdugo, J. Shen, C. L. Simmerling, N. R. Skrynnikov, J. Smith, J. Swails, R. C. Walker, J. Wang, L. Wilson, R. M. Wolf, X. Wu, Y. Xiong, Y. Xue, D. M. York, P. A. Kollman, University of California, San Francisco, **2020**.
[9] K. N. Kirschner, A. B. Yongye, S. M. Tschampel, J. González-Outeiriño, C. R. Daniels, B. L. Foley, R. J. Woods, *J. Comput. Chem.* **2008**, *29*, 622-655.
[10] Schrödinger, New York, NY, **2023**.
[11] A. Navarro-Vázquez, *Magn. Reson. Chem.* **2012**, *50*, S73-S79.
